# People-centered strategies to mobilize people living with disabilities due to Neglected Tropical Diseases (PD-NTDs) to influence policy and programs: A mixed-methods study in Côte d’Ivoire

**DOI:** 10.1371/journal.pntd.0013485

**Published:** 2025-09-08

**Authors:** Julien Aké, Eunheh Koh, Tiembre Issiaka, Boko-Koiadia Adjoua N’Groma Nadège, Lorou Bi Maxime, Maneesh Phillip, Konan Anne Cécille, Rie Yotsu

**Affiliations:** 1 Croissance et Optimisation des Entreprises (COPTIMENT), Abidjan, Cote d’Ivoire,; 2 University of Felix Houphouët-Boigny, Abidjan, Côte d’Ivoire,; 3 Medical College of Georgia, Augusta, GeorgiaUnited States of America; 4 Institute of Ethno-sociology, University of Felix Houphouët-Boigny, Abidjan, Côte d’Ivoire,; 5 Cabinet d’Etudes Statistiques et Informatique, Abidjan, Cote d’Ivoire,; 6 Effect Hope Canada, Ontario, Canada; 7 Fédération des Associations des Handicapes de Cote d’Ivoire, Abidjan, Côte d’Ivoire,; 8 Department of Tropical Medicine and Infectious Disease, Tulane University, New Orleans, Louisiana, United States of America; 9 Department of Dermatology, National Center for Global Health and Medicine, Tokyo, Japan; Instituto Butantan, BRAZIL

## Abstract

**Introduction:**

Neglected tropical diseases (NTDs) are a priority in the public health agenda for Côte d’Ivoire, with persons living with disabilities due to NTDs (PD-NTDs) experiencing many challenges in their daily lives. Current policies do not sufficiently support PD-NTDs, thereby highlighting the need to identify opportunities for policy improvement.

**Methods:**

This study was carried out in two phases: first to identify the current needs (formative phase) and then to develop a pilot strategy (implementation phase). In the formative phase, pertinent current legislation was reviewed. Then, interviews of PD-NTDs and their caregivers were conducted and analyzed quantitatively. Thematic analysis of focus groups with key community stakeholders was also completed. In the implementation phase, a pilot strategy was developed to address the identified issues.

**Results:**

172 PD-NTDs and their caregivers were surveyed through this study. 99% of PD-NTDs expressed a need for rehabilitation, healthcare and psychosocial support. More than 80% endorsed the need for healthcare services and free medications, 92% expressed the need for educational services, and 83% reported economic difficulties. Furthermore, 30% of PD-NTDs reported limited awareness of specialized care services available in the community, 25% endorsed limited knowledge of current legislation that protects the rights of people living with disabilities, and 38% faced significant stigma and discrimination within the past 12 months. Fifty-six interviews with key informants further echoed these gaps. A pilot strategy was developed with four pillars to (1) increase community advocacy, (2) combat stigma, (3) promote mutual support among PD-NTDs, and (4) improve the sustainability of the effort. Forty-one out of 49 activities were completed.

**Discussion:**

This project represents a comprehensive effort to identify policy opportunities to effectively support PD-NTDs. This approach may be used by other organizations that plan to develop initiatives to target the needs of PD-NTDs in their local communities.

## Author summary

This project is a mixed-methods study and pilot strategy that aims to identify and address the needs of people living with disabilities due to Neglected Tropical Diseases (PD-NTDs) in Côte d’Ivoire. The project was completed in two stages: a formative phase to guide the development and the implementation of a pilot strategy. The initial findings revealed while there are some policies in Côte d’Ivoire to support people affected by disabilities, their needs were not sufficiently addressed, raising the opportunity for advocacy to target this gap. In particular, PD-NTDs needed greater health, financial, educational and social opportunities, for which the pilot strategy aimed to improve by empowering PD-NTDs. The project’s focus on encompassing multiple NTDs – as opposed to a single disease strategy – and involvement of PD-NTDs throughout the strategy contributed to the satisfactory fulfillment of the project’s objectives. We are hopeful that the results of this study will advance policy development in Côte d’Ivoire to protect the rights of PD-NTDs on a national level.

## Background

Neglected tropical diseases (NTDs) are a subset of diseases that are endemic to many tropical regions across the world, including Côte d’Ivoire. The National Plan for Health Development 2021–2025 states that Côte d’Ivoire is endemic for 10 NTDs: onchocerciasis, lymphatic filariasis (LF), soil-transmitted helminth (STH), trachoma, schistosomiasis, leprosy, Buruli ulcer, yaws, human African trypanosomiasis (HAT), and lastly Guinea worm, which has been eradicated [1]. Treatment of NTDs is a major public health priority for the country with ongoing mass drug-administration (MDA) programs for onchocerciasis, LF, STH, and schistosomiasis [1].

Unfortunately, people living with disabilities due to NTDs (PD-NTDs) often receive delayed diagnoses and treatment, leading to permanent disabilities. For example, even with established MDA programs for LF, PD-NTDs face multifactorial difficulties in receiving services from Morbidity Management and Disability Prevention (MMDP) programs, increasing their risk of disability [2]. Thus, it is difficult to estimate the true burden of NTD-related disability due to limited infrastructure and resources available for public health surveillance [3].

Disabilities have historically been linked to spiritual beliefs in Côte d’Ivoire, which contribute to significant stigma and exclusion within the community. Additionally, patients affected by NTDs face challenges such as unemployment, limited access to education, and sparse health resources. Although there are existing policies and regulations that aim to support PD-NTDs, there is little consideration of their self-endorsed needs in current public health initiatives.

The health system cannot solely manage the socio-economic consequences of PD-NTDs; these issues require a strong coordinated effort from several sectors of human development. Previous literature demonstrates that patients living with disabilities in Africa experience existing financial barriers and healthcare access [4–8]. The absence of inclusive employment opportunities, infrastructural barriers, and exclusive social environments at work impede people with disabilities in being able to pursue job opportunities [4,5]. Furthermore, the lack of socioeconomic support may exacerbate their difficulties in pursuing medical services, such as the absence of affordable transportation and inability to pay for healthcare [6]. Other notable barriers included insufficient support at healthcare centers, inadequate resources, and cultural stigma that amplify the challenges of patients affected by disabilities [7].

While there are a wide variety of studies that investigate the psychosocial and economic reintegration of people affected by disabilities [9,10], research regarding barriers specific to PD-NTDs is limited, which may be attributed to the difficulty of public health programs to reach these populations [11]. In a recent qualitative study, a focus group discussion with PD-NTDs revealed that community stigma – both about the disease and treatment – and low literacy were significant barriers in MDA programs in Côte d’Ivoire [12]. However, these findings are limited to one group; more extensive research is needed to highlight more perspectives of PD-NTDs to thoroughly investigate the current gap and outline the necessary interventions. Ensuring that people with disabilities caused by NTDs are not “left behind” will contribute to achieving the Sustainable Development Goal 3 and reduce the disease burden of NTDs [13].

Thus, the purpose of this study was to identify the current psychosocial, physical and economic difficulties that PD-NTDs face in their daily lives and create a strategy that would address these issues and inform policy opportunities. We use a mixed-methods approach to comprehensively evaluate the needs of PD-NTDs, including an analysis of the experiences of PD-NTDs and their caregivers about existing policies, regulations and support services. Through a patient-centered model, this strategy aims to improve national and global knowledge, increase the inclusion of PD-NTDs in public health programming, and end discrimination and stigma of persons affected by NTDs.

## Methods

### Ethical approval

This protocol was approved by Comité National d’Ethique des Sciences de la Vie et de la Santé for both formative and implementation phases. The research team was comprehensively trained on how to maintain confidentiality and privacy of participants prior to fieldwork. All participants provided informed consent (verbal or written) prior to their participation. All data was stored securely with access solely granted for trained team members. Information from quantitative and qualitative surveys were anonymized prior to analysis.

**Fig 1:**
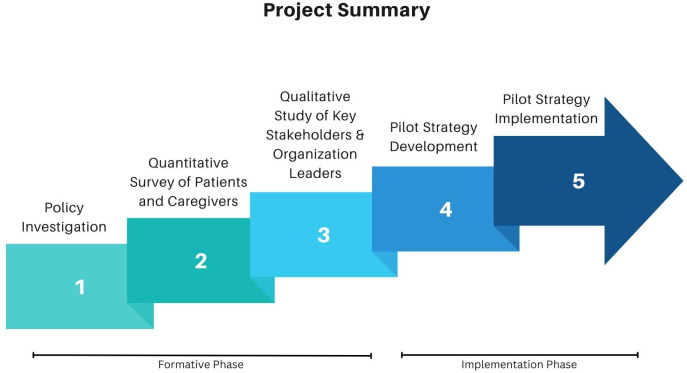
Project summary schematic.

This study was divided into two parts: the first being a formative phase to guide the creation of the strategy and the second being its implementation (Fig 1). In addition to the study team members, PD-NTDs were involved to assist with the proposal design and the execution of the formative and implementation phases.

### Formative phase

**Fig 2:**
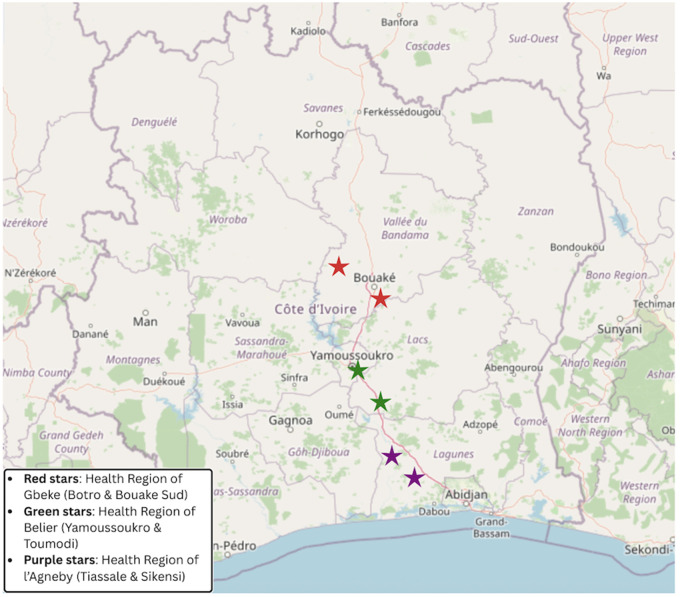
Selected Study Sites (two health districts were selected for each health region). Red represents the Gbeke Health Region (comprised health district of Bouake Sud and Botro). Green represents the Belier Health Region (comprised health districts of Yamoussoukro and Toumodi). Purple represents the Agneby Health Region (comprised health districts of Tiassalé and Sikensi). Map data is from OpenStreetMap (OpenStreetMap.org), which is available under the Open Database License.

For the formative phase, a cross-sectional design with a mixed-methods approach was developed. There were three primary steps, including a review of current legislation, survey of PD-NTDs and their caregivers, and interviews of key informants. Three health regions that are endemic to multiple NTDs and have established treatment programs were selected. Within each region, two health districts reporting the highest number of cases were selected (Fig 2). Specifically, health regions contain multiple health districts, each of which provide healthcare services to the communities within its area.

### Brief policy investigation

Legislative documents of Côte d’Ivoire, including the constitution, international conventions that were ratified by the country, and decrees, were extracted from the internet to identify the national regulations set in place to protect people affected by disabilities. The pertinent legislation was compiled to provide a greater understanding of the current policies.

### Quantitative study

The quantitative study consisted of exploring the needs, knowledge, attitudes and practices (KAP) of PD-NTDs and caregivers through interviewer-administered questionnaires (led by author Maxime). Using a 95% confidence level, 10% margin of error, and an assumed proportion of 0.5 (given that the true prevalence of disabilities amongst patients with NTDs is unknown), the minimum sample size needed was 96 individuals. For practical purposes, this estimate was rounded up to 100 individuals. We also aimed to recruit the same number of caregivers in a 1:1 ratio, leading to a target sample size of 100 PD-NTDs and 100 of their caregivers.

Using the health system’s records, PD-NTDs were identified across the six study areas. 100 patients were randomly selected from this list, comprising of the sampling frame. Inclusion criteria for PD-NTDs were those who lived in the target regions, were able to answer the interview questions, and consented to participate in the study. Patients affected by disabilities from NTDs included victims of amputation, retraction, deformity, leg edema, scrotal edema, and vision impairment. Exclusion criteria included patients with other conditions, individuals who did not reside in the target area, were not able to answer the interview questions or did not provide consent to participate.

In addition to the PD-NTDs, we also recruited their primary caregivers. Inclusion criteria for caregivers included individuals who was the primary caregiver of a patient affected by a NTD for at least 3 months, lived in the target region, were able to answer the interview questions, and provided their consent. Exclusion criteria included individuals who were not the primary caregiver, caregivers of patients who were not affected by a NTD, who did not live in the target area, were not able to answer interview questions, and did not provide their consent to participate.

Data collection was executed with the assistance of mobile technology, specifically with an open data kit app to assist with data collection. Information from these interviews were evaluated using quantitative data analysis techniques to assess the frequency of responses.

### Qualitative study

Questionnaires and interview guides were developed by the study team and shared with stakeholders to collect their perceptions on the needs and challenges faced by PD-NTDs (S1 File). These questionnaires and interview guides were then tested through a trial period with one community in the Tiassale Health District to identify any potential misunderstandings from interviewees and interviewers. The findings of this trial period allowed us to determine the amount of time needed for the interviews and make any necessary changes to the guides. After finalizing the guides for semi-structured interviews, focus group discussions, and in-depth interviews, interviews with key informants were conducted in person (by author Nadege). Interviews were conducted over a ten-day period in February 2022 with individual interviews lasting 45 minutes and focus groups discussions up to 60–90 minutes on average. Representatives from governmental offices, non-governmental organizations, health institutions, and specialized care centers were recruited. Inclusion criteria included employees who had worked at one of these institutions for at least 6 months and were involved with the implementation of the pilot program. All interviewees provided informed consent prior to the interview. Confidentiality was maintained by omitting any personal identifiers. All data was anonymized, securely stored on encrypted servers, and was only accessible to trained team members.

We also recruited focus groups of PD-NTDs and caregivers for each health region, with three focus groups for PD-NTDs and 3 focus groups for caregivers. In order to obtain a diverse range of perspectives, groups of PD-NTDs and caregivers were recruited independently and were not linked to each other. The inclusion criteria for the focus groups included anyone who consented to participate in the discussions; anyone who did not provide consent was excluded.

This section of the study was used to collect relevant information regarding existing policies and programs that currently exist to support patients on a national and regional level. This data was recorded using a voice recorder and transcribed using MAXQDA 2018 software for data analysis.

A quality assurance and control system were put in place, specifically with the use of mobile devices for data collection, validation checks from supervisors, and daily review of the acquired data. Both qualitative analysis (content and thematic analysis) and quantitative analysis (univariate and bivariate descriptive statistics and statistical significance testing using Stata version 14 software) were carried out.

### Implementation phase

To address the findings of the formative phase, a strategy was developed to mobilize and strengthen the capacity of PD-NTDs to advocate for themselves. The objective of the strategy was to increase the ability of PD-NTDs to influence public health initiatives to improve their access to physical, psychosocial, economic, and rehabilitation services in Côte d’Ivoire. The strategy was implemented in the health region of Gkeke by an organization that supports people affected by disabilities named Federation of Organizations of People with Handicap of Côte d’Ivoire (FAHCI) for 15 months. Through FAHCI, PD-NTDs were directly able to lead and participate throughout the entire project to ensure that the strategy continuously addressed their concerns.

## Results

### Formative phase

#### Existing laws and policies protecting people living with disabilities.

Seventeen legal documents were identified for establishing protective rights of people affected by disabilities, including the constitution, international conventions, laws and decrees of Côte d’Ivoire (S1 Table). The constitution of Côte d’Ivoire has specific clauses that protect people affected by disabilities and their access to resources, particularly in Articles 32 and 33. Article 32 guaranteed the provision of resources for people living with disabilities, including healthcare, education, employment, and leisure activities [14]. Article 33 targeted the discrimination that people with disabilities may face, specifically in receiving these resources [14]. While these clauses provided a general protection of rights for people living with disabilities, there were additional laws, decrees, and regulations to protect specific aspects of these rights. In particular, there were regulations within the Criminal Code to protect people living with disabilities who are “incapable of protecting themselves” from abandonment or isolation. Any violations would result in imprisonment and monetary penalties based on the severity of the situation [15]. For educational opportunities, an annual quota was established to provide a number of scholarships for people living with disabilities (Article 12, paragraph 2) to reduce financial barriers in pursuing education [16].

Employment was another area that was covered by these laws. For instance, Article 12 of Law 2015–532 established a quota for employers to hire people affected by disabilities who had the professional qualifications [17]. Furthermore, the government committed to provide additional support in covering costs of treatments, use of equipment including prosthetic equipment, and transportation for people living with disabilities through the “National Social Security Fund of Côte d’Ivoire (CNPS)” [18]. However, the actual efficacy of these laws was questionable, specifically in regards to the ability of patients affected by disabilities to obtain these resources.

### Quantitative study results

A simple random sample of 172 people (103 people with disabilities due to NTDs and 69 caregivers) was selected for the survey with 16 PD-NTDs per research site on average. Of the surveyed PD-NTDs, 58 were male and 45 were female. Ages of PD-NTDs ranged from 20 to 60 years old. Forty-five individuals were single and 39 were married or living with another individual. As for caregivers, 30 individuals were male and 39 individuals were female. Fifty of surveyed caregivers were married or living with another individual. The most common diseases among PD-NTDs were Buruli ulcers (57.3%), followed by leprosy (21.4%), lymphatic filariasis (18.4%) and onchocerciasis (2.9%). Most of the surveyed individuals had received primary education; only 2% of PD-NTDs and 6% of caregivers were able to achieve a higher level of education. More than 60% of the surveyed PD-NTDs were unemployed. Eleven percent of PD-NTDs have full time jobs while 22% had part-time occupations. On the other hand, the majority of caregivers (67%) were employed. As we recruited the caregivers who cared for the PD-NTDs that were selected for our study, there was a discrepancy between the number of patients and number of caregivers. Specifically, there were some PD-NTDs who did not have a primary caregiver or had a caregiver who did not meet the inclusion criteria.

The needs expressed by PD-NTDs were analyzed within the following four domains:

(i) *Healthcare, physical rehabilitation and psychological support*

In the survey, 99% of PD-NTDs expressed a need for rehabilitation, healthcare, and psychosocial support (Table 1). Furthermore, regular medical care and donations of medications were needed by PD-NTDs affected by leprosy (81.8%), Buruli ulcer (84.7%), lymphatic filariasis (89.5%) and onchocerciasis (66.7%). Many PD-NTDs did not endorse the needs of rehabilitative devices, as they did not realize that these devices could be useful to them.

**Table 1:** Identified healthcare needs by PD-NTDs and caregivers, by disease subtype.

Healthcare Needs	PD-NTDs				Caregivers			
	Leprosy (n = 22)	Buruli Ulcer (n = 59)	Lymphatic Filariasis (n = 19)	Oncho-cerciasis (n = 3)	Leprosy (n = 14)	Buruli Ulcer (n = 39)	Lymphatic Filariasis (n = 14)	Oncho-cerciasis (n = 2)
Free medical care and medication donations	18 (81.8%)	50 (84.7%)	17 (89.5%)	2 (66.7%)	12 (85.7%)	32 (82.1%)	14 (100.0%)	1 (50.0%)
Free medical care and accessible medication donations	13 (72.7%)	31 (52.5%)	11 (57.9%)	2 (66.7%)	7 (50.0%)	23 (59.0%)	6 (42.9%)	1 (50.0%)
Rehabilitation Devices	3 (13.6%)	8 (13.6%)	0 (0.0%)	0 (0.0%)	1 (7.1%)	9 (23.1%)	2 (14.3%)	0 (0.0%)
Accessibility of health structures	0 (0.0%)	1 (1.7%)	0 (0.0%)	0 (0.0%)	0 (0.0%)	2 (5.1%)	2 (14.3%)	0 (0.0%)
Reception by staff and consideration of specific constraints	2 (9.1%)	1 (1.7%)	1 (5.3%)	0 (0.0%)	1 (7.1%)	0 (0.0%)	0 (0.0%)	0 (0.0%)
Psychological support	1 (4.5%)	5 (8.5%)	2 (10.5%)	0 (0.0%)	0 (0.0%)	4 (10.3%)	2 (14.3%)	1 (100.0%)
Spiritual support	0 (0.0%)	3 (5.1%)	1 (5.3%)	0 (0.0%)	1 (7.1%)	4 (10.3%)	1 (7.1%)	0 (0.0%)
Other health needs^*^	2 (9.1%)	1 (1.7%)	0 (0.0%)	1 (33.3%)	2 (14.3%)	1 (2.6%)	2 (14.3%)	1 (50.0%)

* Other health needs were additional medical issues that were reported by participants but were not directly related to their NTD. These included other comorbidities, including chronic pain and recurrent skin infections.

PD-NTDs also reported difficulties that arose from existing prejudices about disability and NTDs, stigmatization, and social discrimination within the community. Approximately 20% “felt different from other members in society” and endorsed behaviors of self-stigmatization, while 38% have directly experienced stigma and discrimination during the last 12 months. This further played into the care that PD-NTDs received, as a few PD-NTDs (4) and caregivers (1) reported that they experienced discrimination and breaches in confidentiality when receiving care through the healthcare system.

(ii) *Information related to the rights of persons living with disabilities due to NTDs*

Overall, more than 75% of PD-NTDs have a limited awareness regarding the existing legislation enacted to protect people affected by disabilities. Thirty percent of PD-NTDs lacked knowledge about specialized care structures and rehabilitation services available in the community. Caregivers similarly shared a limited understanding of existing regulations and specialized structures (Table 2). Furthermore, fewer than 10% of surveyed PD-NTDs had knowledge about the causes and modes of transmission of the NTDs that they were affected by, thereby exhibiting limited health literacy. A greater proportion of PD-NTDs and their caregivers recognized symptoms and treatments of NTDs (Table 2).

**Table 2:** Understanding of NTDs among surveyed PD-NTDs and caregivers.

	PD-NTDs (n = 103)	Caregivers (n = 69)
Knowledge of Existing Regulations to Protect PD-NTDs	20 (19.4%)	16 (23.2%)
Knowledge of Specialized Structures to Assist PD-NTDs	72 (70%)	51 (73.9%)
Knowledge of the Cause of their Afflicting NTD	6 (5.8%)	4 (5.8%)
Knowledge of Symptoms of NTDs^**^	75 (72.8%)	60 (87.0%)
Knowledge of Transmission of NTDs^**^	10 (9.7%)	7 (10.1%)
Knowledge of Treatments for NTDs^**^	31 (30.1%)	29 (42.0%)

** NTDs: Neglected Tropical Diseases (Leprosy, Buruli Ulcer, Yaws, Lymphatic Filariasis, Onchocerciasis)

(iii) *Educational opportunities*

Education was a primary need for people with disabilities affected by neglected tropical diseases with 92% of surveyed individuals expressing the need for educational services (Table 3). In particular, participants reported a need for free school registration, access to school supplies, and access to school canteens.

**Table 3:** Identified educational needs by PD-NTDs and caregivers by disease subtype.

Educational needs	PD-NTDs				Caregivers			
	Leprosy (n = 22)	Buruli Ulcer (n = 59)	Lymphatic Filariasis (n = 19)	Oncho-cerciasis (n = 3)	Leprosy (n = 14)	Buruli Ulcer (n = 39)	Lymphatic Filariasis (n = 14)	Oncho-cerciasis (n = 2)
Free registration	21 (95.5%)	50 (84.7%)	15 (78.9%)	2 (66.7%)	13 (92.9%)	33 (84.6%)	12 (85.7%)	2 (100.0%)
Free school supplies	19 (86.4%)	46 (78.0%)	14 (73.7%)	2 (66.7%)	13 (92.9%)	35 (89.7%)	13 (92.9%)	1 (50.0%)
School canteens	3 (13.6%)	16 (27.1%)	3 (15.8%)	0 (0.0%)	3 (21.4%)	8 (20.5%)	2 (14.3%)	1 (50.0%)
Access to specialized educational institutions^*^	3 (13.6%)	5 (8.5%)	3 (15.8%)	1 (33.3%)	3 (21.4%)	5 (12.8%)	2 (14.3%)	0 (0.0%)
Free School Support	2 (9.1%)	1 (1.7%)	2 (10.5%)	0 (0.0%)	1 (7.1%)	3 (7.7%)	0 (0.0%)	0 (0.0%)
Other Needs	1 (4.5%)	4 (6.8%)	1 (5.3%)	1 (33.3%)	0 (0.0%)	0 (0.0%)	0 (0.0%)	0 (0.0%)

* Specialized educational institutions may provide additional support for people with disabilities, such as an institution dedicated to individuals with vision impairment

(iv) *Socio-professional integration and support for self-employment.*

Financial difficulties were frequently reported by participants; 99% of surveyed PD-NTDs identified a need for socioeconomic support. Nearly 79% of PD-NTDs earned monthly incomes less than 50,000 West African Francs (FCFA) per month, amounting to 83 US dollars monthly or less than $3 per day. However, there was a profound financial disparity between female and male PD-NTDs with 94.4% of female PD-NTDs reporting monthly incomes less than 50,000 FCFAs, compared to 65.0% of male PD-NTDs. Moreover, 83% of PD-NTDs reported the need for financial resources to generate additional income (Table 4). These financial difficulties were closely tied to the need for more employment opportunities, as 67% of PD-NTDs did not have a job, 22% had part-time occupations, and 11% had a full-time job in the informal sector.

**Table 4:** Identified socioeconomic needs by PD-NTDs and caregivers by disease subtype.

Socioeconomic Needs	PD-NTDs				Caregivers			
	Leprosy (n = 22)	Buruli Ulcer (n = 59)	Lymphatic Filariasis (n = 19)	Onchocerciasis (n = 3)	Leprosy (n = 14)	Buruli Ulcer (n = 39)	Lymphatic Filariasis (n = 14)	Onchocerciasis (n = 2)
Obtain a stable, well-paid job	5 (22.7%)	16 (27.1%)	3 (15.8%)	0 (0.0%)	4 (28.6%)	10 (25.6%)	4 (28.6%)	0 (0.0%)
Obtaining funding for income-generating activities	16 (72.7%)	47 (79.7%)	18 (94.7%)	2 (66.7%)	11 (78.6%)	33 (84.6%)	14 (100.0%)	2 (100.0%)
Take into account the needs specific to PD-NTDs	5 (22.7%)	7 (11.9%)	2 (10.5%)	0 (0.0%)	3 (21.4%)	7 (17.9%)	2 (14.3%)	0 (0.0%)
Other socioeconomic needs	1 (4.5%)	5 (8.5%)	0 (0.0%)	1 (33.3%)	1 (7.1%)	0 (0.0%)	0 (0.0%)	0 (0.0%)

### Qualitative study results

Various stakeholders from community organizations, health centers and governmental offices were interviewed to better understand current support given to PD-NTDs and the knowledge surrounding the laws and regulations that protect them in Côte d’Ivoire (S2 Table).

A total of 56 individuals were interviewed, including 19 key informants from selected institutions, 12 caregivers and 3 focus groups each with 8 PD-NTDs.

Common themes were identified with selected quotes from the key informants (Table 5).

**Table 5:** Themes from key informant interviews with quotes.

Theme	Selected Quotes
**Healthcare Needs**	
Limited Access to Healthcare Services & Training	- “Here, there are no care structures at the level of Buruli Ulcer….understand that it is not easy for these people to travel. For example, we were trained for everything just to prevent what we call disabilities during treatment but when the reactions during treatment of leprosy occur, we do not have the commodities to manage these cases” (Interview with the NTD focal person). - “In terms of health care, it turns out that they are left to their own overall. Well, here in Toumodi, our patients are mostly at home, but those who manage to come to the center, well, when they come, we do what we can to help them, well, we do everything we can at our level to help them. Those who don’t have the means, we manage to pay for medication for some, but it’s not all of them, so that’s a bit like it. To benefit from care, when I have a bit of means, I can buy medication for some, otherwise, generally speaking, they must take care of themselves.” (Interview with contact at public health center)
**Education**	
Insufficient Support for Parents of PD-NTDs	- “If a parent has a disabled child who goes to school, I think that the system has not planned anything special for these sick children. It is the parent who takes care of his child. He takes him to school. He sees how he can help him so that he can continue his schooling so that he does not lose out so that his future is not compromised; that is the responsibility of the parents. I do not think that there is a social institution in charge of assisting these parents. If it exists, it is not known.” (Interview with the governmental program) - “If I do not have the means to take care of myself, can I educate my child? I cannot send him to school, there is no way for me to find food…it is always the problem of poverty.” (Interview with the NTD focal point)
Academic Difficulties faced by Students	- “There are some pupils who are handicapped by Buruli ulcer. Some have dropped out of school because they could not combine the two (disease management and school); well, the hospitalization lasts for 2 months on average…. They lost several years before continuing, [while] there are some who no longer continued school” (Interview with the focal point) - “So at our level, there is this mechanism that is in place to keep children at a school level, now when they leave, there are some who are discouraged, they cannot reintegrate” (Interview with the representative of the public health institution).
Difficulties of Social Integration for Affected Students	- “I met a child during a campaign, a child with Buruli ulcer disabilities. This child stopped going to school because his disability was in his lower limbs, he does not see himself as the others. So he stopped going to school” (Interview with representative at health center) - “It is the school that puts in place implicit or explicit mechanisms to reject them or even it is the families who spontaneously say well [their] child does not belong. If he goes [to school], they will make fun of him and therefore it is not worth his while to go.” (Interview with the representative of the health center)
**Financial Challenges**	
Financial Burdens	- “The problems are generally…on the financial level; either [PD-NTDs] are single people or they are more or less single people or at least in a large family…and there is no financial support” (Interview with a nurse).
Lack of Employment Opportunities	- “It is complicated because here all we have as work for these people is agricultural companies. Someone who has a disability …will not be hired in an agricultural company” (Interview with the focal NTD person)
Impact of Stigma on Financial Wellbeing	- “The most serious problem is at the level of stigmatization. They are rejected by their families, their communities and then themselves first, people are ashamed of those who have hydrocele…there is also this stigmatization aspect and very often it results in a cessation of the activity that they were carrying out, so the rest follows, the economic dependency aspect since they can no longer carry out their activities, they will often depend on other people” (Interview with the health department representative)
**Social Difficulties**	
Social Isolation	- “They have a problem of social rehabilitation. In fact, they are people who are first marginalized in a village especially when it is the case of leprosy. They become a burden for the society because they have difficulty running their usual activities, which means that psychologically, they really need help and assistance. The population must understand that they are not outcasts and people who should be pushed aside. Instead, we must try to get closer to these people so that they feel alive. Usually, they are pushed aside in a small corner... Well, this is the kind of society in which we lived here” (Interview with the NTD focal person) - “Overall, they are left to their own, there are some who have even been abandoned by their wives, they are forced to cut wood to sell just to have some little money to buy food, there are some who do not have hands to make fire, they depend on others, there are few patients in this condition” (Interview with NTD focal person)
Lack of Psychological Support	- “There is no institution created to provide psychological support services. there is not a psychologist really within the health facility, so we do what we can do to try to support the patients... [there] is a religious environment they also try to sympathize, that’s all we do for the moment there is no psychologist, you know” (Interview with the representative of health center). - “At my level there is no care as such, well, I do not think so; there is no psychological support” (NTD focal person) - “To my knowledge at the district level there is no psychosocial or economic support service” (Interview NTD Focal Person)
Exclusion from Community Organizations that support patients with disabilities	- “They do not have an association and then also normally they must be part of what is called... they are people with disabilities, or they are organizations of people with disabilities. But unfortunately, even the organizations that are structured for people with disabilities do not consider them as people with disabilities, so there is a form of exclusion even within that” (Interview with a representative of mental health center)
Knowledge surrounding Legislation of Cote d’Ivoire	- “At my level, I am not aware of any law or legislative texts that are related to disability. I am not aware of any initiative to make a law at this level and being a specialist in the field I have never been invited to participate, no I don’t know. If there are laws, I am not aware of these laws!” (Interview with the public health institution representative) - “I hear about the laws that protect them, but I don’t know their content” (Focus group with caregivers)

More information about the project is available in S1 Data and S2 Data.

## Implementation phase

### Pilot strategy

The results of the formative phase revealed that there was a limited capacity for advocacy and empowerment for organizations that support PD-NTDs, in addition to limited involvement of PD-NTDs within these programs. Thus, the pilot strategy aimed to address these gaps with four pillars, including (1) increase advocacy and awareness for improved access to physical, psychosocial, educational, and economic rehabilitation services (2) fight against stigma and promote the rights of people living with disabilities, (3) increase mutual support amongst people living with disabilities, and (4) strengthen the organizational capacities of the overseeing organization, FAHCI, to sustain the pilot strategy’s actions.

In particular, the following activities were completed in Gbeke as a part of the pilot strategy:

**Box 1: Activities completed in the Pilot Strategy**

- The recruitment of a project officer at FAHCI and a team of volunteers among people living with disabilities for the implementation of the strategy.
- The development of an operating plan, including monitoring and evaluation, to mobilize PD-NTDs in influencing policies and programs to improve access to socio-economic rehabilitation services.
- The launch of the implementation phase of the project.
- The purchase of equipment for the project’s implementation team.
- The signing of engagement letters with the project’s key stakeholders and organizations to include assistance to PD-NTDs in their missions and objectives.
- The organization of weekly coordination meetings and coaching of the project team, including a project officer from FAHCI.
- The development of a toolkit for the scaling up of the strategy.

A total of 41 activities were fully implemented out of 49 activities, resulting in a completion rate of 83.7% (S3 Table). Only three activities were partially completed (6.1%) and five activities could not be finished due to time and funding constraints (10.2%).

For the first pillar of the strategy, focused on advocacy and raising awareness for improved access to physical, psychosocial, educational and economic rehabilitation services, 26 out of 29 activities were fully completed (with a completion rate of 89.7%). FAHCI established fifteen new partnerships with 11 institutions and 4 health districts (Bouake North, Beoumi, Sakassou and Bouake North-Est). These partnerships were created to provide sustainable health, socioeconomic and physical rehabilitation services for PD-NTDs (Table 6).

**Table 6:** List of institutions engaged by the project for the provision of rehabilitation services for PD-NTDs.

#	Institutions	Type of Institution	Partnership and Ongoing Actions
01	Youth Employment Agency, Bouake	- Government Agency	- Technical support for entrepreneurship - Funding of project for PD-NTDs
02	Regional Directorate of Social Protection, Bouake	- Government Agency	- Exemption of UHC premium for PD-NTDs
03	CARITAS Bouake	- NGO	- Free Access to Health Care
04	Municipality of Bouake	- Community Based Organization	- Integration of PD-NTDs needs in the social support provided by the municipality of Bouake
05	Regional Directorate of the Ministry of Education and Literacy, Bouake	- Government Agency	- Support access to school for students with disabilities due to neglected tropical diseases - Free school kits for students with disabilities due to neglected tropical diseases
06	Ivorian Radio and Television Broadcasting (RTI-Bouake)	- Government Agency	- Broadcasting of sensitization message of NTDs - Media coverage of events organized by PD-NTDs
07	COOPEC Bouake (microcredit organization)	- Private Company	- Technical support for entrepreneurship - Funding of project for PD-NTDs
08	Regional Directorate of Employment Inspection	- Government Agency	- Provision of policies, regulation and laws document informing on the right of DP - Provision of a resource person for sensitization of rights of people living with disabilities in employment
09	Jean-Vatelot de Bouaké Center	- Health center	- Provision of healthcare and physical rehabilitation to people living with disabilities
10	Legal Clinic Directorate Bouake	- NGO	- Improvement and access to the rights of PD-NTDs - Equitable access to justice for PD-NTDs - Collaboration with the Dignity project team
11	Court of First Instance of Bouake	- Government Agency	- Legal protection of PD-NTDs - Enforcement of the laws protecting people living with disabilities

To address stigmatization and promoting respect for the rights of people with disabilities, 7 out of 9 activities were completed (77.8% completion rate). New working relationships with the Ministry of Justice and other non-governmental organizations were created to defend the rights of people affected by disabilities. Through local radio and television networks, a campaign to raise community awareness was also executed. The immediate effect of these programs was demonstrated by a strengthened capacity of the project’s implementation team members to conduct advocacy activities and to empower members of associations supporting people with disabilities.

To increase community mutual support among PD-NTDs, 3 out of 5 planned activities were fully completed, with one additional activity partially completed, resulting in a completion rate between 60–80%. Most significantly, there was a development of a census system to register PD-NTDs and their needs. This system was regularly used to enroll 157 patients throughout the initial stages of the strategy and is still being used to identify people living with disabilities and their needs.

Finally, the organizational capacity of the overseeing organization, FAHCI, was prioritized. Five out of six activities were fully implemented, leading to a completion rate of 83.3% (S3 Table). Specifically, these activities focused on communication with team members and stakeholders, project development and evaluation, in addition to providing technology support to increase the ability of FAHCI to advocate for PD-NTDs.

## Discussion

Building upon a qualitative study that explored the perspectives from one PD-NTD group in Côte d’Ivoire [12], the findings of our mixed-methods study have elucidated the multifactorial difficulties faced by PD-NTDs, highlighting urgent unmet needs. Issues of unemployment and challenges in education are deeply affected by the community’s stigma. The subsequent limitations in financial autonomy further hinder the ability of PD-NTDs to receive the healthcare services they need. These concerns are exacerbated by the lack of both informal and formal organizational support, with the exclusion of PD-NTDs from formal associations dedicated to supporting people affected by disabilities. Moreover, there are currently no organizations that foster a community for PD-NTDs to support one another, resulting in self-isolation and internalization of the community’s stigmas. Key stakeholders in the community also echoed the needs of PD-NTDs, which are consistent with findings from other studies in Brazil and India, where stakeholders identified that social stigma and insufficient resources can negatively impact the lives of PD-NTDs [19]. Despite the different cultural context, the lack of access to psychosocial, healthcare, and financial resources is a pervasive issue, indicating a need for multifactorial approaches to support PD-NTDs.

While there are regulations that offer protection of PD-NTDs in Côte d’Ivoire, persistent implementation gaps limit access to resources. For instance, although Article 12 of Law 2015–532 provided a quota for employment of PD-NTDs, it may be difficult for PD-NTDs to receive the necessary training to qualify for these job opportunities. Thus, it is challenging to assess the actual utilization of these rights as outlined in the country’s legislation. New policies can be developed to address these gaps, for instance, to provide opportunities that offer specialized training to expand job prospects for PD-NTDs. Policies that support socio-professional integration and self-employment opportunities could also help to provide more economic autonomy. However, it is important to note that advocacy efforts should not only focus on developing new policies, but also on enforcing existing frameworks to enhance support for PD-NTDs. As expressed by study participants, there was limited community awareness about regulations that protect the rights of PD-NTDs in Côte d’Ivoire. Thus, campaigns that raise awareness about protective regulations in the community can be effective in addressing this knowledge gap. These initiatives must further focus on eliminating barriers that PD-NTDs may face in utilizing these mandated rights, such as limited literacy.^11^

In addition to legislation on a larger scale, local community-driven interventions have been found to be effective in addressing the needs of PD-NTDs [20–22]. After implementing their intervention, a community-based initiative in Nigeria developed three primary priorities for future improvement, including connecting patients to resources, establishing partnerships with local organizations and advocating for greater protection [22]. Our project had similar priorities, as we aimed to improve our target population’s health literacy and knowledge regarding NTDs, empower them to act, and foster community awareness to bridge access to resources that would ultimately lead to successful policy implementation. By evaluating the reported difficulties of PD-NTDs, their caregivers and key stakeholders, we were able to integrate these issues into the pilot strategy. Furthermore, we provided opportunities for local organizations that support PD-NTDs to expand upon their current efforts by involving local and national leaders. The development of these community partnerships was essential in guiding the sustainability of our efforts to control NTD-related complications in Côte d’Ivoire [19]. However, the actual delivery of these services, such as free medical care or improved access to services, is not the responsibility of the pilot project and will be carried out by the partnerships established in this project between the partner institutions and advocacy efforts from PD-NTDs. Thus, the long-term effects of these partnerships will be more evident when resources are effectively mobilized by the associations and made available to their members.

By building these partnerships with local organizations, we hope to have strengthened the available resources for PD-NTDs to target several structural barriers that PD-NTDs may face in these communities. Various activities were also completed to improve the overarching organization’s (FAHCI) capacity to advocate for and address these issues through their existing programming. Through FAHCI, PD-NTDs were involved throughout the design, implementation and evaluation of the project, verifying that we created practical objectives that effectively addressed their concerns. This strong coordinated effort ensured that PD-NTDs led the implementation of the strategy and guaranteed the completion of the four pillars of the person-centered strategy at an acceptable rate. Furthermore, representatives of FAHCI endorse that they were encouraged by the positive impact of the pilot strategy and plan to expand the programs to other areas within Côte d’Ivoire, which will help to sustain these efforts.

In a greater context, a recent change was initiated by the World Health Organization (WHO) in 2022 to develop a more inclusive framework for skin NTD management, specifically in targeting the most common skin NTDs that have the greatest disease burden in endemic areas [22,23]. This holistic approach is particularly important given that many skin NTDs require individual care and treatment, which can be resource intensive and thus, be difficult to control [24]. Integrated screening has been found to be successful in the detection and management of skin NTDs in Côte d’Ivoire, highlighting the merit of this approach [25–27]. Since cutaneous manifestations are primary clinical signs of the skin NTDs, there are many opportunities for comprehensive management of NTDs with the training of local professionals, tele-dermatology, and community-based interventions [28].

Thus, our project’s inclusive approach in addressing multiple NTDs minimizes the impacts of two primary limitations of the project, time and budget, while optimizing the effects of our pilot strategy. However, the impacts of these limitations were still evident as we were not able to complete all of our planned activities; only 41 activities out of 49 were able to be fully implemented due to these restrictions. Initially, the project was scheduled to be completed within 24 months, including 12 months for the formative phase and 12 months for the pilot strategy. The goal of this relatively short implementation period was to encourage PD-NTDs to be able to advocate for themselves with the support of key stakeholders in the community, rather than directly provide those resources (such as increasing access to healthcare). While the challenge of insufficient funding was partially addressed by the recruitment of volunteers among PD-NTDs, economic constraints significantly restricted our ability to engage with more institutions and complete more activities. Thus, these restrictions may limit the extent of health policy engagement and may further threaten the sustainability of the project to provide the resources needed by PD-NTDs.

We also recognize the impacts of the under-recruitment of the caregivers of PD-NTDs. As we recruited caregivers who took care of PD-NTDs who were included in our study, there were several PD-NTDs who did not have a caregiver. This limitation, however, supports a finding of our study that PD-NTDs may struggle to have sufficient social support.

As this is a pilot study, we believe that the results are promising in outlining legislative opportunities that effectively address concerns shared by PD-NTDs, caregivers, and key stakeholders. In the future, we hope that this project can continue to receive support with more resources over a longer period of time to assess the strategy’s long-term benefits on the daily lives of the PD-NTDs. The longer study period could also outline potential opportunities to build upon the current project design to assist more community members. However, to strengthen community-based interventions, policy initiatives are crucial in supplementing the efficacy of these efforts.

We hope that our strategy’s design is helpful to our colleagues who are interested in similar initiatives to empower PD-NTDs in their own communities. By maintaining a PD-NTD centered approach, colleagues from other countries can recognize the unique needs of PD-NTDs and policy changes in their communities. We have developed a toolkit that outlines our strategy and encompasses the lessons we learned may be used by interested colleagues (S4 Table).

To conclude, through this mixed-methods approach, the specific needs of PD-NTDs in Côte d’Ivoire and the limitations of the country’s current policies were identified. The involvement of PD-NTDs throughout the development and implementation process abide to the project’s overarching goal to create an effective pilot strategy. Furthermore, the establishment of an inclusive strategy to include patients affected by any neglected tropical disease helped to improve advocacy efforts. Therefore, the findings of our study demonstrate the merit of adopting a person-centered approach to identify the needs and develop an effective community-based intervention in Côte d’Ivoire.

## Supporting information

S1 Table Legislation reviewed in the manuscript. (DOCX)

S2 Table Information about the interviewed representatives. (DOCX)

S3 Table Information about the pilot strategy. (DOCX)

S4 Table Copy of the toolkit. (DOCX)

S1 Data Study report mid-term evaluation. (DOCX)

S2 Data Final Evaluation Study Report of the Dignity Pilot Project. (DOCX)

S1 File Interview guides used in this project. (ZIP)
