# Supplementary material for: People-centered strategies to mobilize people living with disabilities due to Neglected Tropical Diseases (PD-NTDs) to influence policy and programs: A mixed-methods study in Côte d’Ivoire
Source: PLoS Negl Trop Dis. 2025 Sep 8;19(9):e0013485. doi: 10.1371/journal.pntd.0013485 (PMC12431663; doi:10.1371/journal.pntd.0013485)
Supplement: S1 Table — (DOCX) [file pntd.0013485.s001.docx]

**S1 Table: List of Legislation that was Reviewed in Initial Literature Search**

| **Type of Legislation** | **Specific Laws (if applicable)** |
| --- | --- |
| Constitution of Côte d’Ivoire of 11/8/2016 |  |
| International Conventions Ratified by Côte d’Ivoire | 1. The International Convention on the Child Rights 2. Convention No. 159 concerning Vocational Rehabilitation and Employment of Disabled Persons 3. United Nations Convention on the Rights of Persons with Disabilities of 13 December 2006 |
| Laws of Côte d’Ivoire | 1. Law No. 98-594 of November 10, 1998, on Guidance for Persons with Disabilities  2. The General Tax Code  3. Law No. 99-477 of August 2, 1999, on the Social Security Code  4. The Code of Criminal Procedure  5. Law No. 2015-532 of July 20, 2015, on the Labor Code  6. The Electoral Code  7. The Interprofessional Collective Agreement of July 19, 1977  8. Law No. 2019-576 of June 26, 2019 establishing the Construction and Housing Code. |
| Decrees in Côte d’Ivoire | 1. Decree No. 96-615 of August 9, 1996 regulating scholarships for higher education in Côte d'Ivoire  2. Decree No. 2018-456 of May 9, 2018 relating to the employment of people with disabilities in the private sector.  3. Decree No. 2021-539 of September 22, 2021, establishing the creation, allocation, organization, and operation of the Technical Commission for Professional Orientation and Reclassification (COTOREP) in the public sector  4. Decree No. 2021-540 of September 22, 2021, establishing the creation, allocation, organization, and operation of the Technical Commission for Professional Orientation and Reclassification (COTOREP) in the private sector  5. Decree No. 2023-88 of February 15, 2023, establishing the creation, allocation, organization, and operation of the Fund for the Professional Integration of Persons with Disabilities |
