# Supplementary material for: People-centered strategies to mobilize people living with disabilities due to Neglected Tropical Diseases (PD-NTDs) to influence policy and programs: A mixed-methods study in Côte d’Ivoire
Source: PLoS Negl Trop Dis. 2025 Sep 8;19(9):e0013485. doi: 10.1371/journal.pntd.0013485 (PMC12431663; doi:10.1371/journal.pntd.0013485)
Supplement: S2 Table — (DOCX) [file pntd.0013485.s002.docx]

**S2 Table: Interviewed Representatives and their Respective Organizations**

| **Affiliated Organizations with Interviewees** | **Number of People Surveyed from Each Organization** |
| --- | --- |
| National Leprosy Elimination Program (PNEL) | 1 |
| National Program to Combat Buruli Ulcer (PNLUB) | 1 |
| National Program for the Fight against Neglected Tropical Diseases with Preventive Chemotherapy (PNLMTN-CP) | 1 |
| Federation of Organizations of People with Handicapp in Côte d'Ivoire (*FAHCI*) | 2 |
| Ministry of national education | 1 |
| Ministry responsible for employment and social protection | 1 |
| Ministry of Justice | 1 |
| Institutions responsible for mental health (Mental Health Program, Bouaké Psychiatric Institute) | 2 |
| Health regions | 3 |
| NTD focal points of health districts | 6 |
| Specialized care centers [*Tiassalé (Taabo), Yamoussoukro (Kongouanou), Bouaké (JB Vatelot)* | 3 |
| Focus Group with people with disabilities due to NTDs (1 per region) | 3 |
| Focus Group with caregivers of PD-NTDs (1 per region) | 3 |
| **Total** | **28** |
