# Supplementary material for: People-centered strategies to mobilize people living with disabilities due to Neglected Tropical Diseases (PD-NTDs) to influence policy and programs: A mixed-methods study in Côte d’Ivoire
Source: PLoS Negl Trop Dis. 2025 Sep 8;19(9):e0013485. doi: 10.1371/journal.pntd.0013485 (PMC12431663; doi:10.1371/journal.pntd.0013485)
Supplement: S3 Table — (DOCX) [file pntd.0013485.s003.docx]

**S3 Table: All planned activities and level of implementation**

Axe 1: Raising awareness and improving community advocacy

| **Commitment of Administrative Authorities & Community Leaders for the Strategy Implementation** | |
| --- | --- |
| Activity | Level of Implementation |
| Organize a project launch ceremony to make it known to the administrative authorities and community and association leaders of Bouaké | 100% |
| Obtaining stakeholder commitment to improving the quality of life of PH-MTN | 100% |
| Organize at least one meeting with each institution to strengthen collaboration | 100% |
| **Advocacy for Free Care, including Rehabilitation and Re-adaptation Care for PD-NTDs** | |
| Activity | Level of Implementation |
| Submit a request to the Ministry of Health and the Ministry of Social Protection to obtain free coverage for rehabilitation and re-adaptation care for PD-NTDs | 100% |
| Follow up on correspondence through at least one meeting with the authorities of the target institutions (Health and social protection). | 100% |
| **Strengthening Psychological Care for PD-NTDs** | |
| Activity | Level of Implementation |
| Organize an advocacy meeting targeting health district authorities so that psychological support is integrated into the care of PD-NTDs | 100% |
| Follow up with the PNEL for the training of health workers for this purpose | 100% |
| Participate in planning meetings on NTD control in health districts and at central level | 100% |
| **Improve the Knowledge and attitudes of PD-NTDs and their caregivers on laws and regulations protecting PD-NTDs** | |
| Activity | Level of Implementation |
| Organize a quarterly radio program to raise awareness about NTDs, recognition of suspicious signs, possible complications and management, as well as laws and regulations that protect people with disabilities | 100% |
| Share videos on WhatsApp and Facebook on awareness of NTDs, recognition of suspicious signs, possible complications and management, as well as laws and regulations that protect people with disabilities | 50% |
| Organize advocacy meetings with community leaders to remove cultural barriers and/or improve collaboration with the health system for the management of PD-NTDs | 100% |
| Participate in planning meetings on NTD control in health districts | 100% |
| **Mobilizing Resources for the Integration of Children with disabilities due to NTDs into the Education System through Advocacy and School Support** | |
| Activity | Level of Implementation |
| Identify children with disabilities due to NTDs with schooling needs during the PD-NTD census with the help of focal points | 100% |
| Organize an advocacy meeting with national education officials for the admission of children in need into schools | 100% |
| Organize a meeting to mobilize financial resources with financial partners and government aid agencies to obtain support for PD-NTDs | 100% |
| **The Promotion of Structures Specialized in Education of Public Health towards PD-NTDs** | |
| Activity | Level of Implementation |
| Identify existing specialized structures in Ivory Coast | 100% |
| Send awareness-raising letters and requests for admission of PD-NTDs to the identified structures. | 100% |
| Organize awareness-raising among PD-NTDs of school age or in training on the existence of specialized education structures and the conditions of access to them | 100% |
| Have a PD-NTD WhatsApp address book for sharing information | 100% |
| **Improving Access to Training for Adults disabled by NTDs** | |
| Activity | Level of Implementation |
| Produce and distribute leaflets (in digital and physical form) to PD-NTDs providing information on literacy and vocational training opportunities. | 100% |
| Organize an awareness-raising meeting for education system officials on the application of laws relating to access to literacy and vocational training for people with disabilities | 0% |
| **Strengthening the Financial Capacity (Employment) of PD-NTDs and Caregivers through Periodic Cash Transfers, Development of AGR and Promotion of Self-Employment** | |
| Activity | Level of Implementation |
| Identify partners for financial and technical support for people with disabilities | 100% |
| Establish collaboration agreements with two institutions specializing in the promotion of self-employment | 100% |
| Establish a fund to guarantee access to micro-credits for PD-NTDs | 0% |
| Identify AGR needs for PSH-MTN | 100% |
| Identify PD-NTDs who should benefit from emergency cash transfers | 100% |
| Advocate with institutions responsible for monetary transfers to take into account PD-NTDs | 100% |
| Establish an intersectoral monitoring committee | 100% |
| Organize an advocacy meeting with the labor inspectorate so that public and private employers apply the laws in favor of the employment of PD-NTDs | 100% |

Axe 2: Fight against the Stigma and Promotion of Respect of the Rights of PD-NTDs

| Improve the Knowledge of the Population including PD-NTDs and Caregivers on the Regulations protecting People with Disabilities | |
| --- | --- |
| Activity | Level of Implementation |
| Organize awareness sessions through community radios (integrated into awareness activities) | 100% |
| Produce and distribute 500 awareness posters on laws and regulations protecting people with disabilities, specialized care structures and rehabilitation/readaptation services. | 0% |
| **Promoting Rights of PD-NTDs** | |
| Activity | Level of Implementation |
| Develop and distribute through WhatsApp to PD-NTDs and their caregivers a video that presents the rights of people with disabilities. | 100% |
| Organize an advocacy meeting with the authorities in charge of territorial administration and the Regional Directors of the target ministries to raise awareness of the laws and regulations that protect people with disabilities | 100% |
| Establish a partnership with the Bouaké legal clinic for the management of denials of rights of PD-NTDs | 100% |
| Organize an advocacy meeting with the Ministry of Justice in Bouaké to obtain its support for the promotion of the rights of PD-NTDs | 100% |
| **Strengthening the Fight against Stigma and Social Discrimination & Promoting the Inclusion and Participation of PD-NTDs** | |
| Activity | Level of Implementation |
| List and disseminate existing laws and regulations protecting PD-NTDs | 100% |
| List the stigmatizing practices experienced in the project area by PD-NTDs | 100% |
| Organize 5 awareness-raising meetings for community leaders on the abandonment of stigmatizing practices by community members | 20% |

Axe 3: Increase community mutual support amongst PD-NTDs

| **Strengthening the Self-Help Capacities of PD-NTDs** | |
| --- | --- |
| Activity | Level of Implementation |
| Train a PD-NTD/FAHCI pool and caregivers in the psychological care of PD-NTDs – Psychological care module to be administered by Health District de Bouaké to the participants in half a day | 0% |
| Set up a support team for the preparation of admission files and referral of PD-NTDs to specialized structures | 100% |
| Train a pool of 25 people on NTDs to raise awareness on the recognition of suspicious signs, possible complications and management, as well as the laws and regulations that protect people with disabilities (Training of focal points) | 25% |
| Organize training for a pool of PD-NTDs in the promotion and defense of their rights. (Training of focal points) | 100% |
| Organize a meeting to promote self-help groups with PD-NTDs and their caregivers (Send messages via WhatsApp – identify a mutual aid focal point) | 100% |

Axe 4: Strengthening the Organizational Capacities of FAHCI with an Objective to Sustain the Pilot Strategy’s Actions

| **Strengthening the Associative Management and Resource Mobilization Capacities to Sustain Pilot Strategy Actions** | |
| --- | --- |
| Activity | Level of Implementation |
| Organize quarterly exchange meetings with the staff with a view to improving the administrative, financial and programmatic management of the association | 100% |
| Partner with a firm to develop and implement a resource mobilization plan to support the execution of the plan | 0% |
| **Ensure the coordination, monitoring and evaluation of the project** | |
| Activity | Level of Implementation |
| Identify focal points at each district level for the implementation of the project | 100% |
| Develop and implement a project monitoring and evaluation plan | 100% |
| Equip the coordination unit with IT equipment | 100% |
| Organize monthly project monitoring meetings with stakeholders | 100% |
