# Supplementary material for: People-centered strategies to mobilize people living with disabilities due to Neglected Tropical Diseases (PD-NTDs) to influence policy and programs: A mixed-methods study in Côte d’Ivoire
Source: PLoS Negl Trop Dis. 2025 Sep 8;19(9):e0013485. doi: 10.1371/journal.pntd.0013485 (PMC12431663; doi:10.1371/journal.pntd.0013485)
Supplement: S4 Table — (DOCX) [file pntd.0013485.s004.docx]

**REPUBLIC OF CÔTE D'IVOIRE**

UNION – DISCIPLINE - LABOR

**TOOLKIT**

**AND SCALE-UP STRATEGY**

**Identification of Strategies to Increase the Influence of People Affected by Neglected Tropical Diseases on their Access to Care Services in Côte d’Ivoire (the Ivory Coast)**

**February 2024**

**TABLE OF CONTENTS**

[LIST OF ABBREVIATIONS 5](#_Toc168419135)

[THANKS 6](#_Toc168419136)

[PREFACE 7](#_Toc168419137)

[INTRODUCTION 8](#_Toc168419138)

[I. BACKGROUND AND RATIONALE 9](#_Toc168419139)

[II. OBJECTIVE OF CREATING THE TOOLKIT 10](#_Toc168419140)

[2.1. General Objective 10](#_Toc168419143)

[2.2. Specific Objectives 10](#_Toc168419144)

[III. TOOLKIT DEVELOPMENT METHODOLOGY 10](#_Toc168419145)

[IV. PROJECT MANAGEMENT PROCESS 11](#_Toc168419146)

[V. STEPWISE TOOL PROPOSAL 11](#_Toc168419147)

[Step 1: Selection of stakeholders including the organization of Persons with Disabilities who will lead the implementation of the strategy. 11](#_Toc168419151)

[**a. Stakeholder selection technique** 11](#_Toc168419152)

[**b. Agreement among stakeholders** 13](#_Toc168419153)

[**c. Criteria for selection of an organization of People Disabled by NTDs** 14](#_Toc168419154)

[**d. Three-party agreement among the Government, the organization of Persons with Disabilities, and the technical and financial partners** 15](#_Toc168419155)

[Step 2: Participatory development of the project with the contribution of all stakeholders 16](#_Toc168419156)

[**Reference framework** 16](#_Toc168419157)

[Step 3: Launch of the project with the establishment of management bodies 17](#_Toc168419158)

[**a.** **Scientific Committee Establishment Framework** 17](#_Toc168419159)

[**b.** **Project Team Establishment Framework** 17](#_Toc168419160)

[Step 4: Conducting the baseline survey with the participation of People with Disabilities to identify needs, experiences and knowledge on the legislation protecting Persons with Disabilities. 18](#_Toc168419161)

[**Outline of the Study Protocol** 18](#_Toc168419162)

[Step 5: Participatory development of the strategy to “Increase the influence of people affected by NTDs on access to healthcare, physical, psychosocial and economic rehabilitation services”. 20](#_Toc168419163)

[**a.** **Strategy Development Framework** 20](#_Toc168419164)

[Steps 6: Recruitment from among Persons with Disabilities of representatives who can ensure the implementation of the strategy. 21](#_Toc168419165)

[**a.** **Call for Applications Framework** 21](#_Toc168419166)

[*b.* *Candidate Selection Report Framework* 22](#_Toc168419167)

[Step 7: Technical and financial support for the implementation of the strategy (coaching, monitoring and evaluation) 23](#_Toc168419168)

[**a.** **Project Information Tool Framework** 23](#_Toc168419169)

[**b.** **Pw/D-NTDs Census Framework** 23](#_Toc168419170)

[**c.** **Official Notes Template** 24](#_Toc168419171)

[**d.** **Activity and Meeting Reporting Framework** 25](#_Toc168419172)

[**e.** **Action Plan Development Framework** 25](#_Toc168419173)

[**f.** **Framework for the development of awareness and advocacy messages for use by community leaders** 26](#_Toc168419174)

[**g.** **Framework for the development of stakeholder commitment statement** 27](#_Toc168419175)

[**h.** **Framework for collaboration agreements with regional institutions** 28](#_Toc168419176)

[**i.** **Framework for terms of reference for the coordination unit between the project implementation team and the Management of the Organization of Persons with Disabilities** 29](#_Toc168419177)

[**j.** **Framework for the development of a logical framework, monitoring plan and theory of change diagram** 30](#_Toc168419178)

[**k.** **Framework for the development of an evaluation grid on the effectiveness of the project implementation:** 30](#_Toc168419179)

[**l.** **Funding Request Framework:** 31](#_Toc168419180)

[**m.** **Financial reporting framework** 32](#_Toc168419181)

[**n.** **Property donation report framework** 32](#_Toc168419182)

[**o.** **Project amendment request note template** 33](#_Toc168419183)

[**p.** **Project Progress Reporting Framework** 34](#_Toc168419184)

[**q.** **End-of-project reporting framework** 34](#_Toc168419185)

[**r. Project Closure Checklist** 35](#_Toc168419186)

[VI. PROJECT SCALE-UP STRATEGY 36](#_Toc168419187)

[CONCLUSION 37](#_Toc168419188)

[BIBLIOGRAPHIC REFERENCES 38](#_Toc168419189)

[APPENDICES : 39](#_Toc168419190)

# **LIST OF ABBREVIATIONS**

| CESI SARL | : | Cabinet d’Études Statistiques et Informatique [Statistical & IT Consulting Firm, Ltd] |
| --- | --- | --- |
| CIV | : | Côte d’Ivoire (the Ivory Coast) |
| COOPEC |  | Cooperative dèEpargne et de Credi (Savings and Credit Cooperative) |
| COPTIMENT | : | Croissance et Optimisation des Entreprises (Business Development and Optimization) |
| DP | : | Disabled Person (Person with Disabilities) |
| FAHCI | : | Ivorian Federation of Disabled Persons’ Associations |
| HAT | : | Human African Trypanosomiasis |
| HIV |  | Human Immunodeficiency Virus |
| HMIS | : | Health Management Information System |
| KAPBs | : | Knowledge, Attitudes, Practices and Beliefs |
| KAPs | : | Knowledge Attitudes and Practices |
| LF | : | Lymphatic Filariasis |
| LRI | : | Leprosy Research Initiative |
| MDA | : | Mass Drug Administration |
| MMDP | : | Morbidity Management and Disabilities Prevention |
| MT | : | Mass Treatment |
| NGO | : | Non-Governmental Organization |
| NTDs | : | Neglected Tropical Diseases |
| PD/NTD |  | Person Disabled by Neglected Tropical Disease |
| PNEL | : | National Leprosy Eradication Program |
| PNLMTN CP | : | National Program to Combat Neglected Tropical Diseases with Preventive Chemotherapy |
| PNLUB | : | National Buruli Ulcer Control Program |
| Pw/D-NTDs |  | Person with Disabilities due to Neglected Tropical Diseases |
| RTI | : | Radio Television Ivoirienne (Ivorian Broadcasting Corporation) |
| SD | : | Skin Disease |
| SO | : | Strategic Objective |
| WHO | : | World Health Organization |

# **AKNOWLEDGEMENT**

We extend our sincere gratitude to all the institutions that contributed to the development of this toolkit, namely:

Health Programs in Côte d'Ivoire (the Ivory Coast) dedicated to the fight against Neglected Tropical Diseases, in particular the National Leprosy Eradication Program (PNEL), the National Buruli Ulcer Control Program (PNLUB), including other Endemic Ulcerative Skin Diseases, and the National Program to Combat Neglected Tropical Diseases with Preventive Chemotherapy (PNLMTN-CP) for their generous technical contributions and political support.

The National Federation of Disabled Persons’ Organizations (FAHCI) for its leadership in the implementation of the strategy and the drafting of the document.

Leprosy Research Initiative (LRI), Effect Hope Canada and COPTIMENT (Business Development and Optimization Consulting Firm) for their technical and/or financial support.

# **PREFACE**

“**Nothing about us without us!**” This was the moto that guided the stakeholders of the DIGNITE project. This promise was kept throughout the design, implementation, and monitoring and evaluation of the project.

From the beginning, we were a leading stakeholder, contributing to the design and development of the final project document, which was then submitted and approved. We were also involved in the implementation of the project, participating in management bodies such as the Scientific Committee and the Management Team, mainly composed of Persons with Disabilities.

We are delighted with this inclusive approach which has allowed us to familiarize ourselves with the fight against Neglected Tropical Diseases (NTDs) and their socio-economic consequences.

From now on, we are an integral part of all initiatives aimed at improving the quality of life of Persons with Disabilities due to NTDs.

We believe that the programs in charge of these diseases now trust us enough to influence policies and programs in favor of people disabled by NTDs, thanks to this pilot study that we conducted together.

We would like to thank all the stakeholders, particularly the project initiators, the Leprosy Research Initiative (LRI), financial partner, as well as Effect Hope Canada and Croissance et Optimisation des Entreprises (COPTIMENT) for their technical support. We also express our gratitude to the NTDs programs which supported the project by providing their political and administrative support, in particular the National Leprosy Eradication Program (*PNEL*), the National Buruli Ulcer and Endemic Ulcerative Skin Diseases Control Program (*PNLUB-MCUE*), and the National Program to Combat Neglected Tropical Diseases with Preventive Chemotherapy (*PNLMTN-CP*).

This experience, which we lived with great enthusiasm, opens up prospects for greater involvement of Disabled Persons’ associations in the fight against Neglected Tropical Diseases.

**Hon. Minister Raphaël DOGO**,

President of the FAHCI

# **INTRODUCTION**

This work aims to share our experience, the process, and the management tools that we used during the development of an operational research project which focused on “*Exploring strategies to effectively mobilize persons with disabilities due to Neglected Tropical Diseases (NTDs) in a people-centered approach so that they influence policies and programs aimed at improving their access to healthcare, physical, psychological, and socio-economic rehabilitation services*”.

Our hope is to see other organizations take inspiration from it to improve the quality of life of people affected by neglected tropical diseases.

This is a modest contribution to the fight against the socio-economic consequences resulting from NTDs. Your suggestions and contributions will be much appreciated for the continuous improvement of its content and dissemination.

In this paper, we will discuss the following sequentially: first, we will review the background and rationale (I), next we will discuss the purpose of developing the toolkit (II). After that, we will discuss the development methodology (III) followed by the project management process (IV). We will then explore the proposed tools in stages (V), and finally, we will propose a project scale-up strategy (VI).

# **I. BACKGROUND AND RATIONALE**

Côte d'Ivoire is a sub-Saharan African country affected by multiple NTDs. The country is endemic to fourteen (14) NTDs out of the twenty (20) identified by the World Health Organization (WHO).

To respond to the scale of these NTDs, the country has set up five (5) special health programs dedicated to NTDs.

The current Mass Drug Administration (MDA) programs for NTDs with preventive chemoprophylaxis, particularly onchocerciasis, lymphatic filariasis, trachoma, soil-transmitted helminthiasis and bilharziasis, are not supported by a strong component of Morbidity Management and Disabilities Prevention (MMDP). Unfortunately, people affected by NTDs identified during MDA programs do not all have access to adequate healthcare services, suffer from late diagnosis and thus develop disabilities.

For NTDs falling under Innovative and Intensified Disease Management cases (IDM-NTDs) such as leprosy, Buruli ulcer and yaws, "Innovative and Intensive Disease Management" programs continue to reveal in their reports a significant number of cases with disabilities already established at the time of diagnosis. For leprosy, over the last three years, the program has revealed approximately 20% of new cases of grade 2 disability (Annual Report 2019). For Buruli ulcer, 26% of cases were diagnosed with category 3 lesions leading to permanent disabilities (Annual Report 2018).

The quality of available data on the number of people disabled by NTDs to fully ascertain the magnitude of the issue remains a challenge. Efforts to improve the Health Management Information System (HMIS) and mapping of active cases are still under way.

There is still a lack of prioritization of people with disabilities in development policies. Ensuring that persons with disabilities due to NTDs (Pw/D-NTDs) are not left behind will contribute to achieving the Sustainable Development Goals^^[[1]](#footnote-1)^^ (SDGs) 1, 3, 5, 8 and 11, and improve the control and eradication of NTDs^^[[2]](#footnote-2)^^.

The health system is focused on preventive and curative care services and cannot manage alone the long-term physical and socio-economic consequences on people affected by NTDs. This is partly due to the absence of a multi-sectoral coordination scheme involving several human development sectors. It could also be the result of competing priorities for limited resources and the lack of expression of demand and influence of people affected by NTDs on policy and program development, and resource allocation to address related socio-economic consequences.

To address this situation, the operational research project was developed to mobilize people affected by NTDs so that they influence government policy and multisectoral programs with a view to improving their access to healthcare, and psychosocial, economic and physical rehabilitation services.

The implementation of the project provided a good understanding of the needs, experiences, and level of knowledge of Persons with Disabilities due to NTDs (Pw/D-NTDs) on the legislation that protects them and promotes their rights.

Using these results, a strategy centered on persons with disabilities due to NTDs was developed and implemented by the latter through a pilot project dubbed “Dignity” over a period of 17 months.

The successes, challenges and lessons learned during implementation, enhanced by the inputs from other players, enabled to develop this toolkit.

# **II. OBJECTIVE OF CREATING THE TOOLKIT**


## **General Objective**

The general objective is to contribute to the reduction of the socio-economic consequences associated with NTDs.

## **Specific Objectives**

- 1. Propose an approach centered on people disabled by NTDs to improve their access to healthcare, and psychological and socio-economic rehabilitation services.
  2. Provide tools for the implementation of the approach.
  3. Propose a scale-up strategy for the approach.

# **III. TOOLKIT DEVELOPMENT METHODOLOGY**

The document uses a descriptive approach to present the processes and tools used throughout the project life cycle, from its design to its final evaluation. The documentary review includes both the documents used during the implementation of the project and those resulting from the literature review.

Strengths and weaknesses are analyzed based on our specific context. The design of the proposed processes and tools incorporates necessary revisions, informed by the successes, challenges and lessons learned during the project implementation. In addition, it draws upon the identified strengths of experiences from other interventions deemed relevant to our project.

# **IV. PROJECT MANAGEMENT PROCESS**

The process is composed of seven steps described below:

- **Step 1:** Selection of stakeholders including the organization of Persons with Disabilities who will lead the implementation of the strategy.
- **Step 2:** Participatory development of the project with the contribution of all stakeholders.
- **Step 3:** Launch of the project with the establishment of management bodies.
- **Step 4:** Conduct of the baseline survey with the participation of Persons with Disabilities to identify needs, experiences and knowledge on the legislation that protects People with Disabilities.
- **Step 5**: Participatory development of the strategy to “Increase the influence of people affected by NTDs on access to healthcare, and physical, psychosocial and economic rehabilitation services”.
- **Steps 6**: Recruitment, from among Persons with Disabilities, of representatives who can ensure the implementation of the strategy.
- **Step 7**: Technical and financial support for the implementation of the strategy (coaching, monitoring and evaluation).

# **V. STEPWISE TOOL PROPOSAL**

In this chapter, depending on the implementation steps, we suggest the most important tools. This list is not exhaustive.


## **Step 1: Selection of stakeholders including the organization of Persons with Disabilities who will lead the implementation of the strategy.**

### **a. Stakeholder selection technique**

There are several techniques used in project management practice to identify, classify, and select key stakeholders. It is important to choose the technique that suits you best. Here are some:

**● Stakeholder analysis**: this involves identifying all potential stakeholders, whether internal or external to the project. Once identified, stakeholders can be assessed based on their interest, influence, and power in relation to the project.

Then identify the following three levels of involvement:

- Primary (direct): directly affected, including primary beneficiaries.
- Secondary: indirectly affected.
- Key: actively involved in the project decisions and management.

**● Power/Interest Matrix**: simple but powerful to classify stakeholders based on their level of power over the project and their interest in it. This helps determine which stakeholders require special attention and what level of engagement is needed for each.

- Power of stakeholders: political, social, and economic status, level of organization, control of strategic resources, decision-making process, formally or informally, power relationship with stakeholders, importance in the success of the project.
- Stakeholder interest: expectations of the project, attractive benefits, resources willing to mobilize and conflict with project objectives.

**● Stakeholder mapping**: consists of mapping the relationships between the different stakeholders involved in the project. This helps visualize connections between and among stakeholders and identify potential alliances or conflicts of interest.

| What for | : To map stakeholders |
| --- | --- |
| What is its content | : Specifies the categories, the actions to be undertaken regarding these stakeholders and the list of identified stakeholders |
| How to use the tools | : In groupwork, with groups of stakeholders identified and listed out of consensus, by types relevant to the project |
| When | : Before and during the launch of the project and it can be updated at any time |
| Where | : In a working session |
| Who | : The project manager organizes the working session |
| With whom | : The other members of his/her project team |

| **TYPES OF STAKEHOLDERS** | **LISTS OF STAKEHOLDERS IDENTIFIED FOR THE PROJECT** |
| --- | --- |
| Who will be affected (positively and negatively) by your project? |  |
| Who has an interest in your project? |  |
| Who has power over your project? |  |
| Who wants your project to fail? |  |
| Who wants your project to succeed? |  |

**● Interviews and consultations**: Individual interviews or group consultations with potential stakeholders can provide valuable information about their expectations, concerns, and level of engagement. This also helps establish effective communication from the start of the project.

| What for | : To regulate labor relations among stakeholders |
| --- | --- |
| What is its content | : See template below |
| How to use the tool | : Complete all sections |
| When | : At any time during the project and can be readapted |
| Where | : Working sessions |
| Who | : The project coordinator |
| With whom | : The project team and stakeholders |

**Stakeholder Interview Guide**

- INTRODUCTION: Greetings and brief introduction to the purpose of the interview.
- INVOLVEMENT IN THE PROJECT: Role, date of entry, responsibilities and benefit received from the project?
- PROGRESS ASSESSMENT: Main successes or achievements, challenges encountered, observation on collaboration and communication among the different stakeholders, aspects for improvement?
- FEEDBACK AND SUGGESTIONS: Suggestions, specific areas for needed changes, additional resources or types of support beneficial to the project?
- CONCLUSION: Acknowledgments

### **b. Agreement among stakeholders**

| What for | : To regulate working relations among stakeholders |
| --- | --- |
| What is its content | : See template below |
| How to use the tool | : Complete all sections |
| When | : Before the launch of the project |
| Where | : Online |
| Who | : The representative of the institution responsible for managing the project |
| With whom | : Other contracting parties and a legal assistance if applicable |

The agreement, in the case of the project, is a contractual document which makes it possible to regulate the working relations of the stakeholders including the funder, the recipient institution responsible for the execution of the project, the other primary, secondary, and key stakeholders.

It is a legal document of which main structure (16 chapters) is as follows.

**Agreement among Stakeholders template**

- Title of the project subject to the agreement
- Contracting parties
- Terms of agreement
- Period and duration
- Obligations and responsibilities of the contracting parties
- Agreement on visibility of contracting organizations
- Advertising and publishing
- Intellectual properties and confidentiality
- Offense
- Amendment
- Force majeure and termination of the agreement
- Law applicable in the management of the agreement
- Entire agreement
- Divisibility
- Responsibility and security
- Funding and other project resources
- Risks linked to fluctuations in foreign currencies and exchange rates
- Guarantee
- Signature

### **c. Criteria for selection of an organization of People Disabled by NTDs**

| What for | : Establish collaboration with an organization of people disabled by NTDs to ensure leadership in the implementation of the project and carry their voice |
| --- | --- |
| What is its content | : see selection template below |
| How to use the tool | : Complete all sections |
| When | : Before the launch of the project |
| Where | : Preparation and circulation for validation |
| Who | : The representative of the institution responsible for managing the project |
| With whom | : Other stakeholders |

Regarding the organization of Persons with Disabilities, the following criteria must guide the choice.

**Standard criterion template for the selection of an organization of Pw/D-NTDs**

1. Duly constituted and legally authorized organization
2. Members are people with disabilities due to NTDs or other causes as applicable
3. Organization aiming at improving the well-being, living conditions and defense of the rights of Persons with Disabilities
4. Willingness to participate in the study.
5. Organization with a presence on the project site (headquarters or decentralized agency)
6. Organization with a good reputation.

### **d. Three-party agreement among the Government, the organization of Persons with Disabilities, and the technical and financial partners**

| What for | : To regulate working relations between the Government, the organization of Persons with Disabilities, and technical and financial partners |
| --- | --- |
| What is its content | : See template below |
| How to use the tools | : Complete all sections |
| When | : Before the launch of the project |
| Where | : Preparation and circulation for validation |
| Who | : The representative of the institution responsible for managing the project |
| With whom | : Other contracting parties and a legal assistance if applicable |

The collaboration agreement in the case of the project is a tripartite agreement among the Government, the organization of Persons with Disabilities, and the technical and financial partners. It is a legal document of which main structure is as follows.

**Three-party Agreement template**

- Identification of contracting parties
- The preamble
- The general provisions including the purpose and value of the preamble
- The obligations of the parties
- Miscellaneous and final provisions
- Signature

## **Step 2: Participatory development of the project with the contribution of all stakeholders**

### **Terms of Reference framework**

| What for | : To provide a guideline, an idea of ​​the activity to guests and other stakeholders. |
| --- | --- |
| What is its content | : See terms of reference template below |
| How to use the tool | : Complete all sections |
| When | : Before each activity, attach them to the invitation letters |
| Where | : At headquarters |
| Who | : The project coordinator |
| With whom | : The project team |

**Terms of reference template**

- Background and rationale
- General objective and specific objectives
- Expected results
- Methodology
- Dates
- Location and list of participants
- Working agenda
- Budget

## **Step 3: Launch of the project with the establishment of management bodies**

### **Scientific Committee Establishment Framework**

| What for | : For expert advice, guidance, and support in project implementation |
| --- | --- |
| What is its content | : See scientific committee establishment template below |
| How to use the tool | : Complete all sections |
| When | : during the launch of the project |
| Where | : Meeting hall |
| Who | : Program in charge |
| With whom | : With representatives of other stakeholders |

The document establishing the scientific committee is structured as follows:

**Scientific Committee Establishment template**

- Official Order ref. ID
- Project Title
- General Provisions
- Mission Statement and Organizational Diagram
- Responsibilities and Operation
- Final Provisions
- Signature

- Copy recipients (other interested parties)

### **Project Team Establishment Framework**

| What for | : To specify the missions, responsibilities and functioning of the project team |
| --- | --- |
| What is its content | : see project team establishment template below |
| How to use the tool | : Complete the different sections. |
| When | : Before setting up the project team |
| Where | : Preparation and circulation for validation |
| Who | : Program in charge |
| With whom | : With representatives of other stakeholders |

The document establishing the project team is structured as follows:

**Project Team Establishment template**

- Official Order ref. ID
- Project Title
- General Provisions
- Mission Statement and Organizational Diagram
- Responsibilities and Operation
- Final Provisions
- Signature
- Amplifiers

## **Step 4: Conducting the baseline survey with the participation of People with Disabilities to identify needs, experiences and knowledge on the legislation protecting Persons with Disabilities.**

### **a.Outline of the Study Protocol**

| What for | : To design the methodology and data collection tools for the implementation of the baseline study |
| --- | --- |
| What is its content | : see table of contents below |
| How to use the tool | : Complete the different sections indicated in the table of contents |
| When | : Before the baseline study |
| Where | : Preparation of the draft by the investigator and circulation among stakeholders |
| Who | : The investigator |
| With whom | : The scientific committee for the validation of the draft |

**Protocol Table of Contents template**

**List of abbreviations and acronyms**

**I- Context of the Study**

I-1 Context of the Project

I-2 Scientific Background/Rationale of the Study

I-3 Introduction to the Implementing Organization

**II- Objectives of the Research**

**III- Methodology of the Research**

III-1 Organizational Framework

III-2 Areas of the Study

III-3 Type, Study Methodology and Data Collection

*III-3-1 Literature Review*

*III-3-2 Qualitative Survey*

*III-3-3 Quantitative Survey*

**IV- Planning of the Survey**

IV-1 Protocol Development and Validation

IV-2 Data Entry Form Design and Pre-Test

IV-3 Field Exploratory Mission

IV-4 Recruitment and Training of Investigators

IV-5 Pilot Survey

**V- Data Collection**

V-1 Field Data Collection

V-2 Usage of Mobile Technology for Data Collection

V-3 Collection Supervision

V-4 Collection Monitoring

**VI- Data Processing and Analysis, Reporting**

VI-1 Data Cleansing and Statistical Analysis

VI-2 Reporting

**VII- Data and Work Quality Control**

**VIII- Ethical Considerations**

VIII-1 Data Confidentiality, Privacy and Storage

VIII-2 Informed and Written Consent

VIII-3 Data Storage and Deletion

VIII-4 Surveyees’ Compensation for their Voluntary Participation

VIII-5 Involvement and Participation of People Affected by NTDs

VIII-6 Risk Management Procedure

VIII-7 Any Other Ethical Aspects

VIII-8 Anticipated Impacts

**IX- Study Timeframe**

**X- Study Team**

X-1 Member List

X-2 Member Profile Information

**XI- Budget**

**Appendices**

Questionnaire

Interview Guide

Information Note

Informed and Written Consent Form

***b.Framework for the development of an evaluation report***

| What for | : To prepare the evaluation report on the performance, results and impact of the project |
| --- | --- |
| What is its content | : See table of contents below |
| How to use the tool | : Complete all sections |
| When | : After data collection and analysis |
| Where | : Preparation of the draft and circulation for validation |
| Who | : The investigator |
| With whom | : The project team and other stakeholders for review and validation |

**Evaluation Report Plan Template**

| List of abbreviations and acronyms |
| --- |
| Executive Summary |
| I- Introduction |
| I-1 Context of the Project |
| I-2 Scientific Background/Rationale of the Study |
| I-3 Implementation |
| II- Objectives of the Evaluation |
| II-1 General Objective |
| II-2 Specific Objectives |
| III- Methodology |
| III-1 Methods and Tools |
| **III-1-1 Documentary Review** |
| **III-1-2 Qualitative Survey** |
| **III-1-3 Area and Targets of the Evaluation** |
| III-2 Data Collection |
| III-3 Data Processing and Reporting |
| IV- Pilot Project Final Evaluation Results |
| IV-1 Overview of the Project |
| IV-2 Project Relevance Analysis (against Global and Local Context) - Specification |
| IV-3 Project Consistency Analysis (internal and external) |
| IV-4 Project Efficiency Analysis |
| **IV- 4-1 Overall Level of Implementation of the Project Activities** |
| **IV- 4-2 Level of Attainment of the Project Objectives** |
| IV-5 Analysis of the Impacts of the Project on People Affected by NTDs |
| **IV-5-1 In terms of Knowledge and Attitudes** |
| **IV-5-2 In terms of Health Status of NTD-affected People** |
| **IV-5-3 In terms of Socio-economic Situation of NTD-affected People** |
| **IV-5-4 In terms of Stakeholder Satisfaction** |
| **IV-5-5 In terms of Pre- vs. Post-Project Situational Analysis** |
| IV-6 Strengths and Weaknesses Analysis of the Project |
| IV-7 Analysis of the Potential Sustainability of the Project Activities |
| IV-8 Lesson Learned and Best Practices |
| Conclusion and Recommendations for the Sustainability and Survival of the Project |
| Bibliography |
| Appendices |
| Information Note |
| Informed and Written Consent Form |
| Confidentiality Clause |
| Data Collection Tools |

## **Step 5: Participatory development of the strategy to “Increase the influence of people affected by NTDs on access to healthcare, physical, psychosocial and economic rehabilitation services”.**

### **Strategy Development Framework**

| What for | : To guide the development of the strategy |
| --- | --- |
| What is its content | : See table of contents below |
| How to use the tool | : Complete all sections |
| When | : Before implementing the strategy |
| Where | : Preparation and circulation for validation |
| Who | : Project team |
| With whom | : Other stakeholders for review and validation |

| **Strategy Drafting template**  List of abbreviations and acronyms   1. Background and rationale 2. Project development methodology 3. Project objective 4. Strategic thrust and objectives 5. Outcome mapping 6. Objectives, activities and schedule 7. Monitoring and evaluation plan   Appendices | …………………………………………. 3  …………………………………………. 4 …………………………………………. 5 …………………………………………. 6 …………………………………………. 6 …………………………………………. 8  …………………………………………. 9  ………………………………………… 15  ………………...………………………. 25 |
| --- | --- |

*For guidance purposes, the strategy contains four main thrusts, as follows:*

**Strategy direction template**

- Advocacy and awareness
- Fight against stigma, discrimination and promotion of the rights of persons with disabilities
- Promotion of mutual assistance among disabled people
- Strengthening the organizational capacities of the associations of persons with disabilities

## **Steps 6: Recruitment from among Persons with Disabilities of representatives who can ensure the implementation of the strategy.**

### **Call for Applications Framework**

| What for | : To develop the call for applications for the recruitment of a project manager, as well as an administrative and financial assistant. |
| --- | --- |
| What is its content | See details below |
| How to use the tool | : Complete all sections |
| When | : Before recruiting project staff |
| Where | : At headquarters |
| Who | : The head of the project management team prepares the draft |
| With whom | : The scientific committee validates the draft |

As soon as the draft is validated, the call for applications is distributed, in particular in the networks of Persons with Disabilities.

The applications received are processed by a committee including representatives of organizations of disabled people for the selection of the best profiles.

### **Candidate Selection Report Framework**

| What for | : Report on the selection of the best candidate profile for the positions of project manager, and administrative and financial assistant. |
| --- | --- |
| What is its content | : See details below |
| How to use the tool | : Complete all sections |
| When | : After recruitment |
| Where | : At headquarters |
| Who | : The head of the project management team |
| With whom | : With the recruitment committee |

**Candidate Selection Report template**

- Background and rationale
- Selection methodology
  1. Pre-selection based on the review of application packages for the positions of project manager, and administrative and financial assistant of the DIGNITE project. At the end of this step the three best profiles are selected.
  2. Selection of shortlisted candidates for interview by a recruitment panel of three members. At the end of this stage, the three best profiles are ranked in order of merit based on the total grades.
  3. Individual contacts are made in order of merit starting with the top candidate to discuss their salary expectations and availability for the start of the project. Reference check is also completed.
  4. Decision.

The candidate for whom the salary expectation, availability for the start of the project and reference check meet the project requirements will be proposed for recruitment.

## **Step 7: Technical and financial support for the implementation of the strategy (coaching, monitoring and evaluation)**

### **Project Information Framework**

| What for | : For awareness and publicity about the project |
| --- | --- |
| What is its content | : See the information tools template below |
| How to use the tools | : Complete all sections |
| When | : Before and during project implementation |
| Where | : Headquarters meeting hall |
| Who | : The project coordinator |
| With whom | : His/her project team |

**Information Tools template**

- Background and rationale
- Main needs of disabled people identified during the study phase
- Thrusts and objectives of the strategy
- Project implementation area
- Implementing institution.

**Call for applications template**

The “Dignity Project” for the identification of strategies aimed at increasing the influence of people affected by Neglected Tropical Diseases (NTDs) on access to physical, economic and psycho-social rehabilitation services in Ivory is a project designed following a baseline study which identified the needs and experiences of people disabled by NTDs in Côte d'Ivoire.

The project will be implemented in the Gbêkê health region. The duration of the project is one year covering the period from ……………to……………….

As part of the implementation of the project, the Federation of Associations of Disabled People of Cote d'Ivoire (FAHCI) is looking for people with the following profiles:

1. **Project Manager**
2. ***Roles and responsibilities***

- Ensure coordination with project stakeholders:
- Government institutions and partners from the for-profit and non-profit private sector
- FAHCI, the leprosy elimination program, COPTIMENT,
- Associations of people with disabilities by NTDs
- The scientific committee of the project
- Organize project coordination meetings
- Write and distribute meeting minutes;
- Validate cash flow forecasts
- Write periodic and detailed reports for donors and stakeholders
- Write end-of-project reports covering the entire project period
- Plan and mobilize stakeholders for the implementation of activities
- Write and evaluate the TOR for implementing project activities
- Monitor the execution of the decisions of the project's scientific committee;
- Inform the management team of any difficulties encountered in project management.
- Organize monitoring and evaluation of the project
- Ensure compliance with county ethics (CESI) requirements in the implementation of the project;
- Ensure compliance with the requirements of the Ministry of Health, the FAHCI and the lessor in the execution of the project.

1. ***Level of study***: Bachelor’s degree in economics and management, project management or any other diploma deemed equivalent
2. ***Professional experience*:** at least three (03) years in a project manager position or equivalent position.
3. **Financial and Administrative Manager :**
4. ***Roles and responsibilities***

- Share and archive documents relating to project activities;
- Monitor the execution of the decisions of the management team and the steering committee;
- Inform the management team of any difficulties encountered in project management.
- Ensure correspondence with the steering committee and project implementation stakeholders.
- Make funding requests for the implementation of project activities
- Write monthly, annual and interim financial reports for donors and stakeholders;
- Ensure the safety and proper functioning of project implementation equipment.
- Ensure compliance with the financial management requirements of the lessor,

1. ***Level of study*:**Bachelor's degree in economics and management, project management or any other diploma deemed equivalent.
2. ***Professional experience*:** at least three (03) years in an administrative and financial manager position.

The project team will be based in Bouaké and will work in close collaboration with FAHCI Bouake and Abidjan.

Applications from both sexes are strongly encouraged.

Interested persons are requested to send CV and cover letter no later than …………………at ……hours by email to ------

### **Pw/D-NTDs Census Framework**

| What for | : To identify Pw/D-NTDs, their needs and expectations |
| --- | --- |
| What is its content | : see information sheet template below |
| How to use the tools | : Complete all sections |
| When | : Before the launch of the census |
| Where | : At meeting session |
| Who | : the project coordinator is responsible for the drafting of the TOR |
| With whom | : The project team |

The census form for people disabled by Neglected Tropical Diseases includes the following items:

**Census Form for people disabled by Neglected Tropical Diseases template**

- Personal data of the respondent including family name, other names, date of birth, place of birth, place of residence.
- Marital status (married, single, divorced, widowed)
- Name of dependent child
- Phone number
- Name of caregiver
- Contact information of the caregiver
- Type of disability caused by NTDs
- Illness that caused the disability
- Needs: Health, psychological support, education (level of study and qualifications), initial education, skill training
- Income

### **Official Notes Template**

| What for | : To submit a request to or share information with an institution on the progress of activities. |
| --- | --- |
| What is its content | : See official note template below |
| How to use the tools | : Complete the sections |
| When | : Whenever it is necessary to submit a request or share information |
| Where | : At working sessions |
| Who | : The project coordinator |
| With whom | : The project team |

The official note is drawn up as follows:

**Official Note template**

- Name of the issuing institution
- Phone contact details
- Email
- Place
- Recipient institution details
- Subject
- Body of note
- Signature

### **Activity and Meeting Reporting Framework**

| What for | : To draft minutes of activities and meetings |
| --- | --- |
| What is its content | : see the outline below |
| How to use tools | : Complete the different sections of the outline with the notes taken during the activity. |
| When | : After each activity or meeting |
| Where | : At headquarters |
| Who | : A person designated at the start of the activity |
| With whom | : participants in the activity |

The content of the activity report is organized as follows:

**Activity Report template**

- Title of the activity
- Date
- Venue
- Participants
- Objectives / Agenda
- Progress / Results / Resolutions
- Recommendations
- Next steps

### **Action Plan Development Framework**

| What for | : For the programming of activities to achieve the project objectives |
| --- | --- |
| What is its content | : See template below |
| How to use the tools | : Complete all sections |
| When | : Before the project launch |
| Where | : Meeting hall |
| Who | : the project coordinator |
| With whom | : The project team and stakeholders |

**Workplan development template**

| **CODES** | **ACTIVITIES** | **TARGETS** | **PERSON IN CHARGE** | **CALENDAR** |
| --- | --- | --- | --- | --- |
| **FOCUS 1** | **ADVOCACY AND AWARENESS BUILDING FOR IMPROVED ACCESS TO PHYSICAL, PSYCHOSOCIAL, EDUCATIONAL AND ECONOMIC REHABILITATION SERVICES** | | | |
| **S.O. 1.1** | **STRATEGIC OBJECTIVE 1.1: WIN THE COMMITMENT OF ADMINISTRATIVE AUTHORITIES AND COMMUNITY LEADERS TO THE IMPLEMENTATION OF THE STRATEGY** | | | |
| **A1.1.1** | Organize a kick-off ceremony to share project details with administrative authorities, community, and organizations leaders in Bouake | Health and administrative authorities (Health, Home Affairs, Education, Technical and Vocational Training, Employment, Social Welfare, Economy and Finance, Justice) of the Gbeke health district | FAHCI with support from the scientific committee. | November 14 |

### **Framework for the development of awareness and advocacy messages for use by community leaders**

| What for | : To harmonize effective, authentic, and validated awareness-building messages on the impacts and actions of the project in order to avoid distortions of key messages |
| --- | --- |
| What is its content | : see template below |
| How to use the tools | : Complete the different sections of the template in a language accessible to community leaders. Comment on them and return them to community leaders so that they can use them to build awareness among the members of their community. |
| When | : Before the meetings with community leaders |
| Where | : Meeting hall |
| Who | : the project coordinator or a member of his/her team. |
| With whom | : The project team and other stakeholders |

**Awareness-building and Advocacy Message template**

**MESSAGE FOR LEADERS AND MEMBERS OF COMMUNITIES ENDEMIC TO NEGLECTED TROPICAL DISEASES**

**A. GENERAL INFORMATION ON NEGLECTED TROPICAL DISEASES**

Neglected Tropical Diseases (NTDs) are a group of 20 diseases. They occur primarily in tropical areas. They affect more than a billion people worldwide. When they are not quickly treated, they cause permanent disabilities. They deteriorate the quality of life of those affected and impoverish them. Twelve of these diseases are endemic in Côte d'Ivoire. These are **Human African Trypanosomiasis or sleeping sickness, Buruli ulcer, Leprosy, Scabies, Envenomation or snake bites, Guinea worm, Yaws, onchocerciasis, lymphatic filariasis, schistosomiasis, soil-transmitted helminthiasis or intestinal worms, and trachoma (add the corresponding disease name in the local language).**

**B. IMPACT OF DISABILITY ON THE COMMUNITY**

Many people disabled by these diseases live in poverty and are not offered the same education and job opportunities. Sometimes, they do not even benefit from healthcare (vaccinations) and are poorly fed. They sometimes suffer from stigma and rejection in their community. Caregivers are also victim of stigma, and also have to make sacrifices, as they dedicate time and resources to the one they are caring for.

**C. ACTIONS TO BE TAKEN TO FIGHT AGAINST THESE DISEASES AND THEIR CONSEQUENCES**

However, by focusing on people's 'abilities' rather than their 'disabilities' or disability, negative attitudes can change. Often, when people with disabilities receive the help they need, they can find a rightful place in the community.

- **Consult very early as soon as the first signs appear to obtain healing without after-effects.**
- **For people disabled by neglected tropical diseases:**
  - **Fight against socio-cultural barriers that lead to;**
    - Stigma
    - Discrimination
    - Social exclusion
  - **Promote**
    - The rights of persons with disabilities
    - Social inclusion
  - **Promote empowerment by facilitating**
    - Access to basic education and skill training
    - Access to employment and self-employment
    - Access to healthcare and physical rehabilitation services
    - Psychological support

### **Framework for the development of stakeholder commitment statement**

| What for | : To draft and give substance to the commitment of the project stakeholders |
| --- | --- |
| What is its content | : See declaration template below |
| How to use the tools | : Each institution commits by signing the statement to demonstrate its collaboration in the implementation of project activities |
| When | : Before the kick-off ceremony of the implementation of the strategy with targeted institutions |
| Where | : At headquarters |
| Who | : The project coordinator |
| With whom | : The project team and stakeholders |

**DECLARATION OF STAKEHOLDERS**

We, the participants in the kick-off ceremony of the active phase of the DIGNITY project, on "Mobilizing people affected by NTDs in order to influence government policy and multi-sectoral programs with a view to their better access to psychosocial, economic and physical rehabilitation services.

**Based on:**

- Resolution WHA73(33) adopted at the Seventy-third World Health Assembly, in which the new road map for neglected tropical diseases 2021-2030 was approved.
- World Health Organization (WHO)’s road map 2021-2030 which recognizes that NTD interventions are one of the "most cost-effective" investments in global public health and that NTDs are important tracers for measuring progress, considering disparities observed in universal health coverage and equitable access to quality health services.

**Recognizing the need for us to:**

- Guarantee universal access to early diagnosis and rapid treatment;
- Accelerate efforts towards the elimination and eradication of neglected tropical diseases by intensifying basic interventions;
- Set up support elements, for example by stimulating collaboration, promoting community mobilization, and carrying out awareness-building action.

**Taking note of the fact that:**

- We must take into consideration the needs of certain vulnerable groups, particularly people disabled by NTDs;
- We aim to ensure that basic health and social services such as education, access to employment, improvement of the living conditions of people affected by NTDs.
- The needs of Pw/D-NTDs are diversified in scope and multifaceted."

**We are committed to:**

- Support the implementation of the 'DIGNITY' project;
- Improve the physical, mental, and psychological well-being of persons with disabilities due to NTDs;
- Mobilize the necessary resources for the implementation of essential interventions contained in the strategy.
- Strengthen collaboration between the State of Côte d'Ivoire and partners engaged in the fight against neglected tropical diseases.
- Meet the psychological and socio-economic rehabilitation needs of Pw/D-NTDs in the Gbeke health district.
- Provide integrated diagnosis and treatment, including inclusive management of disability due to NTDs.
- Improve access to reference institutions.
- Reduce the discrimination and stigma experienced by Pw/D-NTDs.
- Support the economic rehabilitation of affected people and their caregivers;
- Increase advocacy with the government, donors, organizations, and institutions to improve the quality of life of Pw/D-NTDs

Date, Full Name, Position, Institution and Signature

### **Framework for collaboration agreements with regional institutions**

| What for | : To define the rights and duties in the collaboration for the implementation of the project by the signatory parties to the agreement. |
| --- | --- |
| What is its content | : See below |
| How to use the tools | : Complete the different sections |
| When | : Before the collaboration |
| Where | : At headquarters |
| Who | : The project coordinator |
| With whom | : Heads of regional institutions |

The collaboration agreement in the case of the project is an agreement between the regional unit for the implementation of the project for people with disabilities and other interested regional institutions.

It is a legal document of which main structure is as follows.

**Main structure of the collaboration agreement**

- Identification of contracting parties
- The preamble
- The general provisions including the subject and value of the preamble
- The rights and obligations of the parties
- Miscellaneous and final provisions
- Signature

### **Framework for terms of reference for the coordination unit between the project implementation team and the Management of the Organization of Persons with Disabilities**

| What for | : To formalize coordination between the project execution unit and the management of the organization acting as project initiator |
| --- | --- |
| What is its content | : See below |
| How to use the tools | : Complete the different sections of the terms of reference |
| When | : As soon as the project implementation team is established |
| Where | : At headquarters |
| Who | : The project coordinator |
| With whom | : The project team and the management of the organization |

The term of reference includes:

- **Term of Reference template**
- Background and rationale
- Objectives,
- Expected results,
- Work calendar,
- Member of the coordination unit
- Budget

### **Framework for the development of a logical framework, monitoring plan and theory of change diagram**

| What for | : To track project execution performance, results, and impact |
| --- | --- |
| What is its content | : See Excel file in appendix |
| How to use the tools | : Complete all sections |
| When | : Before starting the implementation of the strategy |
| Where | : At working sessions |
| Who | : Project team |
| With whom | : Other stakeholders for review and validation |

The template of the logical framework (log frame), the monitoring matrix and the theory of change diagram (outcome mapping) are Excel sheets, appended to this document.

### **Framework for the development of an evaluation grid on the effectiveness of the project implementation:**

| What for | : To measure the effectiveness of execution of project activities |
| --- | --- |
| What is its content | : See template below |
| How to use the tools | : Complete the sections of the template in a participatory manner based on the actions carried out to achieve the project's objectives at mid-term and at the end of the project. |
| When | : during the mid-term evaluation and the final evaluation |
| Where | : At working sessions |
| Who | : the investigator |
| With whom | : The project team and stakeholders |

1. **Project Implementation Effectiveness Evaluation Grid template**

| **ACTIVITY INDICATORS** | **EXECUTION STATUS** | **EXECUTION RATE** | **COMMENTS** | **Necessary Action before July 30** |
| --- | --- | --- | --- | --- |
| **ADVOCACY AND AWARENESS BUILDING FOR IMPROVED ACCESS TO PHYSICAL, PSYCHOSOCIAL, EDUCATIONAL AND ECONOMIC REHABILITATION SERVICES** | | | |  |
| **COMMITMENT OF ADMINISTRATIVE AUTHORITIES AND COMMUNITY LEADERS TO THE IMPLEMENTATION OF THE STRATEGY** | | | |  |
| Organize a kick-off ceremony to share project details with administrative authorities, community and organization leaders in Bouake | Completed | 100% | NTR |  |
| Win stakeholder commitment to improve the quality of life of Pw/D-NTDs | Completed | 100% | NTR |  |
| Organize at least one meeting with each institution to strengthen collaboration | Completed | 100% | NTR |  |

### **Funding Request Framework:**

| What for | : To request funds for project activities |
| --- | --- |
| What is its content | : See template below |
| How to use the tools | : Complete all sections |
| When | : Every two (2) months |
| Where | : At working sessions |
| Who | : The project coordinator |
| With whom | : The funds management team |

**Funding request submission template:**

| **Period : …………………** |  |  |  |  |  |
| --- | --- | --- | --- | --- | --- |
|  |  |  |  |  |  |
| **Description** | **Quantity** | **Unit Cost** | **Amount** | **Date** | **Good or Service Details** |
| **Remaining funds from previous period** |  |  |  |  |  |
|  |  |  |  |  |  |
|  |  |  |  |  |  |
|  |  |  |  |  |  |
| **Total expenditure to be made** |  |  |  |  |  |
| **Balance at start of period** |  |  |  |  |  |
| **Funds required for the period** |  |  |  |  |  |
|  |  |  |  |  |  |
| **Date: May 23, 2023** |  |  |  |  | **Date:** |
| **Name and Signature of Requester** |  |  |  |  | **Approval** |

### **Financial reporting framework**

| What for | : To prepare the bimonthly report of funds spent |
| --- | --- |
| What is its content | : See template below |
| How to use the tools | : Complete all sections |
| When | : Every two (2) months |
| Where | : At working sessions |
| Who | : The project coordinator |
| With whom | : The funds management team |

**Financial report template:**

| **Financial report** | | | | |  |
| --- | --- | --- | --- | --- | --- |
| **Period : …………** |  |  |  |  |  |
|  |  |  |  |  |  |
| **Description** | **Quantity** | **Unit Cost** | **Amount** | **Good or Service Details** | **Proof of expenditure** |
|  |  |  |  |  |  |
|  |  |  |  |  |  |
| **Total expenses incurred** |  |  |  |  |  |
| **Gap** |  |  |  |  |  |
|  |  |  |  |  |  |
| Date : |  |  |  | Date : |  |
| Authorized Person  and Signature |  |  |  | Approval Authority |  |
|  |  |  |  |  |  |

### **Projet property donation report framework**

| What for | : To document donated noncash items |
| --- | --- |
| What is its content | : see the main items of the report below |
| How to use the tools | : Complete all sections |
| When | : Any time a noncash item is donated as part of the implementation of the project |
| Where | : Meeting hall |
| Who | : The financial partner |
| With whom | : The project team |

**Property Donation Report template**

- Heading of the document:
- Date
- Venue
- Subject
- Project / Beneficiaries
- Participants
- Materials/Equipment
- Conditions of use of equipment:
- Signatories

### **Project amendment request note template**

| What for | : To submit a request note on amendment to the execution calendar, objective indicators, budget, stakeholders, etc. |
| --- | --- |
| What is its content | : See template below |
| How to use the tools | : Complete all sections |
| When | : Whenever necessary or on the submission of periodic reports |
| Where | : Meeting hall |
| Who | : The administrative officer of COPTIMENT |
| With whom | : The COPTIMENT team and project team |

The amendment request note includes the following items:

**Amendment Request Note template**

- The subject of the request
- The justification
- The financial impact on the project budget
- The method of financing this financial impact

### **Project Progress Reporting Framework**

| What for | : To prepare progress reports on project implementation |
| --- | --- |
| What is its content | : See the outline below and in the appendix. |
| How to use the tools | : Complete all sections |
| When | : During the project course, on a quarterly, half-yearly or annual basis |
| Where | : Working sessions |
| Who | : The project coordinator |
| With whom | : The project team |

**Project progress report**

**Project Milestones Report template**

- Project information
- Scientific progress
- Plans for the following year
- Summary of progress and plans communication to non-specialists
- Financial report
- Signature

### **End-of-project reporting framework**

| What for | : To prepare the end-of-project report |
| --- | --- |
| What is its content | : See the outline below and in the appendix |
| How to use the tools | : Complete all sections |
| When | : At the end of the project |
| Where | : Working sessions |
| Who | : The project coordinator |
| With whom | : The project team |

**End-of-project Report template**

- Project information
- Scientific achievement
- Project management
- Future perspective
- Financial management
- Signature

### **s. Project Closure Checklist**

| What for | : To inform project stakeholders and beneficiaries of the end of the project and fulfill all closure obligations |
| --- | --- |
| What is its content | : See checklist below |
| How to use the tools | : Complete all sections |
| When | : At the end of the project |
| Where | : Working sessions |
| Who | : The project coordinator |
| With whom | : The project team |

**Checklist template**

| **Proceedings** | **Completed** | **Pending** |
| --- | --- | --- |
| Final external evaluation |  |  |
| End-of-project narrative and financial report |  |  |
| End-of-project meeting with presentation of the final evaluation and end-of-project report |  |  |
| Closure of project accounts |  |  |
| Repayment of unused account balance |  |  |
| Management of equipment acquired for the implementation of the project |  |  |
| Official notification to all stakeholders |  |  |

# **VI. PROJECT SCALE-UP STRATEGY**

The implementation steps and tools described above provide the technical resources needed for project design, implementation, and monitoring and evaluation in an endemic zone.

The benchmarking approach suggested in an endemic country will consist of selecting as an implementation unit, a health region of which population could be around one million inhabitants for cost/effectiveness purposes.

In the region, a coordination unit composed of people disabled by NTDs or where applicable, people with disabilities with minimum project management skills will be established to lead the seven stages of implementation.

The strategy is made up of four (4) main pillars with strategic objectives (appendix related to project logframe) which can be adapted depending on the situation, context of the region and financial resources available. Here are the four pillars:

1. Advocacy and awareness-raising for improved access to physical, psychosocial, educational and economic rehabilitation services
2. Fighting stigma and promoting respect for the rights of persons with disabilities
3. Promoting mutual support between Pw/D-NTDs
4. Strengthening the organizational capacities of FAHCI to sustaining the actions of the Pw/D-NTDs.

The strategy is based on strengthening the capacities of Persons with Disabilities so that they can advocate to institutions responsible for inclusion or support for the physical, psychological and socio-economic rehabilitation of Pw/D-NTDs. Given that this approach has direct effects on improving quality of life in the medium and long term, it is important to mention in communication with the final beneficiaries the time necessary for these effects to be observed. It is encouraging to see that the support obtained is sustainable because it will be included by the targeted institutions in their objectives.

Also, it is necessary to allow a minimum of 3 years for the implementation of such strategies, keeping the above-mentioned reasons in mind.

# **CONCLUSION**

During the implementation of the project, we used numerous tools which we consider to be significant. Our toolkit is designed to be dynamic, scalable, and open to your suggestions for its continuous improvement. We happily share the tools we have found useful so far, while remaining open to innovations in project management.

# **BIBLIOGRAPHIC REFERENCES**

1. 17 Objectifs de développement durable - L'Agenda 2030 en France (agenda-2030.fr) 26/04/2024 à 11h45mn
2. Cartographie des parties prenantes - Search Images (bing.com)
3. CDDEP_Guide du dialogue avec les parties prenantes.pdf (ecologie.gouv.fr)
4. https://extranet.santemonteregie.qc.ca/app/uploads/2024/02/analyse-parties-prenantes.pdf
5. https://info.undp.org/docs/pdc/Documents/BFA/cheicklist%20cl%C3%B4ture%20PNVB.pdf
6. https://static.blog-projet.fr/wp-content/uploads/2017/05/23151101/Checklist-phase-de-cloture3.jpg
7. https://www.cairn.info/la-boite-a-outils-du-chef-de-projet--9782100848225.htm
8. https://www.theprojectgroup.com/blog/fr/gestion-des-parties-prenantes
9. La liste de contrôle de clôture de projet - Talents - EcommerceMag.fr
10. Les outils du chef de projet - Construisez votre propre boite à outils (manager-go.com), 26/04/2024 à 11h15mn.
11. Rapport d’évaluation finale du Projet Dignité
12. [Report A4 (humanrights.dk)](https://www.humanrights.dk/files/media/document/HRIA%20Toolbox_Stakeholder%20engagement_French.pdf)

#

# APPENDICES :

**PLAN D’ACTION DIGNITE :**

| **CODES** | **ACTIVITES** | **CIBLES** | **RESPONSABLE DE MISE EN ŒUVRE** | **CALENDRIER** |
| --- | --- | --- | --- | --- |
| **AXE 1** | **PAIDOYERS ET SENSIBILISATION POUR L'AMELIORATION DE L'ACCES AUX SERVICES DE REHABILITATION PHYSIQUE, PSYCHOSOCIAL EDUCATIF ET ECONOMIQUE** | | | |
| **OS 1.1** | **OBJECTIF SPECIFIQUE 1.1: FAIRE UN PLAIDOYER EN VUE D'ETENDRE LA GRATUITÉ DES SOINS AUX SERVICES DE RÉHABILITATION ET DE RÉADAPTATION DES PSH-MTN** | | | |
| **A1.1.1** | Organiser un atelier en vue d’élaborer et soumettre un projet d’arrêté pour la prise en charge gratuite des soins de réhabilitation et de réadaptation des PSH-MTN |  |  |  |
|  |  |  |  |  |
|  |  |  |  |  |
|  |  |  |  |  |
|  |  |  |  |  |
|  |  |  |  |  |
|  |  |  |  |  |
| **OS 1.2** | **OBJECTIF SPECIFIQUE 1.2 : RENFORCER LA PRISE EN CHARGE PSYCHOLOGIQUE DES PSH-MTN** | | | |
| **A1.2.1** | Organiser une réunion de plaidoyer ciblant les décideurs en vue d’améliorer la prise en charge psychologique des PSH-MTN |  |  |  |
|  |  |  |  |  |
| **A1.2.2** | Organiser une réunion de plaidoyer ciblant les autorités des districts sanitaires afin que la prise en charge psychologique soit intégrée aux soins des PSH-MTN |  |  |  |
| **OS.1.3** | **OBJECTIF SPECIFIQUE 1.3 : PROMOUVOIR LES CONNAISSANCES ET ATTITUDES QUI FAVORISENT LA RÉDUCTION DES INCAPACITÉS LIÉES AUX MTN** | | | |
| **A1.3.1** | Organiser une réunion de restitution des résultats de l’étude et sensibiliser sur les facteurs qui favorisent la survenue des incapacités et les conséquences socio-économiques des MTN auprès des autorités sanitaires du Gbeke |  |  |  |
| **A1.3.2** | Organiser une émission radio trimestrielle pour la sensibilisation sur les MTN, la reconnaissance des signes suspects, les complications possibles et prise en charge, de même que les lois et règlements qui protègent les personnes handicapées |  |  |  |
|  |  |  |  |  |
|  |  |  |  |  |
|  |  |  |  |  |
| **A1.3.3** | Diffuser des vidéos sur WhatsApp et Facebook sur la sensibilisation sur les MTN, la reconnaissance des signes suspects, les complications possibles et la prise en charge, de même que les lois et règlements qui protègent les personnes handicapées |  |  |  |
| **A1.3.4** | Organiser 5 réunions de plaidoyer auprès des leaders communautaires pour lever les barrières culturelles et ou améliorer la collaboration avec le système de santé pour la prise en charge des PSH-MTN |  |  |  |
| **A1.3.5** | Participer aux réunions de planification sur la lutte contre les MTN dans les districts sanitaires |  |  |  |
|  |  |  |  |  |
|  |  |  |  |  |
| **OS 1.4** | **OBJECTIF SPECIFIQUE 1.4 : OFFRIR DES PRISES EN CHARGE SCOLAIRES AUX ENFANTS EN SITUATION DE HANDICAP DU FAIT DES MTN (reformulation ci-dessous)  Mobiliser des ressources en vue de l'intégration des enfants en situation de handicap du fait des MTN dans le système éducatif à travers des plaidoyers et des prises en charge scolaire** | | | |
| **A1.4.1** | Identifier les enfants handicapés du fait des MTN ayant des besoins en scolarisation |  |  |  |
|  |  |  |  |  |
|  |  |  |  |  |
|  |  |  |  |  |
| **A1.4.2** | Organiser une réunion de plaidoyer auprès des responsables de l’éducation nationale pour l’admission des enfants qui sont dans le besoin dans les établissements scolaires |  |  |  |
| **A1.4.3** | Organiser une réunion de mobilisation de ressources financières avec les partenaires Financiers et agences gouvernementales d’aide pour obtenir des prises en charges pour les PSH-MTN |  |  |  |
|  |  |  |  |  |
|  |  |  |  |  |
|  |  |  |  |  |
|  |  |  |  |  |
|  |  |  |  |  |
|  |  |  |  |  |
| **OS 1.5** | **OBJECTIF SPECIFIQUE 1.5 : FAIRE LA PROMOTION DES STRUCTURES SPECIALISEES DANS L’EDUCATION DES PH AUPRES DES PSH-MTN** | | | |
| **A1.5.1** | Identifier les structures spécialisées existantes en Côte d’Ivoire |  |  |  |
| **A1.5.2** | Adresser des courriers de sensibilisation et de demande d’admission des PSH-MTN dans les structures identifiées. |  |  |  |
| **A1.5.3** | Organiser la sensibilisation des PSH en âge d'aller à l'école ou en formation sur l'existence des structures spécialisées de l'éducation et sur leurs conditions d'accès |  |  |  |
| **OS 1.6** | **OBJECTIF SPECIFIQUE 1.6 : AMELIORER L’ACCES A LA FORMATION DES ADULTES HANDICAPEES PAR LES MTN** | | | |
| **A1.6.1** | Produire et diffuser auprès des PSH-MTN des prospectus qui informent sur les possibilités d’alphabétisation et de formation professionnelle |  |  |  |
| **A1.6.2** | Organiser une réunion de sensibilisation des responsables du système éducatif sur l’application des lois relatives à l’accès à l’alphabétisation et à la formation professionnelle des personnes handicapées |  |  |  |
|  |  |  |  |  |
|  |  |  |  |  |
|  |  |  |  |  |
|  |  |  |  |  |
| **OS 1.7** | **OBJECTIF SPECIFIQUE 1.7. : RENFORCER LES CAPACITES FINANCIERES (AUTONOMISER) DES PSH-MTN ET DES PERSONNES AIDANTES A TRAVERS LES TRANSFERTS MONETAIRES PERIODIQUES, LE DEVELOPPEMENT DES AGR ET LA PROMOTION DE L’AUTO-EMPLOI** | | | |
| **A1.7.1** | Identifier des partenaires pour un appui financier et technique aux personnes en situation de handicap |  |  |  |
|  |  |  |  |  |
|  |  |  |  |  |
|  |  |  |  |  |
| **A1.7.2** | Etablir des conventions de collaboration avec deux institutions spécialisées dans la promotion de l’auto-emploi |  |  |  |
|  |  |  |  |  |
| **A1.7.3** | Mettre en place un fond pour garantir l’accès aux micro-crédits des PSH-MTN |  |  |  |
|  |  |  |  |  |
| **A1.7.4** | Recenser les besoins en AGR pour PSH-MTN |  |  |  |
| **A1.7.5** | Identifier les PSH-MTN devant bénéficier des transferts monétaires d’urgence |  |  |  |
| **A1.7.6** | Faire le plaidoyer auprès des institutions en charge des transferts monétaires pour la prise en compte des PSH-MTN |  |  |  |
| **A1.7.8** | Mettre en place un comité de suivi intersectoriel |  |  |  |
|  |  |  |  |  |
| **A1.7.9** | Organiser une rencontre de plaidoyer auprès de l’inspection du travail afin que les employeurs publics et privés appliquent les lois en faveur de l’emploi des PSH-MTN |  |  |  |
| **AXE 2** | **LUTTE CONTRE LA STIGMATISATION ET PROMOTION DU RESPECT DES DROITS DES PERSONNES HANDICAPEES** | | | |
| **OS 2.1** | **OBJECTIF SPECIFIQUE 2.1 : AMELIORER LES CONNAISSANCES DES POPULATIONS Y COMPRIS DES PSH-MTN ET LES AIDANTES SUR LES LOIS ET REGLEMENTATIONS PROTEGEANT LES PERSONNES HANDICAPEES.** | | | |
| **A2.1.1** | Organiser des séances de sensibilisation à travers des radios communautaires (intègre aux activités de sensibilisation) |  |  |  |
|  |  |  |  |  |
| **A2.1.2** | Produire et diffuser 500 affiches de sensibilisation sur les lois et réglementations protégeant les personnes handicapées, les structures spécialisées de prise en charge et les services de réhabilitation / réadaptation. |  |  |  |
|  |  |  |  |  |
|  |  |  |  |  |
|  |  |  |  |  |
|  |  |  |  |  |
| **OS 2.2** | **OBJECTIF SPECIFIQUE 2.2 : PROMOUVOIR LES DROITS DES PSH-MTN** | | | |
| **A2.2.1** | Développer et diffuser par le canal de WhatsApp auprès des PSH-MTN et de leur aidant une vidéo qui présente les droits des personnes handicapées. |  |  |  |
| **A2.2.2** | Organiser une réunion de plaidoyer auprès des autorités en charge de l’administration du territoire et des Directeurs Régionaux des ministères cibles pour sensibiliser sur les lois et règlement qui protègent les personnes handicapées |  |  |  |
| **A2.2.3** | Etablir un partenariat avec la clinique juridique de Bouaké pour la gestion des dénis de droits des PSH-MTN |  |  |  |
|  |  |  |  |  |
| **A2.2.4** | Organiser une réunion de plaidoyer auprès du ministère de la Justice a Bouaké pour obtenir son appui à la promotion des droits des PSH-MTN |  |  |  |
|  |  |  |  |  |
| **OS 2.3** | **OBJECTIF SPECIFIQUE 2.3 : RENFORCER LA LUTTE CONTRE LA STIGMATISATION ET LA DISCRIMINATION SOCIALE ET FAVORISER L'INCLUSION ET LA PARTICIPATION DES PSH-MTN** | | | |
| **A2.3.1** | Répertorier et diffuser les lois et règlements existants protégeant les PSH |  |  |  |
|  |  |  |  |  |
| **A2.3.2** | Répertorier les pratiques stigmatisantes expérimentées dans la zone du projet par les PSH-MTN |  |  |  |
| **A2.3.3** | Organiser 5 réunions de Sensibilisation des leaders communautaires à l’abandon par les membres de la communauté des pratiques stigmatisantes |  |  |  |
| **AXE 3** | **PROMOTION DE L’ENTRAIDE ENTRE PSH-MTN** | | | |
| **OS 3.1** | **OBJECTIF STRATEGIQUE 3.1. RENFORCER LES CAPACITES D'AUTO-ASSISTANCE DES PH-MTN** | | | |
| **A3.1.1** | Former un pool PSH-MTN/FAHCI et des aidants à la prise en charge psychologique des PSH-MTN |  |  |  |
|  |  |  |  |  |
| **A3.1.2** | Mettre en place une équipe d’assistance pour la constitution des dossiers d’admission et d’orientation des PSH-MTN vers les structures spécialisées |  |  |  |
| **A3.1.3** | Former un pool de 25 personnes sur les MTN pour la sensibilisation sur la reconnaissance des signes suspects, les complications possibles et la prise en charge, de même que les lois et règlement qui protègent les personnes handicapées |  |  |  |
| **A3.1.4** | Organiser une formation d’un pool de PSH-MTN à la promotion et à la défense des droits des PSH. |  |  |  |
| **A3.1.5** | Organiser une réunion de promotion des groupes d'entraide avec les PH-MTN et leurs aidants |  |  |  |
| **AXE 4** | **RENFORCEMENT DES CAPACITES ORGANISATIONNELLES DE L'ASSOCIATION FAHCI EN VUE DE LA PERENISATION DES ACTIONS DES PSH-MTN** | | | |
| **OS 4.1** | **OBJECTIF SPECIFIQUE 4.1 : RENFORCER LES CAPACITES DE GESTION ASSOCIATIVE ET DE MOBILISATION DE RESSOURCES POUR PERENISER LES ACTIONS DES PH-MTN** | | | |
| **A4.1.1** | Organiser des rencontres trimestrielles d’échange avec les PTF en vue de l'amélioration de la gestion administrative, financière et programmatique de l’association |  |  |  |
| **A4.1.2** | Établir un partenariat avec un cabinet pour le développement et la mise en œuvre d'un plan de mobilisation des ressources devant soutenir l’exécution du plan |  |  |  |
| **OS 4.2** | **OBJECTIF SPECIFIQUE 4.2 : ASSURER LA COORDINATION, LE SUIVI ET L’EVALUATION DU PROJET** | | | |
| **A4.2.1** | Elaborer et mettre en œuvre un plan de suivi et évaluation du projet |  |  |  |
| **A4.2.2** | Équiper la cellule de coordination en matériel informatique |  |  |  |
| **A4.2.3** | Organiser des réunions mensuelles de suivi du projet avec les parties prenantes |  |  |  |
|  |  |  |  |  |
|  | *PSH : Personnes en Situation de Handicap* |  |  |  |
|  | *MTN : Maladies Tropicales Négligées* |  |  |  |
|  | *FAHCI : Fédération des Associations des Handicapés de Côte d'Ivoire* |  |  |  |

**MATRICE SUIVI INDICATEURS :**

| **OBJECTIFS** | **LIBELLE DE L'INDICATEUR** | **Données de base (Baseline)** | | | **Données cibles** | | | | | **Sources de données / sources de vérification** |
| --- | --- | --- | --- | --- | --- | --- | --- | --- | --- | --- |
|  |  | **Valeur** | **Année** | **Source de données** | **juil-22** | **sept-22** | **dec-2022** | **mars-23** | **juin-23** |  |
| **Accroitre l'influence des personnes affectées par les MTN sur leur accès aux services de prise en charge en Côte d'Ivoire** | | | | | | | | | | |
| **AXES STRATEGIQUES (AS)** | **INDICATEURS D'IMPACTS INTERMEDIAIRES** |  |  |  |  |  |  |  |  |  |
| **AS1** : Faire des plaidoyers et sensibiliser des acteurs pour l'amélioration de l'accès des PSH-MTN aux services de réhabilitation physique, psychosocial, éducatif et économique | OS1. IOV1 : % de PSH-MTN éligibles bénéficiant des services de réhabilitation et réadaptation physique (chirurgie, prothèse, etc.) |  |  | Baseline du projet Dignité |  |  |  |  |  | Endline du projet Dignité |
|  | OS1. IOV2 : % de PSH-MTN bénéficiant des services de prise en charge psychosociale (écoute, conseils, suivi psychosocial) |  |  | Baseline du projet Dignité |  |  |  |  |  | Endline du projet Dignité |
|  | OS1. IOV3 : % de PSH-MTN éligibles bénéficiant des services d'appui à l'éducation (intégration dans des structures spécialisées, prise en charge scolaire, alphabétisation, formation professionnelle) |  |  | Baseline du projet Dignité |  |  |  |  |  | Endline du projet Dignité |
|  | OS1. IOV4 : % de PSH-MTN bénéficiant des services d'appui socioéconomique (transfert monétaires, AGR, etc.) |  |  | Baseline du projet Dignité |  |  |  |  |  | Endline du projet Dignité |
| **AS2** : Lutter contre la stigmatisation et promouvoir le respect des droits des Personnes en Situation de Handicap du fait des MTN | OS2. IOV1 : % de PSH-MTN victimes de stigmatisation vécue |  |  | Baseline du projet Dignité |  |  |  |  |  | Endline du projet Dignité |
|  | OS2. IOV2 : % de PSH-MTN victimes d'auto-stigmatisation |  |  | Baseline du projet Dignité |  |  |  |  |  | Endline du projet Dignité |
|  | OS2. IOV3 : % de PSH-MTN connaissant leurs droits fondamentaux, les lois et règlementations protégeant les PSH |  |  | Baseline du projet Dignité |  |  |  |  |  | Endline du projet Dignité |
| **AS3** : Promouvoir de l'entraide entre les Personnes en Situation de Handicap du fait des MTN | OS3. IOV1 : % de PSH-MTN ayant bénéficié d'entraide de la part de leurs pairs |  |  | Baseline du projet Dignité |  |  |  |  |  | Endline du projet Dignité |
| **AS4 :** Renforcer les capacités organisationnelles de la FAHCI en vue de pérenniser les actions des PSH-MTN | OS4. IOV1 : Capacité en gestion associative de la FAHCI en faveur des PSH-MTN |  |  | Baseline du projet Dignité |  |  |  |  |  | Endline du projet Dignité |
|  | OS4. IOV2 : Niveau de mobilisation de ressources par la FAHCI en faveur des PSH-MTN |  |  | Baseline du projet Dignité |  |  |  |  |  | Endline du projet Dignité |
|  | OS4. IOV3 : Niveau de coordination par la FAHCI des actions menées par les PSH-MTN |  |  | Baseline du projet Dignité |  |  |  |  |  | Endline du projet Dignité |
| **EFFETS / PRODUITS** | **INDICATEURS D'EFFETS IMMEDIATS / PRODUITS** |  |  |  |  |  |  |  |  |  |
| **OS1 : Faire des plaidoyers et sensibiliser des acteurs pour l'amélioration de l'accès des PSH-MTN aux services de réhabilitation physique, psychosocial, éducatif et économique** | | | | | | | | | | |
| Effet 1.1: La gratuité des soins est étendue aux services de réhabilitation et de réadaptation des PSH-MTN | OS1.R1. IOV1 : un arrêté ministériel est pris pour l'extension de la gratuité des services de réhabilitation et de réadaptation des PSH-MTN |  |  |  |  |  |  |  |  | Copie du projet d'arrêté |
|  | OS1.R1. IOV2 : # de PSH-MTN (hommes / femmes) ayant bénéficié gratuitement de soins aux services de réhabilitation et de réadaptation |  |  |  |  |  |  |  |  | Données de suivi du projet Dignité |
| Effet 1.2 : Les autorités sanitaires nationales et régionales ciblées sont engagées pour une amélioration de la prise en charge psychologique et son intégration dans les soins fournis aux PSH-MTN | OS1.R2. IOV1 : une note de service est prise par le Directeur Régional de la Santé (Gbeke) en vue de l'intégration de la prise en charge psychologique dans les services de soins aux PSH-MTN |  |  |  |  |  |  |  |  | Copie de la note de service |
|  | OS1.R2. IOV2 : # et % de PSH-MTN dans la région du Gbeke bénéficiant désormais de la prise en charge psychologique |  |  |  |  |  |  |  |  | Données de suivi du projet Dignité |
| Effet 1.3 : Une stratégie de sensibilisation est élaborée pour améliorer les connaissances et attitudes des populations sur les MTN (signes, symptômes, complications, Bonnes Pratiques) en vue de réduire les incapacités | OS1.R3. IOV1 : # d'émissions radios diffusées sur les MTN (signes, symptômes, complications, Bonnes Pratiques) |  |  |  |  |  |  |  |  | Données de suivi du projet Dignité |
|  | OS1.R3. IOV2 : # de réunions organisées avec les acteurs spécifiques (leaders communautaires, districts sanitaires, malades de MTN) sur les résultats de l'étude, les symptômes / complications des MTN, les Bonnes Pratiques en matière de MTN, collaboration avec le système de santé et la planification des actions de lutte contre les MTN |  |  |  |  |  |  |  |  | Comptes rendus de réunions / rencontres |
|  | OS1.R3. IOV3 : # personnes sensibilisées (hommes/femmes) à travers les réunions, les séances communautaires et les réseaux sociaux |  |  |  |  |  |  |  |  | Données de suivi du projet Dignité |
| Effet 1.4 : Les acteurs sont engagés, des ressources sont mobilisées et des dispositions sont prises pour l'intégration des enfants en situation de handicap du fait des MTN dans le système scolaire | OS1.R4. IOV1 : # de réunions de plaidoyer organisées avec les autorités éducatives et les partenaires techniques et financiers en vue de l'intégration des enfants PSH-MTN dans le système éducatif |  |  |  |  |  |  |  |  | Comptes rendus de réunions / rencontres |
|  | OS1.R4. IOV2 : # et % d'enfants (garçons / filles) en situation de handicap du fait des MTN recensés ayant effectivement reçu une prise en charge scolaire ou ayant été intégrés dans le système éducatif |  |  |  |  |  |  |  |  | Données de suivi du projet Dignité |
| Effet 1.5 : Les PSH-MTN éligibles connaissent les structures spécialisées dans l'éducation des PSH et les conditions pour y accéder | OS1.R5. IOV1 : # de structures spécialisées dans l'éducation des PSH existantes en Côte d'Ivoire et dans la région du Gbeke |  |  |  |  |  |  |  |  | Données de suivi du projet Dignité |
|  | OS1.R5. IOV2 : # de PSH éligibles (garçons / filles) sensibilisées sur les structures spécialisées dans l'éducation des PSH et sur leurs conditions d'admission |  |  |  |  |  |  |  |  | Données de suivi du projet Dignité |
| Effet 1.6 : Les acteurs sont engagés, des ressources sont mobilisées et l'accès des adultes, en situation de handicap du fait des MTN à des formations, est facilité et amélioré | OS1.R6. IOV1 : # de rencontres / réunions de plaidoyers organisées avec les acteurs ciblés pour la formation des adultes PSH-MTN |  |  |  |  |  |  |  |  | Comptes rendus de réunions / rencontres |
|  | OS1.R6. IOV2 : # d'adultes (hommes/femmes) PSH-MTN sensibilisées sur les opportunités d'éducation ou de formation professionnelle disponibles pour les PSH |  |  |  |  |  |  |  |  | Données de suivi du projet Dignité |
|  | OS1.R6. IOV3 : # d'adultes (hommes/femmes) PSH-MTN ayant bénéficié ou bénéficiant de services d'éducation /formation disponibles pour les adultes PSH |  |  |  |  |  |  |  |  | Données de suivi du projet Dignité |
| Effet 1.7 : Les acteurs sont engagés, des ressources sont mobilisées et les capacités économiques et financières (autonomisation) des PSH-MTN sont renforcées à travers des transferts monétaires, de développement des AGR et de l'auto-emploi | OS1.R7. IOV1 : # d'actions de plaidoyers (identification des partenaires, réunion, processus de conventions, Comité de suivi, visites de sites) organisées en direction des acteurs ciblés pour le renforcement des capacités économiques et financières des PSH-MTN |  |  |  |  |  |  |  |  | Données de suivi du projet Dignité / CR de réunions |
|  | OS1.R7. IOV2 : un fonds de garantie des micro-crédits à l'endroit des PSH-MTN d'un montant de x F CFA est mis en place et fonctionnel |  |  |  |  |  |  |  |  | Document confirmant la disponibilité du fonds de garantie |
|  | OS1.R7. IOV3 : # de conventions de partenariats signés avec des structures spécialisées dans l'auto-emploi en vue de l'encadrement des PSH-MTN |  |  |  |  |  |  |  |  | Copie des conventions signées |
|  | OS1.R7. IOV4 : # et % de PSH-MTN (hommes / femmes) recensés et ayant bénéficié de transferts monétaires et montant de ces transferts |  |  |  |  |  |  |  |  | Données de suivi du projet Dignité |
|  | OS1.R7. IOV5 : # et % de PSH-MTN (hommes / femmes) recensés et ayant bénéficié de d'AGR et montant de ces AGR |  |  |  |  |  |  |  |  | Données de suivi du projet Dignité |
| **OS2 : Lutter contre la stigmatisation et promouvoir le respect des droits des Personnes en Situation de Handicap du fait des MTN** |  |  |  |  |  |  |  |  |  |  |
| Effet 2.1 : Les connaissances des populations y compris des PSH-MTN et des personnes aidantes sur les lois et règlementations protégeant les PSH sont améliorées | OS2.R1. IOV1 : # de PSH-MTN (hommes / femmes) ou personnes aidantes sensibilisées sur les lois et règlementations protégeant les PSH |  |  |  |  |  |  |  |  | Données de suivi du projet Dignité |
|  | OS2.R1. IOV2 : # d'acteurs publics ciblés et de professionnels de la santé, sensibilisés sur les lois et règlementations protégeant les PSH |  |  |  |  |  |  |  |  | Données de suivi du projet Dignité |
|  | OS2.R1. IOV3 : Niveau de connaissances des acteurs publics et des professionnels de la santé, sur les lois et réglementation protégeant les PSH |  |  |  |  |  |  |  |  | Entretiens à mi-parcours / Endline du projet |
| Effet 2.2 : Les droits fondamentaux des PSH-MTN sont connus et vulgarisés | OS2.R2. IOV1 : # de PSH-MTN (hommes / femmes) ou personnes aidantes sensibilisées sur leurs droits fondamentaux |  |  |  |  |  |  |  |  | Données de suivi du projet Dignité |
|  | OS2.R2. IOV2 : # d'acteurs publics ciblés et de professionnels de la santé, sensibilisés sur les droits fondamentaux des PSH-MTN |  |  |  |  |  |  |  |  | Données de suivi du projet Dignité |
|  | OS2.R2. IOV3 : Niveau de connaissances des acteurs publics et des professionnels de la santé, sur les droits fondamentaux des PSH-MTN |  |  |  |  |  |  |  |  | Entretiens à mi-parcours / Endline du projet |
|  | OS2.R2. IOV4 : # et % de cas de violation des droits des PSH-MTN et de leurs proches gérés avec succès par les services de cliniques juridiques |  |  |  |  |  |  |  |  | Données de suivi du projet Dignité |
| Effet 2.3 : La stigmatisation et la discrimination sociale envers les PSH-MTN sont réduites et leur participation / inclusion sociale est améliorée | OS2.R3. IOV1 : # d'actions de lutte contre la stigmatisation et la discrimination envers les PSH-MTN promues à travers les réunions de plaidoyers, les séances de sensibilisation communautaires et les radios communautaires |  |  |  |  |  |  |  |  | Données de suivi du projet Dignité |
|  | OS2.R3. IOV2 : # et % d'acteurs ciblés (leaders communautaires, agents de santé, etc) engagés dans la lutte contre la stigmatisation et la discrimination envers les PSH-MTN |  |  |  |  |  |  |  |  | Données de suivi du projet Dignité / Preuves d'engagement |
| **OS3 : Promouvoir de l'entraide entre les Personnes en Situation de Handicap du fait des MTN** |  |  |  |  |  |  |  |  |  |  |
| Effet 3.1 : Les capacités d'auto-assistance et d'entraide des PSH-MTN se sont renforcées | OS3.R1. IOV1 : # et % d'actions ciblées de renforcement de capacités des PSH réalisées en vue de l'entraide et de l'auto-assistance entre PSH-MTN |  |  |  |  |  |  |  |  | Rapports de formation |
|  | OS3.R1. IOV2 : # de PSH-MTN assistées par leurs pairs formés (prise en charge psychologique, orientation en vue d'une formation, aide à l'emploi, aide à la mise en place d'AGR, défense des droits, etc.) |  |  |  |  |  |  |  |  | Données de suivi du projet Dignité |
|  | OS3.R1. IOV3 : # d'associations de PSH-MTN mises en place à la suite des activités de renforcement des capacités pour l'entraide et l'auto-assistance |  |  |  |  |  |  |  |  | Données de suivi du projet Dignité |
| **OS4 : Renforcer les capacités organisationnelles de la FAHCI en vue de pérenniser les actions des PSH-MTN** |  |  |  |  |  |  |  |  |  |  |
| Effet 4.1 : Les capacités de la FACI en gestion associative et en mobilisation de ressources en faveur des PSH-MTN sont renforcées | OS4.R1. IOV1 : # de rencontres d'échanges organisées avec les PTF en vue du renforcement des capacités (gestion associative, programmatique, mobilisation des ressources) de la FAHCI |  |  |  |  |  |  |  |  | Comptes rendus de réunions / rencontres |
|  | OS4.R1. IOV2 : Un plan de mobilisation de ressources additionnelles en faveur des PSH-MTN est élaboré et fonctionnel |  |  |  |  |  |  |  |  | Copie du plan validé |
|  | OS4.R1. IOV3 : Niveau des ressources additionnelles mobilisées par la FAHCI en faveur des PSH-MTN |  |  |  |  |  |  |  |  | Données de suivi du projet Dignité / Endline du projet |
| Effet 4.2 : Le Projet Dignité est coordonné, suivie et évaluée | OS4.R2. IOV1 : Niveau de fonctionnalité du plan de S&E / Apprentissage du projet Dignité |  |  |  |  |  |  |  |  | Plan de S&E disponible |
|  | OS4.R2. IOV2 : Niveau d'équipement de la cellule de coordination de la FAHCI en matériel |  |  |  |  |  |  |  |  | Preuves d'équipement |
|  | OS4.R2. IOV3 : # et % de rencontres périodiques de suivi et d'apprentissage dans le cadre du projet Dignité |  |  |  |  |  |  |  |  | Comptes rendus de réunions / rencontres |
|  | OS4.R2. IOV4 : # de rapports de suivi évaluation du projet élaborés et partagés |  |  |  |  |  |  |  |  | Copie de rapport de S&E |

**THEORIE DU CHANGEMENT :**

**OUTILS S&E :**

| **N°** | **Outils de S&E** |
| --- | --- |
| 1 | Cadre Logique (à finaliser pour les 3 autres axes) |
| 2 | Matrice de suivi de la performance du projet (sur la base Cadre Logique défini) |
| 3 | Diagramme de la Théorie du Changement du projet |
| 4 | Fiches de suivi des indicateurs de performance du projet |
| 5 | Outil qualitatif d'apprentissage (retours de terrain : points de satisfaction/ insatisfaction, leçons apprises, succès, échecs, etc.) |
| 6 | Canevas de rapports trimestriels |
| 7 | Outil opérationnel et dynamique (MS Excel web) de suivi des indicateurs de performance du projet |

1. [17 Sustainable Development Goals - Agenda 2030 in France (agenda-2030.fr)](https://www.agenda-2030.fr/17-objectifs-de-developpement-durable/) 04/26/2024 at 11:45 a.m. [↑](#footnote-ref-1)
2. Framework for the fight against skin NTDs [↑](#footnote-ref-2)
