# Supplementary material for: People-centered strategies to mobilize people living with disabilities due to Neglected Tropical Diseases (PD-NTDs) to influence policy and programs: A mixed-methods study in Côte d’Ivoire
Source: PLoS Negl Trop Dis. 2025 Sep 8;19(9):e0013485. doi: 10.1371/journal.pntd.0013485 (PMC12431663; doi:10.1371/journal.pntd.0013485)
Supplement: S1 Data — (DOCX) [file pntd.0013485.s005.docx]

**REPUBLIC OF COTE D’IVOIRE**

UNION – DISCIPLINE – WORK

**Study report**

**mid-term evaluation**

Mid-term evaluation of the project to identify a strategy to increase the influence of people affected by NTDs on access to care services for people affected by NTDs in Côte d'Ivoire (Dignité project)

**April 4, 2023**

| **Presented by:** | **CESI** (Statistical and Computer Science Studies Firm)  Tel: (+225) 27 21 38 18 02  Mobile: (+225) 07 08 27 37 38  **Email:** [cabstat_info@yahoo.fr](mailto:cabstat_info@yahoo.fr) / infos@cesi-ci.com |
| --- | --- |

**“Mid-term evaluation of the Dignity Project in Côte d’Ivoire »**

This project aims to explore effective strategies and mechanisms for mobilizing people affected by NTDs and their caregivers to influence government policies and multi-sectoral programs to improve and integrate the support system for people disabled by NTDs in Côte d'Ivoire.

| ***Search site:*** | Gbêkê Health Region in Ivory Coast |
| --- | --- |
| ***Type of study:*** | Qualitative study |
| ***Provider :*** | CESI (Statistical and Computer Science Studies Firm) |
| ***Study Team:*** | - LOROU Bi Gohoré Jean-Maxime, Principal Consultant, Statistical Engineer, specialist in sampling, processing and statistical analysis of data - Dr N'DRI Kouadio Patrice, Sociologist, Consultant, Research Professor, Assistant Professor at Alassane Ouattara University in Bouaké - TANOH Armand Hira, Consultant, Statistician, Specialist in Project Monitoring and Evaluation, Director of CESI; |
| ***Study period:*** | March – April 2023 |
| ***Study Sponsor:*** | COPTIMENT (Business Growth and Optimization) Ivory Coast |
| ***Members of the***  ***scientific committee*** | - Dr. Assie Nda Kouassi Marcellin, Director of the National Leprosy Elimination Program; - Dr Julien Aké, Associate Director of Coptiment , Doctor, Specialist in Community Health, Expert in the fight against Neglected Tropical Diseases (NTDs), - Dr. Koffi Aboa, Representative of the PNLUB - Representative of the PNLMTN-CP - Prof. Isaac TIEMBRE, Associate Researcher, Researcher in Public Health and Specialist in Research Methodology, Felix Houphouët Boigny University, Consultant at Coptiment - Dr Boko Nadège Koidia , Associate Researcher, Researcher in Social Sciences, Felix Houphouët Boigny University, Consultant at Coptiment - Ms. Konan, Representative of the Federation of Associations of the Disabled of Ivory Coast - Sara Marshall, Program Director, Effect : Hope Canada |

**Table of Contents**

[List of abbreviations or acronyms](#_Toc134792148)  [1](#_Toc134792148)

[Summary](#_Toc134792149)  [2](#_Toc134792149)

[I- Introduction](#_Toc134792150)  [1](#_Toc134792150)

[I-1 Presentation of the context of project](#_Toc134792151)  [1](#_Toc134792151)

[I-2 Scientific justification/rationale for the study:](#_Toc134792152)  [1](#_Toc134792152)

[I-3 Implementation](#_Toc134792153)  [2](#_Toc134792153)

[II- Objectives of the mid-term evaluation](#_Toc134792154)  [2](#_Toc134792154)

[III- Methodology](#_Toc134792155)  [2](#_Toc134792155)

[III-1 Method and tools](#_Toc134792156)  [2](#_Toc134792156)

[*III-1-1 The documentary review*](#_Toc134792157)  [2](#_Toc134792157)

[*III-1-2 The qualitative survey*](#_Toc134792158)  [3](#_Toc134792158)

[*III-1-3 Evaluation area and targets*](#_Toc134792159)  [4](#_Toc134792159)

[III-2 Data collection](#_Toc134792160)  [4](#_Toc134792160)

[III-3 Data processing and report writing](#_Toc134792161)  [5](#_Toc134792161)

[IV- Results of the mid-term evaluation](#_Toc134792162)  [6](#_Toc134792162)

[IV-1 Level of execution of activities](#_Toc134792163)  [6](#_Toc134792163)

[IV-2 Analysis of the relevance of the project (vs. global and specific environment)](#_Toc134792164)  [12](#_Toc134792164)

[IV-3 Analysis of the coherence of the project](#_Toc134792165)  [13](#_Toc134792165)

[IV-4 Analysis of project effectiveness (planning and execution)](#_Toc134792166)  [14](#_Toc134792166)

[IV-5 Analysis of the strengths and weaknesses of the project](#_Toc134792167)  [15](#_Toc134792167)

[IV-6 Analysis of the potential sustainability of project actions](#_Toc134792168)  [18](#_Toc134792168)

[IV-7 Lessons learned and good practices](#_Toc134792169)  [19](#_Toc134792169)

[Conclusions and recommendations for successful achievement of results for the remaining period](#_Toc134792170)  [20](#_Toc134792170)

[Bibliography](#_Toc134792171)  [21](#_Toc134792171)

[Annexes](#_Toc134792172)  [22](#_Toc134792172)

#

# List of abbreviations or acronyms

| AMM/ AMD | : | Mass Drug Administration |
| --- | --- | --- |
| BIT | : | International Labour Office |
| KAP | : | Knowledge Attitudes and Practices |
| CESI SARL | : | Statistical and IT Research Firm, limited liability company |
| CI | : | Ivory Coast |
| COVID 19 | : | Coronavirus disease 19 |
| DSC | : | Community Health Department |
| FL | : | Lymphatic filariasis |
| FAHCI | : | Federation of Associations for the Promotion of People with Disabilities in Côte d'Ivoire |
| LRI | : | Leprosy Research Initiative |
| MC | : | Skin Manifestation |
| MTN | : | Neglected Tropical Disease |
| ODK | : | Open Data Kit |
| NGO | : | Non-Governmental Organization |
| PNDS |  | National Health Development Plan (NHDP) |
| PNEL | : | National Leprosy Elimination Program |
| PNLMTN CP | : | National Program for the Fight against Neglected Tropical Diseases with Preventive Chemotherapy |
| PNLUB | : | National Program to Combat Buruli Ulcer |
| THA |  | Human African Trypanosomiasis |
| HIV | : | Human immunodeficiency virus |

# Summary

As an endemic country, Côte d'Ivoire is a victim of 10 NTDs, including leprosy and Buruli ulcer, which are most prevalent in the Gbêkê health region. The study report on the identification of the specific needs of people with disabilities due to Neglected Tropical Diseases (NTD-PSH) in Côte d'Ivoire, conducted in 2022, provides a general overview of the situation of victims. This situation, which is not encouraging, has led to the establishment of a pilot project called the "dignity project."

Indeed, the dignity project aims to carry out activities towards PSH-MTN grouped under four (04) main axes which are:

**Axis 1** : Advocacy and awareness raising for improving access to physical, psychosocial, educational and economic rehabilitation services,

**Axis 2** : Fight against stigmatization and promotion of respect for the rights of people with disabilities,

**Axis 3** : Promoting mutual aid between PSH-MTN

**Axis 4** : Strengthening the organizational capacities of the FAHCI association with a view to the sustainability of PSH-MTN actions.

This mid-term evaluation aims to understand the evolution of the implementation of activities in order to measure the effectiveness of the project on the experience of the beneficiaries.

This study was based on a qualitative approach using the following data collection tools: documentary review, individual semi-structured interviews and group interviews.

This assessment shows that the activities are completed at a rate of 62%. This proportion, appreciated by each stakeholder, has aroused the expression of their satisfaction. However, some difficulties were mentioned. They relate to the very limited volume of the budget which does not allow to fully meet the expectations of the members, and the problem of mobility since the implementation team does not have any machinery to better crisscross the localities under its supervision. As a result, the achievement of the project objectives seems problematic. To overcome these weaknesses with a view to preserving the achievements, the following recommendations were made. These are:

1. Increase the overall budget allocated to the execution of the project in order to make the implementation team more operational,
2. Provide the project team with sufficient means of mobility (vehicles, motorcycles, etc.) and fuel vouchers for its travel in the various districts and specific localities,
3. Provide the project team with additional equipment to strengthen it,
4. Review the functioning of the scientific committee in order to improve its collaboration with the other stakeholders in the project while prioritizing the sustainability of the achievements,
5. Provide the team with a monthly volunteer support fund,
6. Encourage and motivate support partners to act for the well-being of PSH-MTN,
7. Begin supporting PSH-MTNs by using their respective associations as support,
8. Include in the project implementation process the visit of each program to the target to better assess the achievement of the objectives,
9. Raise awareness among the FAHCI so that it takes ownership of the project by perpetuating the achievements for its extension throughout the Ivorian territory.

# I- Introduction

## I-1 Presentation of the project context

Disability is historically linked to occult beliefs in Côte d'Ivoire. It remains a source of stigma and significant exclusion from access to education, the labor market, and healthcare. Several initiatives aim to improve the daily lives of people with disabilities, but systematic discrimination persists. Although some policies and regulations have been developed to support the situation of people with disabilities in the country, this situation is not taken into account in the lives and priorities of people living with disabilities [DIDR-OFPRA Report: People with Disabilities - Côte d'Ivoire 2018].

Côte d'Ivoire, a sub-Saharan African country, is primarily affected by multiple NTDs. The National Health Development Plan (NHDP) 2016-2020 indicates that the country is endemic for 10 NTDs, namely onchocerciasis, lymphatic filariasis (LF), soil-transmitted helminthiasis (ST), trachoma, schistosomiasis, leprosy, Buruli ulcer, yaws, human African trypanosomiasis (HAT), and finally Guinea worm, which has been declared eradicated.

Current Mass Drug Administration (MDA) programs for onchocerciasis, LF, soil-transmitted helminthiasis, and schistosomiasis do not include a significant morbidity management and disability prevention (MMDP) component alongside chemoprophylaxis. Unfortunately, individuals affected by NTDs discovered during MDAs do not have access to case management services and are thus at risk of developing disabilities.

For NTDs categorized as case-managed NTDs such as leprosy, Buruli ulcer, and yaws, the program continues to report a significant number of cases with disabilities already developed in the late stages of the disease. For leprosy, over the past three years, the program has reported approximately 20% of new cases with grade 2 disabilities [Annual Report 2019]. For Buruli ulcer, 26% of cases were reported with category 3 lesions that resulted in permanent disabilities [Annual Report 2018].

The exact number of people disabled by these diseases is not properly documented. Mapping of active cases is still ongoing.

## I-2 Scientific justification/rationale for the study:

The above analysis gives an idea that a good number of people affected by NTDs live with permanent disabilities. The health system alone cannot manage the socio-economic consequences on the person affected by the disease. The reason is that this requires a very strong coordination mechanism involving several sectors of human development.

Although there are a wide variety of studies on the psychosocial and economic reintegration of people with disabilities in general in various fields ( Batty 2015), in the case of NTDs and Buruli ulcer, certain personal and psychosocial determinants and factors have been little studied or remain little taken into account, and health programs have difficulty reaching the poorest populations ( Ndongo et al., 2014).

In this regard, Valérie Simonet (2014) identified in her study five main obstacles encountered by people with disabilities in terms of healthcare. These are the lack of financial resources, inadequate responses from healthcare structures, the lack of rehabilitation structures and skills, the poor organization of people with disabilities and the lack of information and awareness.

Hypothesizing that engaging people with disabilities through NTDs and organizing people with disabilities will sustainably improve the effectiveness and responsiveness of the Côte d'Ivoire government and programs to the needs of people with disabilities through NTDs.

The project aims to explore effective strategies and mechanisms for mobilizing people affected by NTDs that cause disabilities and their caregivers to influence government policies and multi-sectoral programs to improve and integrate the support system for people disabled by NTDs in Côte d'Ivoire.

## I-3 Implementation

The implementation of the project effectively began in October 2021 with the baseline assessment, followed by the development of the strategy focused on people with disabilities by NTDs to increase their influence on access to care services. The said strategy is in implementation in the Gbêkê health region.

This report describes the objectives, methodology and results of the mid-term evaluation of the project.

# II- Objectives of the mid-term evaluation

**General Objective** :

Evaluate the effectiveness of project implementation.

**Specific Objectives** :

1. Analyze the relevance and consistency of the project strategy
2. Measure effectiveness in planning and executing project activities
3. Identify the strengths and weaknesses of the project management bodies
4. Identify strengths and weaknesses in project implementation

# III- Methodology

## III-1 Method and tools

This evaluation study is essentially based on a qualitative and participatory approach. It used data collection tools such as documentary review, semi-structured and group interviews.

### *III-1-1 The documentary review*

This involved the collection and consultation of project documents and other documents necessary for this evaluation. In terms of the documents collected, we have:

- baseline protocol
- The final baseline report
- The project databases
- The logical framework and the action plan
- National frameworks and plans for combating NTDs
- Activity reports
- Minutes of meetings and gatherings
- Any project document deemed necessary

It also involved the verification of documents used for the implementation of the project and evidence of meetings such as attendance lists, awareness sheets, minutes and other leaflets.

Subsequently, a review of these different categories of documents was carried out for a good understanding of the level of implementation of the project in order to draw specific analyses.

### *III-1-2 The qualitative survey*

This survey used two main techniques for data collection. These are semi-structured interviews and focus groups. Thus, different interviews were conducted with the project stakeholders to gather their opinions, contributions and suggestions on the implementation of the project as well as the strengths and weaknesses to measure the effectiveness of the project implementation. The said stakeholders are:

- Scientific Committee
- Coptiment
- Federation of Associations for the Promotion of People with Disabilities in Côte d'Ivoire ( *FAHCI* )/project coordinator
- Gbêkê Region and Health Districts
- NTD focal points of the Gbêkê health districts
- Bouaké Legal Clinic
- National Leprosy Elimination Program (PNEL)
- National Program to Combat Buruli Ulcer (PNLUB)
- National Program for the Fight against Neglected Tropical Diseases with Preventive Chemotherapy (PNLMTN-CP)
- PSH and caregivers
- Other Ministries involved in the project

In terms of data collection tools, individual and group interview guides based on the activities included in the action plan were used. These guides were sent to project implementation stakeholders, institutional stakeholders, stakeholders involved in the care of people with disabilities due to NTDs and to people with disabilities themselves. This collection also aimed to gather relevant information on the implementation of the project, the difficulties encountered, and the strengths and weaknesses of the project management bodies.

It should also be noted that this study saw the involvement and effective participation of the firm's research team and the project's scientific committee. This arrangement facilitated access to the target structures and the provision of the necessary authorizations. To this end, the scientific committee was regularly informed of the progress of the study while validating the various stages of the mid-term evaluation.

### *III-1-3 Evaluation area and targets*

The area covered by the mid-term evaluation is the Gbêkê region (with Bouaké as its capital) and the target audience is all the project stakeholders, including the beneficiaries:

- Scientific Committee
- Coptiment
- Federation of Associations for the Promotion of People with Disabilities in Côte d'Ivoire ( *FAHCI* )/project coordinator
- Gbêkê Region and Health Districts
- NTD focal points of the Gbêkê health districts
- Bouaké Legal Clinic
- National Leprosy Elimination Program (PNEL)
- National Program to Combat Buruli Ulcer (PNLUB)
- National Program for the Fight against Neglected Tropical Diseases with Preventive Chemotherapy (PNLMTN-CP)
- PSH and caregivers
- Other Ministries involved in the project

## III-2 Data collection

The data collection mission was carried out in March 2023 in the departments of the stakeholders involved in the implementation of the project, both present and absent from Bouaké (for more details, see the list above). For residents, the meetings were in person, while non-residents were interviewed online, either by Zoom or by telephone.

The collection was carried out from Friday, March 10 to Thursday, March 16, a period of one week by three (03) investigators. The investigators were responsible for collecting the data according to the instructions given and ethical considerations, namely informed consent, respect for anonymity and confidentiality. This work was carried out under the supervision and direction of consultants from the CESI firm.

## III-3 Data processing and report writing

Some interviews were recorded using a Dictaphone and transcribed into Word software. Other interviews were conducted using an interview guide over the phone or face-to-face and recorded in notepads. The various interviews were analyzed through data immersion.

Overall, the data exploitation was carried out in the form of a content analysis of the speech delivered by the participants during the interviews, with illustration of the key messages by one or two verbatim which effectively reflect the perception expressed by the participants. This content analysis reinforced by a thematic analysis aimed at the production of the study report which reflects the reality of the evolution of the implementation of the pilot project in Gbêkê.

In the second phase, which is the drafting of the report, this was the work of the team of consultants. This report, once completed, will be submitted to the client. The latter, after observations and recommendations, will give the team of consultants the opportunity to proceed with their integrations in order to perfect the final version of the said report.

With regard to the level of execution and the proposals for execution, we note that the level of completion of the activities was assessed as follows:

- To calculate the overall proportion of activity execution, we calculated the sum of activities completed or in progress divided by the total number of activities planned in the logical framework. Thus, we obtained a rate of 62%. We used the same procedure to obtain the different proportions of activity execution levels for each axis.
- Our rating scale is presented in the table below.

**Table No. 1** : Scale for assessing the level of execution of project activities

| **No.** | **Proportion slice** | **Level of appreciation** | **Level of satisfaction** |
| --- | --- | --- | --- |
|  | 0% | Activity not yet executed | Not Applicable (NA) |
|  | From 1 to 10% | Activity in progress | Not satisfied |
|  | From 11 to 30% | Activity in progress | Not satisfied |
|  | From 31 to 50% | Activity performed on average | Moderately satisfied |
|  | From 51 to 70% | Activity sufficiently performed | Satisfied |
|  | From 71 90% | Activity fairly executed | Very satisfied |
|  | From 91 to 100% | Activity performed | Very very satisfied |

**Source** : our methodology

# IV- Results of the mid-term evaluation

## IV-1 Level of execution of activities

| **ACTIVITY INDICATORS** | **EXECUTION LEVEL** | **PROPORTION OF EXECUTION** | **OBSERVATIONS** |
| --- | --- | --- | --- |
| **ADVOCACY AND AWARENESS RAISING FOR IMPROVED ACCESS TO PHYSICAL, PSYCHOSOCIAL, EDUCATIONAL AND ECONOMIC REHABILITATION SERVICES** | | | |
| **COMMITMENT OF ADMINISTRATIVE AUTHORITIES AND COMMUNITY LEADERS TO THE IMPLEMENTATION OF THE STRATEGY** | | | |
| Organize a project launch ceremony to raise awareness among administrative authorities and community and association leaders in Bouaké | Made | 100% | Nothing to report |
| Obtaining stakeholder commitment to improving the quality of life of PH-MTN | Made | 100% | Nothing to report |
| Organize at least one meeting with each institution to strengthen collaboration | Made | 100% | Nothing to report |
| **ADVOCACY FOR FREE CARE INCLUDING REHABILITATION AND REHABILITATION CARE FOR PSH-MTN** | | | |
| Submit a request to the Ministry of Health and the Ministry of Social Protection to obtain free coverage for rehabilitation and readaptation care for PSH-MTN | Made | 100% | Nothing to report |
| Follow up on correspondence through at least one meeting with the authorities of the target institutions (Health and social protection). | Made | 100% | Nothing to report |
| **STRENGTHENING PSYCHOLOGICAL CARE FOR PSH-MTN** | | | |
| Organize an advocacy meeting targeting health district authorities so that psychological care is integrated into the care of PSH-MTNs | Not yet completed | 0% | Not at that level yet |
| Follow up with the PNEL for the training of health workers for this purpose | Not yet completed | 0% | Not at that level yet |
| Participate in planning meetings on NTD control in health districts and at central level | Made | 100% | Nothing to report |
| **IMPROVE THE KNOWLEDGE AND ATTITUDES OF POPULATIONS, PWDs AND CAREGIVERS ON NTDs AND LAWS AND REGULATIONS PROTECTING PWDs.** | | | |
| Organize a quarterly radio program to raise awareness about NTDs, recognition of suspicious signs, possible complications and management, as well as laws and regulations that protect people with disabilities. | It's on track: two radio shows per week instead of one show per quarter | 100% | Nothing to report |
| Distribute videos on WhatsApp and Facebook on raising awareness about NTDs, recognizing suspicious signs, possible complications and management, as well as laws and regulations that protect people with disabilities | Not yet completed | 0% | Not at this level yet, collecting WhatsApp numbers |
| Organize advocacy meetings with community leaders to remove cultural barriers and/or improve collaboration with the health system for the management of PSH-MTNs | Not yet completed | 0% | Radio awareness no meeting yet |
| Participate in planning meetings on NTD control in health districts | Made | 100% | Nothing to report |
| **MOBILIZING RESOURCES FOR THE INTEGRATION OF CHILDREN WITH DISABILITIES DUE TO NTDs INTO THE EDUCATION SYSTEM THROUGH ADVOCACY AND SCHOOL SUPPORT** | | | |
| Identify children with disabilities due to NTDs who have schooling needs during the PSH-NTD census with the help of focal points | Made | 100% | Nothing to report |
| Organize an advocacy meeting with national education officials for the admission of children in need into schools | Not yet completed | 0% | Not at that level yet |
| Organize a meeting to mobilize financial resources with financial partners and government aid agencies to obtain support for PSH-MTN. | Not yet completed | 0% | Expected in May 2023 |
| **THE PROMOTION OF SPECIALIZED STRUCTURES IN THE EDUCATION OF PH TO PSH-MTN** | | | |
| Identify existing specialized structures in Ivory Coast | Partially completed | 25% | Process in progress |
| Send awareness-raising letters and requests for admission of PSH-MTNs to the identified structures. | Not yet completed | 0% | Specialized structures not yet fully identified |
| Organize awareness-raising among PSH of school-age or training age on the existence of specialized education structures and the conditions of access to them | Made | 100% | Nothing to report |
| Have a PH-MTN WhatsApp address book for sharing information | Made | 100% | Nothing to report |
| **IMPROVING ACCESS TO TRAINING FOR ADULTS WITH DISABILITIES FROM NTDs** | | | |
| Produce and distribute leaflets (in digital and physical form) to PSH-MTNs providing information on literacy and vocational training opportunities. | realized | 100% | Nothing to report |
| Organize an awareness-raising meeting for education system officials on the application of laws relating to access to literacy and vocational training for people with disabilities | Not yet completed | 0% | Not at that level yet |
| **STRENGTHENING THE FINANCIAL CAPACITY (EMPLOYMENT) OF PSH-MTN AND CAREGIVERS THROUGH PERIODIC CASH TRANSFERS, THE DEVELOPMENT OF AGR AND THE PROMOTION OF SELF-EMPLOYMENT** | | | |
| Identify partners for financial and technical support for people with disabilities | Made | 100% | Nothing to report |
| Establish collaboration agreements with two institutions specializing in the promotion of self-employment | Made | 100% | Nothing to report |
| Establish a fund to guarantee access to microcredit for PSH-MTN | Not yet completed | 0% | Not at that level yet |
| Identify the needs in AGR for PSH-MTN | Made | 100% | Nothing to report |
| Identify PSH-MTNs who should benefit from emergency cash transfers | Made | 100% | Nothing to report |
| Advocate with institutions responsible for monetary transfers to take into account PSH-MTNs | Made | 100% | Nothing to report |
| Establish an inter-sectoral monitoring committee | Made | 100% | Nothing to report |
| Organize an advocacy meeting with the labor inspectorate so that public and private employers apply the laws in favor of the employment of PSH-MTN | Made | 100% | The Labor Inspectorate has promised to make texts available |
| **FIGHT AGAINST STIGMA AND PROMOTION OF RESPECT FOR THE RIGHTS OF PEOPLE WITH DISABILITIES** | | | |
| **IMPROVE THE KNOWLEDGE OF THE POPULATION, INCLUDING PSH-MTN AND CAREGIVERS, ON LAWS AND REGULATIONS PROTECTING PEOPLE WITH DISABILITIES.** | | | |
| Organize awareness sessions through community radio stations (integrated into awareness activities) | Made | 100% | Nothing to report |
| Produce and distribute 500 awareness posters on laws and regulations protecting people with disabilities, specialized care structures and rehabilitation/readaptation services. | Not yet completed | 0% | In progress |
| **PROMOTING THE RIGHTS OF PSH-MTN** | | | |
| Develop and distribute via WhatsApp to PSH-MTN and their helpers a video that presents the rights of people with disabilities. | Not yet completed | 0% | Not at that level yet |
| Organize an advocacy meeting with the authorities in charge of territorial administration and the Regional Directors of the target ministries to raise awareness of the laws and regulations that protect people with disabilities | Made | 100% | Nothing to report |
| Establish a partnership with the Bouaké legal clinic for the management of denials of rights of PSH-MTN | Made | 100% | Nothing to report |
| Organize an advocacy meeting with the Ministry of Justice in Bouaké to obtain its support for the promotion of the rights of PSH-MTNs Make an appointment and meet | Not yet completed | 0% | Not at that level yet |
| **STRENGTHENING THE FIGHT AGAINST STIGMA AND SOCIAL DISCRIMINATION AND PROMOTING THE INCLUSION AND PARTICIPATION OF PSH-NTDs** | | | |
| List and disseminate existing laws and regulations protecting PSH | Made | 100% | Nothing to report |
| List the stigmatizing practices experienced in the project area by PSH-MTNs | Made | 100% | Nothing to report |
| Organize 5 awareness-raising meetings for community leaders on the abandonment of stigmatizing practices by community members | Not yet completed | 0% | In negotiations to have the contacts |
| **PROMOTION OF MUTUAL AID BETWEEN PSH-MTN** | | | |
| **STRENGTHENING THE SELF-HELP CAPACITIES OF PSH-MTN** | | | |
| Train a PSH-MTN/FAHCI pool and caregivers in the psychological care of PSH-MTN – Psychological care module to be administered by HP de Bouake to the participant in half a day | Not yet completed | 0% | Not at that level yet |
| Establish a support team for the preparation of admission files and referral of PSH-MTNs to specialized structures | Completed (10 volunteers) | 100% | Nothing to report |
| Train a pool of 25 people on NTDs to raise awareness on the recognition of suspicious signs, possible complications and management, as well as the laws and regulations that protect people with disabilities (Training of focal points) | Partially completed | 25% | Process in progress |
| Organize training for a pool of PSH-MTNs in the promotion and defense of PSH rights. (Training of focal points) | Not yet completed | 0% | Not at that level yet |
| Organize a meeting to promote self-help groups with PH-MTNs and their caregivers (Send messages via WhatsApp – identify a mutual aid focal point) | Not yet completed | 25% | We act through PSH associations |
| **STRENGTHENING THE ORGANIZATIONAL CAPACITIES OF THE FAHCI ASSOCIATION WITH A VIEW TO THE SUSTAINABILITY OF PSH-MTN ACTIONS** | | | |
| **STRENGTHENING ASSOCIATIVE MANAGEMENT AND RESOURCE MOBILIZATION CAPACITIES TO SUSTAIN PH-MTN ACTIONS** | | | |
| Organize quarterly exchange meetings with the TFPs with a view to improving the administrative, financial and programmatic management of the association | Made | 100% | Nothing to report |
| Partner with a firm to develop and implement a resource mobilization plan to support the execution of the plan | Not yet completed | 0% | Not at that level yet |
| **ENSURE THE COORDINATION, MONITORING AND EVALUATION OF THE PROJECT** | | | |
| Identify focal points at each district level for the implementation of the project | Made | 100% | Nothing to report |
| Develop and implement a project monitoring and evaluation plan | Completed (bi-monthly monitoring and evaluation) | 100% | Nothing to report |
| Equip the coordination unit with IT equipment | Accomplished | 100% | Nothing to report |
| Organize monthly project monitoring meetings with stakeholders | Made | 100% | Nothing to report |

**Source** : March 2023 survey data

The data in this table shows that out of forty-nine (49) activities planned in the logical framework, thirty (30) have been fully completed at 100%. The other nineteen (19) are either partially completed or planned for the coming months. This therefore results in satisfaction to the extent that the activities are completed at 62%. To this end, all stakeholders expressed their satisfaction as evidenced by the respective comments of Coptiment " ***in terms of implementation of activities, I think I am very very satisfied*** " and of PNEL " ***there was a delay but this has been caught up and things are progressing well " (*** Coptiment Representative , Abidjan, March 2023).

## IV-2 Analysis of the relevance of the project (vs. global and specific environment)

The relevance of this project is evident in the methodology used to develop its strategy. Indeed, this strategy was developed by the beneficiaries themselves, who were supported by the national health authorities responsible for the management and governance of the largest NTD control programs in Côte d'Ivoire.

Furthermore, the project strategy was developed following the baseline study which highlighted the specific needs of people affected by NTDs. It is on the scientific basis of this study and its results that the different axes of the strategy were defined.

In general, health projects are based on three major axes: training, research and awareness-raising or communication. The project aimed at the well-being of PWD-NTDs includes these general elements in the implementation process. To succeed in this process, all stakeholders have pooled their efforts through engagement actions with the involvement of various national programs for the improvement of the living and health conditions of people with disabilities due to NTDs. Thus, the importance or usefulness of this project is quite visible in the construction and implementation of this value chain that constitutes all the links ranging increasingly from caregivers, namely CHWs, to program managers and the scientific committee. In a word, it is an integrated project where each stakeholder feels concerned and useful for a common cause: the fight against NTDs.

Specifically, actions are planned and executed according to a program. Meetings are held and activity reports are distributed among stakeholders. At the project area level, volunteers, although lacking significant resources, continue awareness-raising in the localities, advocacy, and census activities to improve the living conditions, access to care, and socio-economic well-being of PSH-NTDs. In addition, the relevance of the project according to Coptiment is demonstrated by:

“ ***The participation of the heads of departments, the various community leaders of Bouaké and the leaders of associations…indeed they held meetings several times with stakeholders and they shared reports ” (*** Coptiment Representative , Abidjan, March 2023).

Such a statement clearly shows that the project is relevant and its implementation reflects the vision of all stakeholders and partners who have so far remained determined for the success of this project.

## IV-3 Analysis of project consistency

As an integrated project, the development of its strategy was based on relevant national frameworks and action plans for the fight against NTDs in Côte d'Ivoire. The strategy and action plan of the Dignité project are fully consistent with Côte d'Ivoire's strategic orientations for the fight against NTDs.

Each institutional entity, association, and program has its assigned roles. However, we noted the existence of administrative burden. Our discussions with stakeholders show that some stakeholders sometimes remain passive, despite knowing their obligations and level of participation in the implementation of this project. This observation prompted the PNEL to react: " ***It's an integrated project, but I don't feel the PNLUB and PNLMTN-CP programs are*** working" (PNEL Representative, Abidjan, March 2023). This implies that these indicated stakeholders seem passive or even inactive, while the project's implementation deadline is drawing to a close. As a result, these partners must review their position and work to achieve the set objectives. It should be noted that this burden is also reflected in the PNLMTN-CP's comments:

" ***So as they said, it hasn't really been intensified and promulgated yet, or how can I put it, on a large scale. But I do know that they had entrusted this activity to a structure on site in Bouaké. So we are at the same level of information as the people from the departmental management (....). It should be intensified and now the activities carried out must be shared with others so that we can perhaps all really be at the same level of information*** " (PNLMTN Representative, Abidjan, March 2023).

The contradiction that arises from this statement in relation to the information on the progress of the execution of the project justifies the implication of this program.

Apart from this observation, the implementation of the project is progressing well. At the external level, the beneficiaries we interviewed are still waiting for concrete actions to be taken. To this end, the PSH-MTN, Mr. YKL and Mr. KY, put forward the following:

“ ***FAHCI came to see us in our village. They wrote down our names and took the contact details of each NTD patient. They promised to come back, but so far we haven't seen anything. Here, there is no health center that treats our illness. And then when we go to the Rafiékro health center , the health workers don't treat us. So it's difficult because we're suffering*** ” (FAHCI Representative, Abidjan, March 2023).

This statement shows that the actions taken towards PWD-NTDs have not yet met their expectations. They expect actions that could positively impact their living conditions and health. But this is slow to materialize. This constitutes a source of pain for these people that they cannot understand, much less endure any longer.

## IV-4 Analysis of project effectiveness (planning and execution)

Project effectiveness is observed in planning and execution, which is broken down into four specific objectives. Each specific objective includes activities whose successful execution helps to understand the effectiveness of the project.

***Os1- advocacy and awareness raising for improving access to physical, psychosocial, educational and economic rehabilitation services***

This first objective, composed of twenty-nine (29) activities, has an acceptable completion rate of 65.51%, meaning 19/29 activities were completed. Such a result demonstrates the commitment of the stakeholders involved in the implementation of the project and that of the administrative authorities, department heads and community leaders. Added to this is the dynamism of the project team, which works tirelessly to achieve the objectives. This is also evidenced by the words of Coptiment : " ***I confirm that we have indeed had a commitment signature from the stakeholders. "***» ( Coptiment Representative , Abidjan, March 2023). The FAHCI coordinator adds by specifying the approach followed in achieving the activities of this objective:

" ***We organized a meeting with the prefecture, with each departmental director of the health district, also meetings with each focal point of the health districts, there you go, meetings also with institutions, such as the legal clinic, the labor inspectorate, the youth employment agency, the DRENA too. They did not receive us completely. So the meetings are taking place. I even had to do a focus group in a village because we do radio broadcasts in this village. I was called a lot*** " (FAHCI Representative, Abidjan, March 2023).

In addition to mass and community meetings, project implementers conduct awareness sessions via Bouaké's local radio station. These awareness-raising messages provide information on physical, psychological, psychosocial, educational, and economic rehabilitation services, as well as the access procedure to follow. The messages also provide details on the signs for recognizing NTDs, the treatment procedure, and specific reception centers.

***Os2 - fight against stigmatization and promote respect for the rights of people with disabilities***

This objective is broken down into nine (9) activities, five (5) of which have been completed, representing a completion rate of 55.55%. The fight against stigma is carried out through radio awareness sessions and the sharing of awareness leaflets and posters. These awareness campaigns include legal components that inform the target audience about the rights of people with disabilities due to MTN.

***Os3- promotion of mutual aid between*** ***PSH-MTN***

To this end, five (5) activities were planned, but only one (1) was fully implemented. This involves setting up a support team to compile admission files and refer PSH-MTNs to specialized structures. This result gives us a proportion of 20% in terms of achievement. As a result, the promotion of mutual assistance between PSH-MTNs has not really begun. Certainly, the FAHCI works with associations of People with Disabilities, as indicated in the following statement: " ***We collaborate with PSH-MTN structures*** " (FAHCI Representative, Abidjan, March 2023), but the actors involved in the implementation of the project must make every effort to strengthen the self-help capacities of PSH-MTNs.

***Os4- strengthening the organizational capacities of the FAHCI association with a view to the sustainability of PSH-MTN actions***

The activities of this objective are largely implemented. Thus, out of a total of six (6) activities, five (5) have been completed. This gives a proportion of 83.33% of completion. The FAHCI has therefore implemented certain actions to strengthen the capacities of its actors in terms of associative management, coordination, monitoring and evaluation of the project. However, the question of mobilizing resources for the sustainability of PSH-MTN actions has not yet emerged. At this level, the coordinator reassured us that this will not be long because they are not yet at this level.

## IV-5 Analysis of the strengths and weaknesses of the project

Like all human endeavors, the Dignity Project has strengths and weaknesses in its implementation. Our analysis will highlight these two aspects of the project under each heading.

**Project management bodies**

The strengths relating to the implementation of the project lie in the commitment of the various project management bodies, especially the FAHCI team which meets and interacts daily with the PSH-MTNs, as the coordinator points out when speaking about the strengths of the project:

" ***We have the commitment of the team on site, myself and my volunteers. There is also the commitment of the structures, the institutions; the majority of institutions are welcoming and present on site. The availability of focal points and the district DDs*** " (FAHCI Representative, Abidjan, March 2023).

The commitment and availability of all the stakeholders mentioned constitute a strength in the realization of this project. Through the data collection sessions, we observed a dynamism at the level of each stakeholder for the success of the project. Apart from the dynamism and commitment, the other strength is that the project is part of a scientific study. As a result, the actions of each management body are well determined. Moreover, this is the position of the co-management :

“ ***For me, one of the strengths of the project is that we have carried out scientific studies to determine the needs of people, not with people with disabilities. That is one of the strengths of the project. Starting with the identification of the needs of people with disabilities by meeting with them… then the development of strategies for mobilizing people with disabilities in order to improve access to the service… with people with disabilities ” (*** Coptiment Representative , Abidjan, March 2023).

Another strength results from the communication mechanism insofar as all entities are informed of the activities carried out on the ground. To this end, the PNEL emphasizes that: " ***the program is informed of everything that is done in the implementation of the project*** " (PNEL Representative, Abidjan, March 2023).

Regarding the weaknesses of the management bodies, the first concerns the functioning of the scientific committee, which has experienced difficulties. Indeed, the President, as coordinating director of national programs to combat Buruli ulcer and leprosy, was dismissed from his duties in December 2022. Being a government authority, the replacement process took place over a period of two (02) months. It is also important to point out that the replacement, once in office, focused his priorities on the organization of World Leprosy Days. This is why, until now, he has not been able to fully fulfill the functions of president of the scientific committee. Such a situation makes the meetings of the scientific committee problematic. As a result, the delivery of project implementation documents is not effective. The documents do not always reach the recipients. In a word, the dysfunction of the scientific committee constitutes a weakness in the general functioning of the project management bodies.

Beyond the problem related to the functioning of the scientific committee, we have the passivity of the PNLUB and PNLMTN as mentioned above. Through our meetings, we have noted that they do not feel sufficiently concerned by the project. This is evidenced by the PNLMTN's comments:

" ***We don't go directly because it's the FAHCI as a structure that carries out the activities. But that doesn't prevent us from having the directives, how to do it, we are really informed and we try to identify a little the needs of the patients who are affected... I think they have launched the activities and we are informed*** " (PNLMTN Representative, Abidjan, March 2023).

At this level, the programs mentioned should be actively involved in the implementation of this project because they are directly concerned with the pathologies of the victims. In addition, some structures were difficult to access, but with the involvement of the administrative authorities, this obstacle was overcome.

**Project implementation device**

Speaking of implementation mechanisms, we have identified several strengths. These include the mobility of the FAHCI team and the availability of other ministries involved, who respond when a challenge arises. For example, the legal clinic, the labor inspectorate, and other organizations say they are ready to support the FAHCI and its partners for the success of this project. Such perspectives and willingness will only facilitate the project's implementation.

However, we noted several difficulties in the implementation of the project. The first is the budget, which is very limited, as the co-author points out :

“ ***The main difficulty is budgetary. The budget allocated to the implementation of the project was low. So it couldn’t really cover what needed to be done properly. It would have been normal to recruit the coordinator, the monitoring and evaluation manager, the financial manager. We were only able to recruit two people ” (*** Coptiment Representative , Abidjan, March 2023).

This situation raises the personnel problem, which is the second difficulty. It is the basis for the recruitment of ten (10) unpaid volunteers. Only these people benefit from their transportation, which allows them to go to the villages according to the schedule. It is important to emphasize that the coordinator does not have a salary but has agreed to work for the well-being of PSH-NTDs. Other skills such as the monitoring and evaluation manager and the one intended for the management of financial matters were not recruited due to lack of sufficient budget.

The third difficulty is related to the team's mobility. Indeed, the team set up to carry out the project does not have any mobility equipment. It feels obliged to use either motorcycle taxis, with all the risks involved, or bush taxis, which do not have a fixed mobility program. As a result, it is often late to meeting points. This impacts daily or weekly performance. It would be appropriate for the team to have a double-cab pickup car , motorcycles, and fuel vouchers to facilitate its travel to the villages. This would be a breath of fresh air for the successful implementation of the project.

**Project implementation**

The activities are carried out by the FAHCI team in collaboration with all stakeholders, including the various national programs that are linked to the issue of NTDs. This system has strengths and weaknesses that it is wise to highlight.

Speaking of strengths, we first have the communication strategy, which is very effective. Indeed, radio awareness campaigns are conducted twice a week instead of once a quarter as planned in the logical framework activities. The coordinator describes this program in these terms:

" ***We signed a seven-month contract even because we started in December 2022. We signed a seven-month contract with Radio Bouaké. Every Tuesday from 9 a.m. and every Friday from 4 p.m., that is, twice a week... the rebroadcast Tuesday at 11 p.m. and Friday at 8 p.m.*** " (FAHCI Representative, Abidjan, March 2023).

This awareness-raising strategy has a wide reach in that parents of NTD patients call FAHCI to register and involve them in the project. The second strength of the implementation of this project is the availability of a database of PSH-NTDs, the identification and census of their specific needs as well as the statistics of children with disabilities of school age. The third strength is the involvement of PSH-NTDs in the implementation of the project. To this end, the coordinator and volunteers work in close collaboration with PSH-NTD associations.

Regarding the weaknesses of this implementation, the first is that health workers are not yet trained to care for PWD-NTDs. This constitutes a major problem for the regular care of NTD patients in the different localities. In addition, specialized centers to accommodate the target have not yet been identified and assistance funds for people likely to benefit from any support are not yet available. As a result, promises of assistance are not kept and PWD-NTDs are asking themselves several questions, especially those who are isolated and live in precarious conditions. This is evidenced by the concerns expressed by these PWD-NTDs:

“ **The illness doesn’t allow us to work and we don’t have money to treat ourselves. Today, everything has become a matter of money. Our concern is when the people from FAHCI will come back to us and what will they do for us** ?” (PSH-MTN representative, Abidjan, March 2023).

This statement by PWDs clearly shows that they expect support or assistance that will enable them to ensure their survival and, above all, to access better living conditions. It is a way of expressing their heartfelt cry while describing the extent of the health and existential situation of PWD-NTDs.

The third weakness is the lack of material and financial support from partners who have expressed a willingness to support the implementation of the project. In addition, the low funding of the project prevents FAHCI from carrying out actions proportional to the realities of the project target. All of these weaknesses impact FAHCI's performance while slowing the momentum of the project's implementing team. However, actions should be carried out increasingly in order to improve the conditions of access to care through free care, and the socio-economic situation of PSH-MTNs.

## IV-6 Analysis of the potential sustainability of project actions

What matters in this pilot project is the sustainability of the achievements. Currently, radio awareness campaigns are going very well. But what can help preserve the achievements and, above all, ensure the potential sustainability of the project's actions is the support of PWD-NTDs. This support must be provided through PWD-NTD associations. This will strengthen trust, cohesion, mutual support, and collaboration both among PWD-NTDs and between them and the various stakeholders and their specific partners. To this end, the strategies to be adopted are those of mass assistance and proximity actions.

## IV-7 Lessons learned and good practices

Several lessons and good practices have emerged from the implementation of this project on PSH-NTDs. The project coordinator provides some answers to these questions:

**“Self-sacrifice… sacrifice for people with disabilities. You need that, and also the ability to work under pressure”** (FAHCI Representative, Abidjan, March 2023) **.**

From this statement, we can see that the essential lessons are, first of all, self-sacrifice, courage, and sacrifice. This assumes that the actors of the implementation team work voluntarily without regard for a possible salary. Their attention is focused on the well-being of PSH-MTN. Thus, they build a capacity to work under pressure, that is, without respite and rest. This has allowed them to develop resilience and resistance capacities conducive to facing the problems and emergency situations that the target constantly raises. It also results from this evaluation that the team demonstrated an organization that led it to meet the requirements of the project. It has thus maintained good collaboration with the institutions and the various associations of people with disabilities in order to achieve its objectives.

# Conclusions and recommendations for successful achievement of results for the remaining period

Ultimately, we can conclude that the mid-term evaluation of the Dignity project allowed us to identify the progress in the implementation of the activities identified in the logical framework document. The majority of stakeholders involved in the implementation of this project expressed their satisfaction with the progress of the work. While there are some bottlenecks at the level of the scientific committee as well as the programs and support partners, a good part of the objectives is being achieved. This leads us to say that at the end of this project the objectives will not be fully achieved but only partially. It is therefore important to preserve the achievements by implementing strategies that are in line with the realities on the ground. Thus, to successfully complete this project in order to achieve the objectives, we make the following recommendations:

1. Increase the overall budget allocated to the execution of the project in order to make the implementation team more operational,

1. Provide the project team with sufficient means of mobility (vehicles, motorcycles, etc.) and fuel vouchers for its travel in the various districts and specific localities,
2. Provide the project team with additional equipment to strengthen it,
3. Review the functioning of the scientific committee in order to improve its collaboration with the other stakeholders in the project while prioritizing the sustainability of the achievements,
4. Provide the team with a monthly volunteer support fund,
5. Encourage and motivate support partners to act for the well-being of PSH-MTN,
6. Begin supporting PSH-MTNs by using their respective associations as support,
7. Include in the project implementation process the visit of each program to the target to better assess the achievement of the objectives,
8. Raise awareness among the FAHCI so that it takes ownership of the project by perpetuating the achievements for its extension throughout the Ivorian territory.

# Bibliography

-Mid-term evaluation study protocol for the dignity project, 2023, Identification of a strategy to increase the influence of people affected by NTDs on access to care services for people affected by NTDs in Côte d'Ivoire

-Study report, 2022, Identification of the specific needs of people with disabilities due to Neglected Tropical Diseases (PSH-NTDs) in Côte d'Ivoire

-DIDR-OFPRA Report: People with disabilities - Ivory Coast 2018

-National Health Development Plan (NHDP) 2016-2020

# Annexes

**Appendix 1 : Interview guide**

| **Mid-term evaluation of the Dignity Project**  “Identification of effective strategies and mechanisms to increase the influence of people affected by NTDs on access to care services for people affected by NTDs in Côte d’Ivoire” | | |
| --- | --- | --- |
| **March 2023** | **QUIZ** | **Final version** |

The information contained in this questionnaire is confidential. It is covered by statistical confidentiality and may only be published anonymously in accordance with Law No. 2013-537 of July 10, 2013, on the organization of the National Statistical System.

Investigator Name: _____________________ Date: /___/____/_________/

Enter the target: ______________________

| **No.** | **QUESTIONS** | **Answers** | **TARGET** |
| --- | --- | --- | --- |
| **AXIS 1** | **ADVOCACY AND AWARENESS RAISING FOR IMPROVED ACCESS TO PHYSICAL, PSYCHOSOCIAL, EDUCATIONAL AND ECONOMIC REHABILITATION SERVICES** | | |
| **1** | **COMMITMENT OF ADMINISTRATIVE AUTHORITIES AND COMMUNITY LEADERS TO THE IMPLEMENTATION OF THE STRATEGY** | | |
| **Q1.1.1** | Did you organize a project launch ceremony to raise awareness among the administrative authorities and community and association leaders in Bouaké? | 1= Yes  2= No, if not why? | FAHCI / Scientific Committee / Coptiment . |
| **Q1.1.2** | Have you secured stakeholder commitment to improving the quality of life of PSH-MTNs? | 1= Yes  2= No, if not why? | FAHCI / Scientific Committee / Coptiment |
| **Q1.1.3** | Have you organized at least one meeting with each institution to strengthen collaboration? | 1= Yes  2= No, if not why? | FAHCI / Scientific Committee / Coptiment |
| **2** | **ADVOCACY FOR FREE CARE INCLUDING REHABILITATION AND REHABILITATION CARE FOR PSH-MTN** | | |
| **Q1.2.1** | Have you submitted a request to the Ministry of Health and the Ministry of Social Protection for free coverage of rehabilitation and readaptation care for PSH-MTN? | 1= Yes  2= No, if not why? | Presidency of the FAHCI |
| **Q1.2.2** | Did you follow up on the letters through at least one meeting with the authorities of the target institutions (Health and Social Protection)? | 1= Yes  2= No, if not why? | FAHCI |
| **3** | **STRENGTHENING PSYCHOLOGICAL CARE FOR PSH-MTN** |  |  |
| **Q1.3.1** | Have you organized an advocacy meeting targeting health district authorities so that psychological care is integrated into the care of PSH-MTNs? | 1= Yes  2= No, if not why? | FAHCI |
| **Q1.3.2** | Have you followed up with the PNEL for the training of health workers regarding this project? | 1= Yes  2= No, if not why? | FAHCI / Focal points |
| **Q1.3.3** | Did you participate in planning meetings on NTD control in health districts and at central level? | 1= Yes  2= No, if not why? | FAHCI / Focal points |
| **4** | **IMPROVING THE KNOWLEDGE AND ATTITUDES OF POPULATIONS, PWDs AND CAREGIVERS ON NTDs AND LAWS AND REGULATIONS PROTECTING PWDs.** |  |  |
| **Q1.4.1** | Have you organized a quarterly radio program to raise awareness about NTDs, recognizing suspicious signs, possible complications and management, as well as laws and regulations that protect people with disabilities? | 1= Yes  2= No, if not why? | FAHCI |
| **Q1.4.2** | Have you shared videos on WhatsApp and Facebook on raising awareness about NTDs, recognizing suspicious signs, possible complications and management, as well as laws and regulations that protect people with disabilities? | 1= Yes  2= No, if not why? | FAHCI |
| **Q1.4.3** | Have you organized advocacy meetings with community leaders to remove cultural barriers and/or improve collaboration with the health system for the management of PSH-MTNs? | 1= Yes  2= No, if not why? | FAHCI |
| **Q1.4.4** | Did you participate in the planning meetings on NTD control in the health districts? | 1= Yes  2= No, if not why? | FAHCI / Health districts / PNEL / PNLUB |
| **5** | **MOBILIZATION OF RESOURCES FOR THE INTEGRATION OF CHILDREN WITH DISABILITIES DUE TO NTDs INTO THE EDUCATION SYSTEM THROUGH ADVOCACY AND SCHOOL SUPPORT** |  |  |
| **Q1.5.1** | Did you identify children with disabilities due to NTDs who had schooling needs during the PSH-NTD census with the help of focal points? | 1= Yes  2= No, if not why? | FAHCI |
| **Q1.5.2** | Have you organized an advocacy meeting with national education officials for the admission of children in need into schools? | 1= Yes  2= No, if not why? | FAHCI |
| **Q1.5.3** | Have you organized a meeting to mobilize financial resources with financial partners and government aid agencies to obtain support for PSH-MTNs? | 1= Yes  2= No, if not why? | FAHCI |
| **6** | **THE PROMOTION OF SPECIALIZED STRUCTURES IN THE EDUCATION OF PH TO PSH-MTN** |  |  |
| **Q.6.1** | Have you identified the existing specialized structures in Ivory Coast? | 1= Yes  2= No, if not why? | FAHCI |
| **Q1.6.2** | Have you sent awareness-raising letters and requests for admission of PSH-MTNs to the identified structures? | 1= Yes  2= No, if not why? | FAHCI |
| **Q1.6.3** | Have you made PWD of school-age or training aware of the existence of specialized education structures and the conditions of access to them? | 1= Yes  2= No, if not why? | FAHCI |
| **Q1.6.4** | Do you have a PH-MTN WhatsApp address book for sharing information? | 1= Yes  2= No, if not why? | FAHCI |
| **7** | **IMPROVING ACCESS TO TRAINING FOR ADULTS WITH DISABILITIES FROM NTDs** |  |  |
| **Q1.7.1** | Have you produced and distributed leaflets (in digital and physical form) to PSH-MTNs that provide information on literacy and vocational training opportunities? | 1= Yes  2= No, if not why? | FAHCI |
| **Q1.7.2** | Have you organized an awareness-raising meeting for education system officials on the application of laws relating to access to literacy and vocational training for people with disabilities? | 1= Yes  2= No, if not why? | FAHCI |
| **8** | **STRENGTHENING THE FINANCIAL CAPACITY (EMPLOYMENT) OF PSH-MTN AND CAREGIVERS THROUGH PERIODIC CASH TRANSFERS, THE DEVELOPMENT OF AGR AND THE PROMOTION OF SELF-EMPLOYMENT** |  |  |
| **Q1.8.1** | Have you identified partners for financial and technical support for people with disabilities? | 1= Yes  2= No, if not why? | FAHCI |
| **Q1.8.2** | Have you established collaboration agreements with two institutions specializing in the promotion of self-employment? | 1= Yes  2= No, if not why? | FAHCI |
| **Q1.8.3** | Has a fund been set up to guarantee access to microcredit for PSH-MTNs? | 1= Yes  2= No, if not why? | FAHCI |
| **Q1.8.4** | Have you identified the needs in AGR for PSH-MTN? | 1= Yes  2= No, if not why? | FAHCI |
| **Q1.8.5** | Have you identified the PSH-MTNs who should benefit from emergency cash transfers? | 1= Yes  2= No, if not why? | FAHCI |
| **Q1.8.6** | Have you made any pleas to the institutions responsible for monetary transfers to take into account PSH-MTNs? | 1= Yes  2= No, if not why? | FAHCI |
| **Q1.8.7** | Has an inter-sectoral monitoring committee been set up? | 1= Yes  2= No, if not why? | FAHCI |
| **Q1.8.8** | Have you organized an advocacy meeting with the labor inspectorate so that public and private employers apply the laws in favor of the employment of PSH-MTN? | 1= Yes  2= No, if not why? | FAHCI |
| **AXIS 2** | **FIGHT AGAINST STIGMA AND PROMOTION OF RESPECT FOR THE RIGHTS OF PEOPLE WITH DISABILITIES** | | |
| **1** | **IMPROVING THE KNOWLEDGE OF THE POPULATION, INCLUDING PSH-MTN AND CAREGIVERS, ON LAWS AND REGULATIONS PROTECTING PEOPLE WITH DISABILITIES.** |  |  |
| **Q2.1.1** | Have you organized awareness sessions through community radios (integrated into awareness activities)? |  |  |
| **Q2.1.2** | Have you produced and distributed 500 awareness posters on laws and regulations protecting people with disabilities, specialized care structures and rehabilitation/rehabilitation services? | 1= Yes  2= No, if not why? | FAHCI |
| **2** | **PROMOTION OF THE RIGHTS OF PSH-MTN** |  |  |
| **Q2.2.1** | Have you developed and disseminated through WhatsApp to PSH-MTN and their helpers a video that presents the rights of people with disabilities? |  |  |
| **Q2.2.2** | Have you organized an advocacy meeting with the authorities in charge of territorial administration and the Regional Directors of the target ministries to raise awareness about the laws and regulations that protect people with disabilities? | 1= Yes  2= No, if not why? | FAHCI / Bouaké Legal Clinic |
| **Q2.2.3** | Have you established a partnership with the Bouaké legal clinic for the management of denials of rights of PSH-MTN? | 1= Yes  2= No, if not why? | FAHCI |
| **Q2.2.4** | Have you organized an advocacy meeting with the Ministry of Justice in Bouaké to obtain its support for the promotion of the rights of PSH-MTN or a meeting and appointment? | 1= Yes  2= No, if not why? | FAHCI / PSH-MTN |
| **3** | **STRENGTHENING THE FIGHT AGAINST STIGMA AND SOCIAL DISCRIMINATION AND PROMOTING THE INCLUSION AND PARTICIPATION OF PSH-MTN** |  |  |
| **Q2.3.1** | Have you listed and disseminated existing laws and regulations protecting PSH? | 1= Yes  2= No, if not why? | FAHCI |
| **Q2.3.2** | Have you listed the stigmatizing practices experienced in the project area by PSH-MTNs? | 1= Yes  2= No, if not why? | FAHCI |
| **Q2.3.3** | Have you organized 5 awareness meetings for community leaders on the abandonment of stigmatizing practices by community members? | 1= Yes  2= No, if not why? | FAHCI |
| **AXIS 3** | **PROMOTION OF MUTUAL AID BETWEEN PSH-MTN** |  |  |
| **1** | **STRENGTHENING THE SELF-HELP CAPACITY OF PSH-MTN** |  |  |
| **Q3.1.1** | Have you trained a PSH-MTN/FAHCI pool and caregivers in the psychological care of PSH- MTN?  (Psychological care module to be administered by HP de Bouake to the participant in half a day) | 1= Yes  2= No, if not why? | FAHCI / PNSM |
| **Q3.1.2** | Have you set up a support team to compile admission files and refer PSH-MTNs to specialized structures? | 1= Yes  2= No, if not why? | FAHCI |
| **Q3.1.3** | Train a pool of 25 people on NTDs to raise awareness on the recognition of suspicious signs, possible complications and management, as well as the laws and regulations that protect people with disabilities (Training of focal points) | 1= Yes  2= No, if not why? | FAHCI |
| **Q3.1.4** | Have you organized training for a pool of PSH-MTNs on the promotion and defense of PSH rights? (Training of focal points) | 1= Yes  2= No, if not why? | FAHCI |
| **Q3.1.5** | Have you organized a meeting to promote self-help groups with PH-MTNs and their caregivers? (Pass messages via WhatsApp – identify a mutual aid focal point) | 1= Yes  2= No, if not why? | FAHCI |
| **AXIS 4** | **STRENGTHENING THE ORGANIZATIONAL CAPACITIES OF THE FAHCI ASSOCIATION WITH A VIEW TO THE SUSTAINABILITY OF PSH-MTN ACTIONS** | | |
| **1** | **STRENGTHENING ASSOCIATIVE MANAGEMENT CAPACITIES AND RESOURCE MOBILIZATION TO SUSTAIN THE ACTIONS OF PSH-MTN** |  |  |
| **Q4.1.1** | Have you organized quarterly exchange meetings with the TFPs with a view to improving the administrative, financial and programmatic management of the association? | 1= Yes  2= No, if not why? | FAHCI |
| **Q4.1.2** | Have you partnered with a firm to develop and implement a resource mobilization plan to support the execution of the plan? | 1= Yes  2= No, if not why? | FAHCI |
| **2** | **PROJECT COORDINATION, MONITORING AND EVALUATION** |  |  |
| **Q4.2.1** | Have you identified focal points at each district level for the implementation of the project? | 1= Yes  2= No, if not why? | FAHCI |
| **Q4.2.2** | Have you developed and implemented a project monitoring and evaluation plan? | 1= Yes  2= No, if not why? | CESI/Mr. Lorou Bi |
| **Q4.2.3** | Equip the coordination unit with IT equipment | 1= Yes  2= No, if not why? | Coptiment |
| **Q4.2.4** | Do you hold monthly project monitoring meetings with stakeholders? | 1= Yes  2= No, if not why? | Coptiment |

What other difficulties did you encounter in implementing the project?

What strengths did you identify in implementing this project?

What lessons have been learned?

What recommendations would you make for the successful completion of the pilot project?

Do you think you could achieve your goals by carrying out all the activities listed in the project action plan?

Thank you for participating in this study.

END
