# Supplementary material for: People-centered strategies to mobilize people living with disabilities due to Neglected Tropical Diseases (PD-NTDs) to influence policy and programs: A mixed-methods study in Côte d’Ivoire
Source: PLoS Negl Trop Dis. 2025 Sep 8;19(9):e0013485. doi: 10.1371/journal.pntd.0013485 (PMC12431663; doi:10.1371/journal.pntd.0013485)
Supplement: S2 Data — (DOCX) [file pntd.0013485.s006.docx]

**REPUBLIC OF IVORY COAST**

UNION – DISCIPLINE – WORK

**Final Evaluation Study Report of the**

**Dignity Pilot Project**

Identification de stratégie pour accroitre l'influence des personnes affectées par les Maladies Tropicales Négligées sur leur accès aux services de prise en charge en Côte d'Ivoire

**February 28, 2024**

| **Presented by:** | **CESI** (Statistical and Computer Science Studies Firm)  Tel: (+225) 27 21 38 18 02  Mobile: (+225) 07 08 27 37 38  **Email :** [cabstat_info@yahoo.fr](mailto:cabstat_info@yahoo.fr) / infos@cesi-ci.com |
| --- | --- |

**Final Evaluation of the Dignity Project**

“Identification of a strategy to increase access to care services for people affected by Neglected Tropical Diseases in Côte d’Ivoire”

| ***Search site:*** | Gbêkê health region, in central Ivory Coast |
| --- | --- |
| ***Type of study:*** | Cross-sectional study |
| ***Provider :*** | Statistical and Computer Science Studies Office (CESI) |
| ***Study Team:*** | - LOROU Bi Gohoré Jean-Maxime, Principal Consultant, Statistical Works Engineer, Specialist in Project Monitoring and Evaluation, Associate Director of CESI, - TANOH Armand Hira, Associate Consultant, Statistician, Specialist in Project Monitoring and Evaluation, Director of CESI; - Dr. N'DRI Kouadio Patrice, Socio-anthropologist, Associate Consultant, Research Professor at the Alassane Ouattara University of Bouaké-Côte d'Ivoire - Dr Julien Aké, Associate Director of Coptiment , Doctor, Specialist in Community Health, Expert in the fight against Neglected Tropical Diseases (NTDs), - Dr Boko Nadège Koidia , Associate Researcher, Researcher in Social Sciences, Felix Houphouët Boigny University, Consultant at Coptiment - Prof. Isaac TIEMBRE, Associate Researcher, Researcher in Public Health and Specialist in Research Methodology, Felix Houphouët Boigny University, Consultant at Coptiment |
| ***Study period:*** | September 2023 – February 2024 |
| ***Study Sponsor:*** | COPTIMENT (Business Growth and Optimization) Ivory Coast |
| ***Members of the scientific committee*** | - Dr. Dizoe Agui Sylvestre, Director of the National Leprosy Elimination Program; - Dr Julien Aké, Associate Director of Coptiment , Doctor, Specialist in Community Health, Expert in the fight against Neglected Tropical Diseases (NTDs), - Dr Koffi Aboa , Representative of the PNLUB, - Dr Dje Norbert, Coordinating Director of PNLMTN-CP, - Pr Isaac TIEMBRE, Associate Researcher, Researcher in Health and Public Health and Specialist in Research Methodology, Felix Houphouët Boigny University, Consultant at Coptiment , - Dr Boko Nadège Koidia , Associate Researcher, Researcher in Social Sciences, Felix Houphouët Boigny University, Consultant at Coptiment , - Ms. Konan, Representative of the Federation of Associations of the Disabled of Côte d' Ivoire , - Maneesh Philip, Director of Programs, Effect:Hope Canada. |

**Table of Contents**

[List of abbreviations or acronyms](#_Toc158010359)  [3](#_Toc158010359)

[Executive Summary](#_Toc158010360)  [4](#_Toc158010360)

[I- Introduction](#_Toc158010361)  [8](#_Toc158010361)

[I.1. Presentation of the context of the project](#_Toc158010362)  [8](#_Toc158010362)

[I.2. Scientific justification/rationale for the study](#_Toc158010363)  [8](#_Toc158010363)

[I.3. Implementation](#_Toc158010364)  [9](#_Toc158010364)

[II- Objectives of the research](#_Toc158010365)  [9](#_Toc158010365)

[II.1. General Objective](#_Toc158010366)  [9](#_Toc158010366)

[II.2. Specific Objectives :](#_Toc158010367)  [9](#_Toc158010367)

[III- Methodology](#_Toc158010368)  [10](#_Toc158010368)

[III.1 Method and tools](#_Toc158010369)  [10](#_Toc158010369)

[**III.1-1 The documentary review**](#_Toc158010370)  [10](#_Toc158010370)

[**III.1-2 The qualitative survey**](#_Toc158010371)  [10](#_Toc158010371)

[**III.1-3 Assessment Area and Targets**](#_Toc158010372)  [13](#_Toc158010372)

[III.2 Data collection](#_Toc158010373)  [13](#_Toc158010373)

[III.3 Data processing and report writing](#_Toc158010374)  [14](#_Toc158010374)

[IV- Results of the final evaluation of the pilot project](#_Toc158010375)  [15](#_Toc158010375)

[IV.1 Brief presentation of project](#_Toc158010376)  [15](#_Toc158010376)

[IV.2 Analysis of the relevance of the project (vs. global and specific environment)](#_Toc158010377)  [16](#_Toc158010377)

[IV.3 Analysis of project consistency (internal and external)](#_Toc158010378)  [17](#_Toc158010378)

[IV-4 Analysis of the effectiveness of the project](#_Toc158010379)  [19](#_Toc158010379)

[**IV-4-1 Overall level of execution of project activities**](#_Toc158010380)  [19](#_Toc158010380)

[**IV.4.2 Level of achievement of project objectives**](#_Toc158010381)  [28](#_Toc158010381)

[IV.5 Analysis of the effects of the project on People affected by NTDs](#_Toc158010382)  [31](#_Toc158010382)

[**IV.5.1 At the level of knowledge and attitudes**](#_Toc158010383)  [32](#_Toc158010383)

[**IV.5.2 At the level of the health situation of PVMTN**](#_Toc158010384)  [32](#_Toc158010384)

[**IV.5.3 At the level of the socio-economic situation of PVMTN**](#_Toc158010385)  [33](#_Toc158010385)

[**IV.5.4 At the level of actor satisfaction**](#_Toc158010386)  [34](#_Toc158010386)

[**IV.5.5 At the level of situation analysis Before versus After the project**](#_Toc158010387)  [36](#_Toc158010387)

[IV.6 Analysis of the strengths and weaknesses of the project](#_Toc158010388)  [37](#_Toc158010388)

[IV.7 Analysis of the potential sustainability of project actions](#_Toc158010389)  [40](#_Toc158010389)

[IV.8 Lessons learned and good practices](#_Toc158010390)  [41](#_Toc158010390)

[Conclusion and recommendations for the sustainability and survival of project](#_Toc158010391)  [42](#_Toc158010391)

[Bibliography](#_Toc158010392)  [43](#_Toc158010392)

[Annexes](#_Toc158010393)  [45](#_Toc158010393)

[Information Notice](#_Toc158010394)  [45](#_Toc158010394)

[Written and informed consent form](#_Toc158010395)  [47](#_Toc158010395)

[Confidentiality Agreement](#_Toc158010396)  [48](#_Toc158010396)

[Data collection tools](#_Toc158010397)  [49](#_Toc158010397)

#

# **List of abbreviations or acronyms**

| AMM / AMD | : | Mass Drug Administration |
| --- | --- | --- |
| BIT | : | International Labour Office |
| CAP | : | Knowledge Attitudes and Practices |
| CAPC | : | Knowledge, Attitudes, Practices and Beliefs |
| CESI SARL | : | Statistical and IT Research Firm, Limited Liability Company |
| CI | : | Ivory Coast |
| COOPEC |  | Savings and Credit Cooperative |
| COPYING |  | Business Growth and Optimization |
| COVID 19 | : | Coronavirus disease |
| CSAS | : | Head of Health Action Service |
| DSC | : | Community Health Department |
| FAHCI | : | Federation of Associations of Disabled People of Ivory Coast |
| FL |  | Lymphatic filariasis |
| LRI | : | Leprosy Research Initiative |
| MC | : | Skin Manifestation |
| MTN | : | Neglected Tropical Disease |
| ODK | : | Open Data Kit |
| NGO | : | Non-Governmental Organization |
| PNDS |  | National Health Development Plan (NHDP) |
| PNEL | : | National Leprosy Elimination Program |
| PNLMTN CP | : | National Program for the Fight against Neglected Tropical Diseases with Preventive Chemotherapy |
| PNLUB | : | National Program to Combat Buruli Ulcer |
| PSH-MTN |  | Person with Disabilities due to Neglected Tropical Diseases |
| PVMTN |  | Person Living with Neglected Tropical Disease |
| RAP |  | Participatory Action Research |
| RTI | : | Ivorian Radio and Television |
| THA | : | Human African Trypanosomiasis |
| HIV |  | Human immunodeficiency virus |

# **Executive Summary**

Côte d'Ivoire is endemic to 14 Neglected Tropical Diseases, including leprosy and Buruli ulcer. The study report on the identification of the specific needs of people with disabilities due to Neglected Tropical Diseases (PWD-NTDs) in Côte d'Ivoire, conducted in 2022 as part of the project's baseline study, provides a general overview of the needs, knowledge, attitudes, practices and beliefs (KAPC) of affected people, their caregivers and relevant key informants in responding to the needs of PWD-NTDs.

This baseline study identified the following main needs among people with disabilities due to NTDs:

- Health care, rehabilitation, readaptation and psychosocial support;
- Information – awareness on NTDs and the rights of PWDs;
- Schooling and professional training of PSH-MTN;
- Socio-professional integration and support for self-employment;
- Income Generating Activities (IGA) and income support.

Furthermore, this basic study highlighted the following CAPCs:

- Poor knowledge of PSH-MTNs on the causes and modes of transmission of MTNs, the construction of prejudices and the presence of sociocultural barriers;
- Poor knowledge of PSH-MTN on specialized care structures and rehabilitation/readaptation services;
- Low knowledge of PSH-MTN on laws and regulations protecting people with disabilities;
- Negative attitudes of PSH-MTN towards barriers, prejudices about disability and MTNs, stigma and social discrimination;
- Lack of enabling policies and environment to address barriers, prejudices about disability and NTDs, stigma and social discrimination;
- Low level of organization of PSH-MTNs and low capacity for advocacy and empowerment of PSH-MTN Organizations.

Using the results of this baseline study, a strategy aimed at *"mobilizing people affected by NTDs to influence government policy and multi-sectoral programs* was developed and implemented *with a view to improving access to psychosocial, economic and physical rehabilitation services* ." The development of this strategy was done in a participatory manner under the leadership of people with disabilities.

The strategy implementation project called "dignity project" was carried out in the Gbêkê health region in Côte d'Ivoire over the period from December 2022 to November 2023.

Four main axes have been defined in the strategy. These are :

**Axis 1** : Advocacy and awareness raising for improving access to physical, psychosocial, educational and economic rehabilitation services comprising twenty-nine (29) activities;

**Axis 2** : Fight against stigmatization and promotion of respect for the rights of people with disabilities with nine (09) activities;

**Axis 3** : Promotion of mutual aid between PSH-MTN which totals five (05) activities;

**Axis 4** : Strengthening the organizational capacities of the FAHCI association with a view to the sustainability of the actions of PSH-MTN, the number of activities of which amounts to six (06).

A mid-term evaluation of the "Dignity Project" took place in April 2023. This evaluation noted an average level (62%) of implementation of activities. It identified the strengths and weaknesses of the project and made specific recommendations for achieving the set objectives.

The recommendations below were formulated and submitted to the project team as well as to all stakeholders for the successful implementation of the dignity project.

1. Increase the overall budget allocated to the execution of the project in order to make the implementation team more operational;
2. Provide the project team with substantial logistical resources (vehicles, motorcycles) and a fuel voucher for its travel in the various districts and specific localities;
3. Provide the project team with additional equipment to strengthen it;
4. Review the functioning of the scientific committee in order to improve its collaboration with other stakeholders while prioritizing the sustainability of achievements;
5. Provide the project team with a monthly volunteer support fund;
6. Encourage and motivate support partners to act for the well-being of PSH-MTNs;
7. Begin supporting PSH-MTNs by using their respective associations as support;
8. Include in the project implementation process the visit of each program to the project beneficiaries in order to better assess the achievement of the objectives.

In short, the final evaluation had a dual objective: to measure the effectiveness of the project in its planning and execution and to analyze the relevance, effectiveness, sustainability and effects of the project on the beneficiaries' experiences.

This evaluation was based on a qualitative approach using the following data collection tools: documentary review, individual semi-structured interviews and group interviews.

At the end of the evaluation, it was noted that 83.7% of the activities were completed. This proportion, considered very satisfactory, was also appreciated by each stakeholder. The following observations were made according to the specific objectives of the evaluation:

1. *Measure effectiveness in planning and executing project activities* ;

Overall, the project's effectiveness is considered very satisfactory as more than 80% of the activities were completed. However, 10% of the activities could not be completed due to the budget and time constraints.

1. *Analyze relevance, effects and sustainability* *of the project* ;

The project is considered relevant because it took into account the specific needs of people affected by NTDs and took into account the orientations of institutions and programs working in the fight against NTDs and its socio-economic consequences.

Furthermore, the project had positive effects on people affected by NTDs through the following results:

- Improving knowledge (symptoms of NTDs, good practices);
- Improving attitudes (reducing self-stigma, self-confidence, raising hope, bringing people together and working together;
- The associations have developed skills in conducting awareness-raising and advocacy activities in favor of PSH-MTN;
- The PSH-MTN have fully taken ownership of the project and now influence their environment;
- Many steps are underway to improve the daily lives of those affected through advocacy by associations with the relevant authorities and institutions in terms of financial accessibility to health care through access to CMU, access to economic opportunities, access to education and professional training, and the fight against discrimination and stigma.

1. *Analyze the sustainability of the project's actions in the Gbêkê region and the implementation hypotheses* ;

The project approach guarantees the sustainability of the actions:

- The implementation of activities entrusted to existing associations of people affected by NTDs;
- The involvement of all NTD control programs at national and local levels;
- The involvement of public and private institutions whose mandate includes addressing the socio-economic needs of PSH.
- The involvement of administrative authorities and local community leaders.

However, it is appropriate to support the project over a reasonable period of at least three (03) years with more logistical means so that the advocacy actions have perceptible effects in the short, medium and long term in the daily lives of the people affected.

1. *Identify lessons learned and good practices from the project* ;

In terms of lessons learned and good practices, the following points are worth highlighting:

- The participatory and integrated approach made it possible to achieve a successful experience;
- Putting the associations at the heart of the project's implementation has developed their skills in project management and advocacy;
- The improvement of the project implementation monitoring and evaluation plan through the adoption of new strategies made it possible to categorize the results to be achieved in the immediate, medium and long term.
- The institutions responsible for the health, psychological, socio-economic and educational care of vulnerable people have been contacted by the associations to exercise their function and extend their programs to people affected by NTDs;
- The project's actions facilitated the activities of the health districts (awareness raising, detection of new cases (discovery of diseases said to be eradicated in the health region such as yaws);
- Such a pilot project should run over a much longer period (at least three years) to ensure that medium-term objectives are achieved and lessons learned for scaling up can be learned.

Taking into account all these observations, the stakeholders made the following proposals to guarantee the effects of the project in the medium and long term:

***At the level of associations of people affected by NTDs:***

1. Continue advocacy with institutions as part of their activities to include people with mental health conditions (MSMTN) so that they can effectively mobilize resources dedicated to people affected by MTNs;
2. Establish a technical monitoring and evaluation unit to strengthen coordination between the project team and FAHCI management;
3. Seek technical and financial partnerships in order to provide the FAHCI project team with substantial resources to continue project activities;
4. Advocate with institutions and programs to participate in strategy development, planning and awareness-raising activities to take into account the expectations of PSH-MTNs;
5. Maintain collaboration with health and psychological care structures and programs;

***At the level of health and psychological care structures and programs:***

1. Provide technical and financial support to FAHCI and other PSH associations;
2. Involve PSH associations in routine activities to combat NTDs;
3. Make effective free care and psychological support, in particular targeted free care for people affected by NTDs;

***At the level of the socio-economic and educational support structures and programs identified:***

1. Train people affected by NTDs in jobs and employment strategies in the public and private sectors, including self-employment ;
2. Mobilize funds to support people affected by NTDs in the development of Income Generating Activities;
3. Train PSH-MTNs on texts relating to their protection and promotion;
4. Promote access to basic education for people affected by NTDs;

***At the level of technical and financial partners (including the project funder)*** :

1. Provide technical and financial support to the dignity project over an additional three-year period to enable the achievement of medium-term objectives;
2. Supporting strategy scaling initiatives;
3. Provide technical and financial support for periodic monitoring and evaluations to better document successes, challenges and lessons learned.

# **I- Introduction**

## I.1. Presentation of the project context

Disability is historically linked to occult beliefs, community and cultural imaginaries in Côte d'Ivoire. As such, it remains a source of stigma and significant exclusion from access to education, the labor market, and health. Several initiatives aim to improve the daily lives of people with disabilities, but systematic discrimination persists. Although certain policies and regulations have been developed to support the situation of people with disabilities in the country, this situation is not taken into account in the lives and priorities of people living with disabilities [DIDR-OFPRA Report: People with Disabilities - Côte d'Ivoire 2018].

Côte d'Ivoire, a sub-Saharan African country, is primarily affected by multiple NTDs. The National Health Development Plan (NHDP) 2016-2020 indicates that the country is endemic for ten NTDs, namely onchocerciasis, lymphatic filariasis (LF), soil-transmitted helminthiasis (ST), trachoma, schistosomiasis, leprosy, Buruli ulcer, yaws, human African trypanosomiasis (HAT), and finally Guinea worm, which has been declared eradicated.

Current Mass Drug Administration (MDA) programs for onchocerciasis, LF, soil-transmitted helminthiasis, and schistosomiasis do not include a significant morbidity management and disability prevention (MMDP) component alongside chemoprophylaxis. Unfortunately, individuals affected by NTDs discovered during MDAs do not have access to case management services and are thus at risk of developing disabilities.

For NTDs classified as case-managed NTDs such as leprosy, Buruli ulcer, and yaws, the program continues to report a significant number of cases with disabilities already developed in the late stages of the disease. For leprosy, over the past three years, the program has reported approximately 20% of new cases with grade 2 disabilities [Annual Report 2019]. For Buruli ulcer, 26% of cases were reported with category 3 lesions that resulted in permanent disabilities [Annual Report 2018]. Currently, the Bouaké Nord-Ouest health district has just discovered cases of yaws this year during its early detection campaigns in its health area.

While the Federation of Associations of the Disabled of Côte d'Ivoire (FAHCI) has a large database of people with disabilities, the exact number of people with disabilities due to NTDs is not properly documented. Mapping of active cases is still ongoing.

This report constitutes the result of the final evaluation of the dignity pilot project, the objectives of which were to identify PVMTN and their socio-economic needs, to bring specific associations to form partnerships with a view to influencing their care on the educational, socio-professional and health levels.

## I.2. Scientific justification/rationale for the study

The above analysis gives an idea that a good number of people affected by NTDs live with permanent disabilities. The health system alone cannot manage the socio-economic consequences on the person affected by the disease. The reason is that this requires a very strong coordination mechanism involving several sectors of human development.

Although there are a wide variety of studies on the psychosocial and economic reintegration of people with disabilities in general in various fields (WHO, 2019), in the case of NTDs and Buruli ulcer, certain personal and psychosocial determinants and factors have been little studied or remain little taken into account, and health programs have difficulty reaching the poorest populations ( Ndongo et al., 2014).

In this regard, Valérie Simonet (2014) identified in her study five main obstacles encountered by people with disabilities in terms of healthcare. These are the lack of financial resources, inadequate responses from healthcare structures, the lack of rehabilitation structures and skills, the poor organization of people with disabilities and the lack of information and awareness.

Hypothesizing that engaging people with disabilities through NTDs and organizing people with disabilities will sustainably improve the effectiveness and responsiveness of the Côte d'Ivoire government and programs to the needs of people with disabilities through NTDs.

The project aimed to explore effective strategies and mechanisms for mobilizing people affected by NTDs that cause disabilities and their caregivers to influence government policies and multi-sectoral programs to improve and integrate the support system for people disabled by NTDs in Côte d'Ivoire.

## I.3. Implementation

The implementation of the project effectively began in October 2021 with the baseline assessment, followed by the development of the strategy focused on people with disabilities by NTDs to increase their influence on access to care services. The said strategy is in being implemented through the dignity pilot project in the Gbêkê health region, which began in December 2022 and is nearing completion. This project is initiated by the National Leprosy Elimination Program (PNEL) with the financial and technical support of effect : hope Canada, The Leprosy Research Initiative and COPTIMENT.

This report describes the objectives, methodology, results of the final evaluation of the said pilot project and the recommendations made.

# **II- Objectives of the research**

## II.1. General Objective

This study aims to evaluate the implementation of the dignity pilot project

## II.2. Specific Objectives :

1. Measure effectiveness in planning and executing project activities;

2. Analyze the relevance and effects of the project and their sustainability;

3. Analyze the effects before and after the implementation of the strategy developed by FAHCI and their sustainability in the Gbêkê region;

4. Identify lessons learned and good practices from the project;

5. Analyze the project scaling assumptions.

# **III- Methodology**

## III.1 Method and tools

The final baseline assessment was implemented using two strategies:

- The documentary review ;
- Qualitative research ;

The two strategies are as follows:

- Documentary review: this consisted of collecting and consulting methodological documents and study reports relating to the dignity project.
- Qualitative survey: it resulted in the interview of the project stakeholders to collect their contribution and positions on the implementation of the project, the strengths and weaknesses as well as the possible effects on the beneficiaries and their sustainability,

**III.1-1 The documentary review**

This involved the collection and consultation of methodological documents and study reports relating to our subject.

This review was carried out in the following stages:

- Identification and selection of information sources;
- Collection, evaluation of the quality of documents and selection of the most reliable;
- Exploitation of documents using a reading grid.

In addition, the review collected the following documents, although not exhaustive:

- laws, policies, systems and mechanisms that aim to support people with disabilities, including those affected by NTDs;
- Secondary data sources in project documents, key national and regional documents, including relevant policies and plans, data extracted from the health management information system and lessons learned from similar studies conducted in other countries;
- Study reports on KAP (Knowledge, Attitudes and Practices) related to NTDs and other diseases;
- Existing mechanisms for psychosocial and economic support for people with disabilities caused by NTDs,
- The study reports, activity reports and databases of the Dignity pilot project.

Subsequently, a review of these secondary data sources was carried out in order to provide adequate direction to the study in terms of defining key indicators , developing the study protocol and data collection tools.

### **III.1-2 The qualitative survey**

This survey used two main techniques for data collection. These are semi-structured interviews and focus groups. Thus, different interviews were conducted with project stakeholders, administrative authorities, regional directorates and beneficiaries to gather their opinions, contributions and suggestions on the implementation of the project as well as the strengths and weaknesses to measure the effectiveness of the project implementation.

It should also be noted that the gender aspect was taken into account through the inclusion and effective participation of both men and women in the interviews with key informants.

***Inclusion criteria*** : Have been in this position or in this institution for at least six months and have participated in/been informed of the implementation of the Dignity pilot project

***Collection tools:*** the interviews were conducted using a semi-structured interview guide covering the following points:

| **Actors met** | **District/department/directorate** | **Effective** |
| --- | --- | --- |
| Focal point | Bouaké North-West District | 1 |
| CSAS | Bouaké North-West District | 1 |
| Secretary General 1 | Regional prefecture | 1 |
| District Director | Béoumi Health District | 1 |
| Focal point | Botro District | 1 |
| Radio Host | RTI Bouaké | 1 |
| SG DREN 1 | DRENA Bouaké 1 | 1 |
| Regional Director | Regional Directorate of Social Protection | 2 |
| Association Managers (Focus) | FAHCI | 8 |
| Coordinator | FAHCI | 1 |
| Responsible | Coptiment | 1 |
| District Director | Botro Health District | 1 |
| Focal Point | Akawa village (Bouaké) | 1 |
| Agency Manager | Coopec | 1 |
| Buruli ulcer (Focus) | Akawa village | 8 |
| Regional Director | Labor inspection | 1 |
| PV leprosy (Focus) | Sakassou Health District | 8 |
| Focal Point | Sakassou Health District | 1 |
| Focal Point | Gbêkê Health Region | 1 |
| Director | Legal Clinic | 1 |
| Regional Director | Youth employment | 1 |
| Responsible | PNLMTN-CP | 1 |
| Member of the scientific committee | Scientific Committee | 1 |
| Responsible | PNEL Focal Point | 1 |
| UB Coordinator | Vatelot Center | 1 |
| Regional President | CNJCI (community leader) | 1 |
| President | Women of Bouaké (community leader) | 1 |
| President | Coordination of associations for the disabled in the Bandama Valley | 1 |
| Focal Point (Dermatologist) | Bouaké South Health District | 1 |
| Director | Sociocultural Department of the Town Hall | 1 |
| Secretary General | Albino Association | 1 |
| Head of Communities | Chieftaincy (Community Leader) | 1 |
| President | Scientific Committee | 1 |
| Executive Secretary | Caritas | 1 |
| **Total workforce** |  | **56** |

In total, thirty-one (31) individual interviews and three (03) focus groups of eight (08) people each were conducted. The total number of people interviewed was fifty-six (56). For more details, see the table below showing the structures/people actually interviewed:

**Table I** : List of structures surveyed

**Source** : field data, CESI, November 2023

The themes covered in the study include:

- Level of knowledge of implementing actors (FAHCI);
- Level of satisfaction of stakeholders with regard to the implementation of the dignity pilot project;
- Changes brought about by the project (level of ownership, capacity to influence, capacity for advocacy, etc.);
- Effectiveness of the pilot project;
- Socioeconomic needs of targeted PSH-MTN ;
- Experiences of targeted PSH-MTNs in relation to existing policies, regulations and support services;
- New knowledge, attitudes and practices of PSH-MTNs and their caregivers regarding MTNs, existing policies, regulations and support services
- Main obstacles encountered by PSH-MTN in the implementation of the dignity project **;**
- Proposals for solutions relating to the conditions of people with disabilities due to NTDs, etc.
- Project scaling assumptions
- Lessons learned and good practices from the pilot project
- Sustainability and modalities for scaling up the strategy
- Strategies for preserving acquired skills
- Possibility of implementing the project on a national scale.

The interviews were recorded using a Dictaphone, and others were noted down and subsequently transcribed.

It was also a question of appreciating:

The level of achievement of the project objectives;

The level of achievement of results in terms of influence of PSH exerted on the institutions in charge to ensure that their needs are taken into account in relation to the objectives according to the different axes of the project which are:

- - AXIS 1: Advocacy and awareness raising for improving access to physical, psychosocial, educational and economic rehabilitation services;
  - AXIS 2: Fight against stigmatization and promotion of respect for the rights of people with disabilities;
  - AXIS3: Promotion of mutual aid between PSH-MTN;
  - AXIS 4: Strengthening the organizational capacities of the FAHCI association with a view to the sustainability of PSH-MTN actions.

Changes made by the project:

- Capacity of organizations of people with disabilities caused by NTDs to be influential and to carry out advocacy with public and private institutions in order to mobilize partnerships and various resources allowing the effective implementation of existing provisions and especially to create them to effectively influence policies, laws, directives and programs.

In terms of relevance:

- alignment of the project with national strategies for promoting the rights and services of people with disabilities through NTDs;

- the coherence between the strategies developed by the project and the objectives set at the outset; - the project's consideration of cross-cutting issues such as gender.

In terms of efficiency:

- Achievement of objectives in terms of capacity building of organizations of people with disabilities caused by NTDs (FAHCI) in order to conduct awareness-raising, advocate and mobilize partners and various resources;

- Achievement of objectives in terms of appropriation by these organizations of the strategy developed by the project.

In terms of efficiency:

- the capacity with which the objectives set by the project were achieved with the limited resources (budget, time, HR, materials, logistics) made available to the FAHCI;

In terms of impact:

- improving the knowledge of those involved in implementing the project (FAHCI members and others involved) on the various themes covered;

- the change in behavior of these organizations and their members in addressing the problems of people disabled by NTDs.

In terms of sustainability:

- the capacity with which organizations now take charge of the problem of NTDs outside of project actions;

- the local institutional environment and local partnerships favorable to collaboration with FAHCI and other organizations of people with disabilities caused by NTDs.

### **III.1-3 Assessment area and targets**

The final evaluation of the Dignity Pilot Project took place in the Gbêkê health region in central Côte d'Ivoire, which is co -endemic for the targeted diseases and is the implementation area of the project. This region continues to report a significant number of active cases and associated disabilities. Thus, this evaluation study targeted the project implementation team, partners, administrative authorities who facilitated its execution and people affected by Neglected Tropical Diseases ( see Table I).

## III.2 Data collection

The collection was carried out over seven (07) days by four (04) investigators. The collection agents traveled by motorcycle and car. These facilitators (data collection agents) were tasked with conducting the interviews according to the instructions given and ethical considerations , namely informed consent, respect for anonymity and confidentiality . They worked under the supervision and direction of CESI consultants.

In practice, the collection mission took place from Thursday, November 16 to Wednesday, November 22, 2023, in the departments of the actors involved in the implementation of the pilot project, both present and absent from Bouaké (for more details, see the list above). For residents, the meetings were conducted in person, while non-residents were interviewed online, either by zoom or by telephone.

Monitoring the progress of the fundraising effort made it possible to measure the teams' performance, find solutions to any difficulties, and ensure that progress was on schedule. To achieve this, the consultants carried out:

- **A daily debriefing** with all teams via a WhatsApp platform that was created for the occasion. During this debriefing, each team did the following:
- The point of the people investigated by his team;
- The difficulties encountered by his team and the solutions provided;
- The agenda for the next day.
- **A regular collection report** is regularly sent to the steering committee and presents an update on the progress of the collection by district and by locality.
- **A final collection report** presented the data collected by district.
- **An online follow-up meeting took place on D1, D3, D5, etc.**

## III.3 Data processing and report writing

The interviews were recorded, transcribed and entered into Word software. A codebook was then developed to take into account the different themes of the research topic. An analysis of the interviews was carried out through immersion in the data (rereading of verbatim).

The data were analyzed through a content analysis of the speech delivered by the participants during the interviews, illustrating the key messages with one or two verbatim statements that reflect the perceptions expressed by the participants. The thematic and content analysis was carried out with a view to producing the study report.

The effectiveness of the project was assessed through the level of execution of activities as follows:

- Calculation of the activity execution rate by dividing the number of activities carried out or in progress by the total number of activities planned in the logical framework.

We used the same procedure to obtain the different proportions of the level of execution of activities by axis.

- Assessment of the rate of execution of activities according to the scale presented in the table below:

**Table II** : Scale for assessing the level of execution of project activities

| **No.** | **Proportion slice** | **Level of execution of activities** | **Assessment of the level of execution** |
| --- | --- | --- | --- |
|  | 0% to 20% | Very little activity performed | Not at all satisfactory |
|  | 21 to 40% | Activity poorly executed | Not satisfactory |
|  | 41 to 60% | Average activity performed | Unsatisfactory |
|  | 61 to 80% | Activity sufficiently performed | Satisfying |
|  | 81 to 100% | Activity performed | Very satisfactory |

**Source** : our methodology

The study report was drafted and presented to the client. The client's observations and recommendations were incorporated into the report to finalize it. A workshop to validate the report was organized by the Scientific Committee.

# **IV- Results of the final evaluation of the pilot project**

## IV.1 Brief presentation of the project

The project being evaluated took place in two phases:

- A research phase on the needs of people affected by NTDs;
- A formative phase of development and implementation of the Dignity pilot project.

The research phase

**This first phase** consisted of conducting a baseline study to identify the needs of people with disabilities from NTDs. This phase took place from October 2021 to July 2022.

This baseline study identified the specific needs of people with disabilities due to Neglected Tropical Diseases (NTDs). The report of this study provided a general overview of the needs, knowledge, attitudes, practices and beliefs (KAPC) of affected people, their caregivers and relevant key informants in responding to the needs of NTDs.

The following main needs have been identified among people with disabilities due to NTDs:

- Health care, rehabilitation, readaptation and psychosocial support;
- Information – awareness raising on NTDs and the rights of PWDs;
- Schooling and professional training of PSH-MTN;
- Socio-professional integration and support for self-employment;
- Income Generating Activities (IGA) and income support.

Furthermore, this basic study highlighted the following CAPCs:

- Low knowledge of PSH-MTNs on the causes and modes of transmission of MTNs;
- Poor knowledge of PSH-MTN on specialized care structures and rehabilitation/readaptation services;
- Low knowledge of PSH-MTN on laws and regulations protecting people with disabilities;
- Negative attitudes of PSH-MTN towards barriers, prejudices about disability and MTNs, stigma and social discrimination;
- Lack of enabling policies and environment to address barriers, prejudices about disability and NTDs, stigma and social discrimination;
- Low level of organization of PSH-MTNs and low capacity for advocacy and empowerment of PSH-MTN Organizations.

The formative phase

Using the results of the baseline study, **the second phase** consisted of developing a strategy aimed at *"mobilizing people affected by NTDs to influence government policy and multi-sectoral programs for better access to psychosocial, economic and physical rehabilitation services* . "

The development of this strategy was carried out in a participatory manner under the leadership of people with disabilities.

The strategy implementation project called "dignity project" was carried out in the Gbêkê health region in Ivory Coast over the period from December 2022 to November 2023.

Four main axes have been defined in the strategy :

**Axis 1** : Advocacy and awareness raising for improving access to physical, psychosocial, educational and economic rehabilitation services comprising twenty-nine (29) activities;

**Axis 2** : Fight against stigmatization and promotion of respect for the rights of people with disabilities with nine (09) activities;

**Axis 3** : Promotion of mutual aid between PSH-MTN which totals five (05) activities;

**Axis 4** : Strengthening the organizational capacities of the FAHCI association with a view to the sustainability of the actions of PSH-MTN, the number of activities of which amounts to six (06).

## IV.2 Analysis of the relevance of the project (vs. global and specific environment)

The relevance of this project is seen through the methodology of developing its strategy, planning and execution.

Indeed, the strategy adopted in this project was developed by the beneficiaries themselves, supported by health authorities mainly through the various NTD control programs at the national level. These have contributed enormously to the management and governance of the largest NTD control programs in Côte d'Ivoire.

Furthermore, this project strategy was developed following the baseline study which highlighted the specific needs of people affected by NTDs. It is on the scientific basis of this study and its results that the different axes of the strategy were defined. After the mid-term evaluation, the project team corrected its limitations by improving the strategies and especially by carrying out the activities that remained pending.

Regarding the planning and execution of the project, let us specify that health projects are based on three major axes: training, research and awareness-raising or communication. The project aimed at the well-being of PSH-NTDs includes these general elements in the implementation process. To succeed in this process, all stakeholders have pooled their efforts through engagement actions with the involvement of various national programs for the improvement of the living and health conditions of people with disabilities due to NTDs. Consequently, the importance of this project is quite visible in the construction and implementation of this value chain composed of all the links ranging increasingly from caregivers, namely CHWs, to program managers and the scientific committee. Such a system has made it possible to identify PSH-NTDs, their socioeconomic needs and, above all, to develop strategies for their health care, their socio-professional and educational integration through advocacy and solicitations for partnerships. To this end, the Scientific Committee describes its level of involvement in these terms: “ ***As the scientific manager of the project, it is first of all the supervision of the project since the project began in Bouaké, the coordination of activities at the level of the dignity project; everything that happens concerning the project. – We must coordinate these activities and then bring together the scientific committee to make the different points, the different assessments*** ”, (Representative of the Scientific Committee, Abidjan, November 2023).

## IV.3 Analysis of project consistency (internal and external)

The integrated Dignity pilot project involved several stakeholders in developing its implementation strategy. This strategy was part of the relevant national framework and action plan established to combat NTDs in Côte d'Ivoire. Thus, the strategy and action plan of the Dignity pilot project are in line with Côte d'Ivoire's strategic orientations in the fight against NTDs. The Scientific Committee highlighted the participatory and inclusive nature of this pilot project with this statement: ***" It's a project that really made it possible to take stock of the different NTDs and their impacts on populations. But what was interesting was that it was a participatory project where the beneficiaries themselves were stakeholders so that we could see the difficulties they were facing. And based on these difficulties, try to bring concrete strengths to their needs*** ," (Scientific Committee Representative, Abidjan, November 2023). In other words, all stakeholders including the beneficiaries were involved in the implementation of the project.

This is why each implementing actor (institutional and administrative entities, PSH-MTN programs and associations, etc.) played very important roles. It is the smooth running of this system that gave the final result quite satisfactory for the whole. Let us add that overall, the objectives of the project were achieved, despite the very limited resources. These include the limited budget, the time allocated to the implementation of the one-year pilot project which did not allow for certain large-scale actions, the limited equipment since the project team did not have mobility equipment, etc.

However, our discussions showed that some essential structures were not truly involved in the implementation of the dignity pilot project. This lot includes the Buruli ulcer control program and Caritas (a Catholic structure that is implementing a similar project for PVMTN in the same health region (Gbêkê)). Caritas' comments clearly justify this argument: " ***we have not had any consultation with the stakeholders of this dignity project*** ," (Caritas representative, Bouaké, November 2023).

## IV-4 Analysis of project effectiveness

### **IV-4-1 Overall level of execution of project activities**

Legend

|  | Activity carried out entirely |
| --- | --- |
|  | Activity partially completed |
|  | Activity not carried out |

| **ACTIVITY INDICATORS** | **EXECUTION LEVEL** | **PROPORTION OF EXECUTION** | **OBSERVATIONS** | **BENEFICIARY OBSERVATIONS** | **SOURCE OF VERIFICATION** |
| --- | --- | --- | --- | --- | --- |
| **ADVOCACY AND AWARENESS RAISING FOR IMPROVED ACCESS TO PHYSICAL, PSYCHOSOCIAL, EDUCATIONAL AND ECONOMIC REHABILITATION SERVICES** | | | | | |
| **COMMITMENT OF ADMINISTRATIVE AUTHORITIES AND COMMUNITY LEADERS TO THE IMPLEMENTATION OF THE STRATEGY** | | | | | |
| Organize a project launch ceremony to raise awareness among administrative authorities and community and association leaders in Bouaké | Made | 100% | Nothing to report | Kick-off meeting held | Launch attendance list |
| Obtaining stakeholder commitment to improving the quality of life of PH-MTN | Made | 100% | Nothing to report | Commitment made at launch | Launch attendance list |
| Organize at least one meeting with each institution to strengthen collaboration | Made | 100% | Nothing to report | Meeting with each institution held | Mail discharge |
| **ADVOCACY FOR FREE CARE INCLUDING REHABILITATION AND REHABILITATION CARE FOR PSH-MTN** | | | | | |
| Submit a request to the Ministry of Health and the Ministry of Social Protection to obtain free coverage for rehabilitation and readaptation care for PSH-MTN | Made | 100% | Nothing to report | Request submitted but unsatisfactory return (insufficient medication) | Lack of justification |
| Follow up on correspondence through at least one meeting with the authorities of the target institutions (Health and social protection). | Made | 100% | Nothing to report | Follow-up carried out | Lack of justification |
| **STRENGTHENING PSYCHOLOGICAL CARE FOR PSH-MTN** | | | | | |
| Organize an advocacy meeting targeting health district authorities so that psychological care is integrated into the care of PSH-MTNs | Made | 100% | Advocacy meeting held without follow-up | Did not receive psychological support | Lack of justification |
| Follow up with the PNEL for the training of health workers for this purpose | Made | 100% | District health workers trained | Insufficient intervention resources | Lack of justification |
| Participate in planning meetings on NTD control in health districts and at central level | Made | 100% | Nothing to report | Planning carried out according to the district program | Attendance list |
| **IMPROVE THE KNOWLEDGE AND ATTITUDES OF POPULATIONS, PWDs AND CAREGIVERS ON NTDs AND LAWS AND REGULATIONS PROTECTING PWDs.** | | | | | |
| Organize a quarterly radio program to raise awareness about NTDs, recognition of suspicious signs, possible complications and management, as well as laws and regulations that protect people with disabilities. | Two radio shows per week instead of one show per quarter | 100% | Nothing to report | Radio broadcasts produced regularly during the week | Lack of justification |
| Distribute videos on WhatsApp and Facebook on raising awareness about NTDs, recognizing suspicious signs, possible complications and management, as well as laws and regulations that protect people with disabilities | Achieved on average | 50% | Many PVMTN do not have a mobile phone (android) with WhatsApp and Facebook applications. | Never received any SMS, WhatsApp and Facebook messages on NTD awareness | Presence of videos |
| Organize advocacy meetings with community leaders to remove cultural barriers and/or improve collaboration with the health system for the management of PSH-MTNs | Made | 100% | Radio awareness and meeting carried out | Meeting at the prefecture but no individual meetings with community leaders | Attendance list |
| Participate in planning meetings on NTD control in health districts | Made | 100% | Nothing to report | Planning meetings and awareness raising conducted | Lack of justification |
| **MOBILIZING RESOURCES FOR THE INTEGRATION OF CHILDREN WITH DISABILITIES DUE TO NTDs INTO THE EDUCATION SYSTEM THROUGH ADVOCACY AND SCHOOL SUPPORT** | | | | | |
| Identify children with disabilities due to NTDs who have schooling needs during the PSH-NTD census with the help of focal points | Made | 100% | Nothing to report | Census taken and nothing more | List of children |
| Organize an advocacy meeting with national education officials for the admission of children in need into schools | Made | 100% | Meetings held but no follow-up | Unorganized advocacy meeting | Attendance list |
| Organize a meeting to mobilize financial resources with financial partners and government aid agencies to obtain support for PSH-MTN. | Accomplished | 100% | Access to credit, Baobab and Coopec met | Meetings completed but need to swallow | Minutes of meetings not received |
| **THE PROMOTION OF SPECIALIZED STRUCTURES IN THE EDUCATION OF PH TO PSH-MTN** | | | | | |
| Identify existing specialized structures in Ivory Coast | Accomplished | 100% | Specialized structures identified | Activity carried out with Botro's DD | List of structures |
| Send awareness-raising letters and requests for admission of PSH-MTNs to the identified structures. | Accomplished | 100% | Letters addressed to specialized structures | Mail received | Presence of mail discharges |
| Organize awareness-raising among PSH of school-age or training age on the existence of specialized education structures and the conditions of access to them | Made | 100% | Nothing to report | Identification made but no concrete action | Lack of justification |
| Have a PH-MTN WhatsApp address book for sharing information | Made | 100% | Nothing to report | PVMTN say they have received nothing | List presence and number |
| **IMPROVING ACCESS TO TRAINING FOR ADULTS WITH DISABILITIES FROM NTDs** | | | | | |
| Produce and distribute leaflets (in digital and physical form) to PSH-MTNs providing information on literacy and vocational training opportunities. | Made | 100% | Nothing to report | Prospectus received by certain PVMTN | Presence of leaflets |
| Organize an awareness-raising meeting for education system officials on the application of laws relating to access to literacy and vocational training for people with disabilities | Activity not carried out | 0% | Discussion with DREN 1 officials | Unorganized awareness meeting | Nothing to report |
| **STRENGTHENING THE FINANCIAL CAPACITY (EMPLOYMENT) OF PSH-MTN AND CAREGIVERS THROUGH PERIODIC CASH TRANSFERS, THE DEVELOPMENT OF AGR AND THE PROMOTION OF SELF-EMPLOYMENT** | | | | | |
| Identify partners for financial and technical support for people with disabilities | Made | 100% | Nothing to report | No support received | Lack of justification |
| Establish collaboration agreements with two institutions specializing in the promotion of self-employment | Made | 100% | Nothing to report |  | Lack of justification |
| Establish a fund to guarantee access to microcredit for PSH-MTN | Activity not carried out | 0% | Lack of substance | No access to any Fund | Nothing to report |
| Identify the needs in AGR for PSH-MTN | Made | 100% | Nothing to report | Needs identified but not followed up | Presence of the list of needs |
| Identify PSH-MTNs who should benefit from emergency cash transfers | Made | 100% | Nothing to report | Promise made but no follow-up | Presence of list of PSH-MTN |
| Advocate with institutions responsible for monetary transfers to take into account PSH-MTNs | Made | 100% | Advocacy completed | No swallow obtained | Presence of mail discharge |
| Establish an intersectoral monitoring committee | Made | 100% | Nothing to report |  | No justification |
| Organize an advocacy meeting with the labor inspectorate so that public and private employers apply the laws in favor of the employment of PSH-MTN | Made | 100% | The Labor Inspectorate has promised to make texts available | The labor inspectorate recognizes a single exchange meeting | Attendance list presence |
| **FIGHT AGAINST STIGMA AND PROMOTION OF RESPECT FOR THE RIGHTS OF PEOPLE WITH DISABILITIES** | | | | | |
| **IMPROVE THE KNOWLEDGE OF THE POPULATION, INCLUDING PSH-MTN AND CAREGIVERS, ON LAWS AND REGULATIONS PROTECTING PEOPLE WITH DISABILITIES.** | | | | | |
| Organize awareness sessions through community radio stations (integrated into awareness activities) | Made | 100% | Radio awareness sessions carried out | Radio awareness sessions carried out | Testimonies from PVMTN and Bouaké radio |
| Produce and distribute 500 awareness posters on laws and regulations protecting people with disabilities, specialized care structures and rehabilitation/readaptation services. | Not carried out to standards | 0% | Activity taken into account during radio awareness raising | Awareness radios carried out | List of themes developed |
| **PROMOTING THE RIGHTS OF PSH-MTN** | | | | | |
| Develop and distribute via WhatsApp to PSH-MTN and their helpers a video that presents the rights of people with disabilities. | Made | 100% | The majority received the messages | Messages forwarded to those with Android phones | List of PSH-MTN and their number |
| Organize an advocacy meeting with the authorities in charge of territorial administration and the Regional Directors of the target ministries to raise awareness of the laws and regulations that protect people with disabilities | Made | 100% | Meeting held with relevant authorities | Meeting held | Attendance list |
| Establish a partnership with the Bouaké legal clinic for the management of denials of rights of PSH-MTN | Made | 100% | Meeting with the legal clinic | Process in progress (unfinished) | Attendance list |
| Organize an advocacy meeting with the Ministry of Justice in Bouaké to obtain its support for the promotion of the rights of PSH-MTNs Make an appointment and meet | Made | 100% | Judicial authorities met | Meeting held | Attendance list |
| **STRENGTHENING THE FIGHT AGAINST STIGMA AND SOCIAL DISCRIMINATION AND PROMOTING THE INCLUSION AND PARTICIPATION OF PSH-NTDs** | | | | | |
| List and disseminate existing laws and regulations protecting PSH | Made | 100% | Nothing to report | The associations confirm having received these laws and regulations. | No justification |
| List the stigmatizing practices experienced in the project area by PSH-MTNs | Made | 100% | Nothing to report | Listed practices | Activity report |
| Organize 5 awareness-raising meetings for community leaders on the abandonment of stigmatizing practices by community members | Partially completed | 20% | Only one meeting held out of 5 | Only one meeting held | Attendance list |
| **PROMOTION OF MUTUAL AID BETWEEN PSH-MTN** | | | | | |
| **STRENGTHENING THE SELF-HELP CAPACITIES OF PSH-MTN** | | | | | |
| Train a PSH-MTN/FAHCI pool and caregivers in the psychological care of PSH-MTN – Psychological care module to be administered by HP de Bouaké to the participant in half a day | Not realized | 0% | We interacted with district focal points | Nothing to report | Nothing to report |
| Establish a support team for the preparation of admission files and referral of PSH-MTNs to specialized structures | Completed (10 volunteers) | 100% | Nothing to report | Team formed | List of ten (10) volunteers |
| Train a pool of 25 people on NTDs to raise awareness on the recognition of suspicious signs, possible complications and management, as well as the laws and regulations that protect people with disabilities (Training of focal points) | Partially completed | 25% | Difficult access to PVMTN and lack of mobility equipment |  | No justification |
| Organize training for a pool of PSH-MTNs in the promotion and defense of PSH rights. (Training of focal points) | Made | 100% | Training of focal points carried out |  | Attendance list |
| Organize a meeting to promote self-help groups with PH-MTNs and their caregivers (Send messages via WhatsApp – identify a mutual aid focal point) | Made | 100% | Actions through PSH associations |  |  |
| **STRENGTHENING THE ORGANIZATIONAL CAPACITIES OF THE FAHCI ASSOCIATION WITH A VIEW TO THE SUSTAINABILITY OF PSH-MTN ACTIONS** | | | | | |
| **STRENGTHENING ASSOCIATIVE MANAGEMENT AND RESOURCE MOBILIZATION CAPACITIES TO SUSTAIN PH-MTN ACTIONS** | | | | | |
| Organize quarterly exchange meetings with the TFPs with a view to improving the administrative, financial and programmatic management of the association | Made | 100% | Online training |  | Lack of justification |
| Partner with a firm to develop and implement a resource mobilization plan to support the execution of the plan | Not yet completed | 0% | Nothing to report | Nothing to report | Nothing to report |
| **ENSURE THE COORDINATION, MONITORING AND EVALUATION OF THE PROJECT** | | | | | |
| Identify focal points at each district level for the implementation of the project | Made | 100% | Nothing to report | Focal points identified | List of focal points |
| Develop and implement a project monitoring and evaluation plan | Completed (bi-monthly monitoring and evaluation) | 100% | Nothing to report | Project team | Team List |
| Equip the coordination unit with IT equipment | Accomplished | 100% | Nothing to report | Printer, computer and refills | List or photograph of equipment |
| Organize monthly project monitoring meetings with stakeholders | Made | 100% | Nothing to report | Monthly meetings held | Attendance list |

Source: CESI survey data, November 2023

The level of execution of the activities was assessed as follows:

- In total, 41 activities were fully completed out of 49 activities, representing an execution rate of 83.7%, which corresponds to a very satisfactory level of execution (above 80%) according to the assessment matrix that we gave ourselves;
- Only 3 activities were partially completed out of 49 activities, a rate of 6.1%;
- **However, 5 activities could not be carried out out of 49 activities, i.e. a rate of 10.2%** .

Overall, the overall level of implementation of the pilot project activities is very satisfactory (83.7%) as evidenced by the respective comments of the Scientific Committee, Coptiment and FAHCI:

" ***In any case, overall, I am satisfied with the project. But I think that perhaps, since it is a pilot project, we should seek more funding to be able to expand the project further*** ," (Representative of the Scientific Committee, Abidjan, November 2023)

" ***I am really satisfied, moreover, on several counts. First, I would say that I am satisfied because the national leprosy elimination program and in general the NTD program - it is the leprosy program that carries it, because the other programs like Buruli ulcer and NTDs in preventive chemotherapy have really seen the merits of such an approach ." (*** Coptiment representative , Abidjan, November 2023)

" ***I am very satisfied with the project and my hope is that it continues. We must not start and leave it, that is not really good*** ," ( FAHCI Representative, Bouaké, November 2023).

### **IV.4.2 Level of achievement of project objectives**

The effectiveness of the project is observed in the planning and execution, which is broken down into four specific objectives. Each specific objective includes activities whose successful execution has made it possible to understand the effectiveness of the project. Thus, firstly, we will present the activities of each specific objective, and secondly, we will analyze the level of satisfaction of the stakeholders at several levels.

***OS1- advocacy and awareness raising for improving access to physical, psychosocial, educational and economic rehabilitation services***

At the level of this first specific objective, out of 29 planned activities, 26 were carried out, representing a rate of 89.7%. This proportion shows a very satisfactory level of achievement of OS1. This performance is explained by the good collaboration that prevailed between the actors implementing the project. Added to this effort is the dynamism of the project team, which agreed to work with passion and tirelessly. To describe the scale of the work, the Botro Health District puts forward this: " ***we had to raise awareness among the population concerned about NTDs. There are some that are due to many things, whether it is water, so you have to drink clean water. You have to avoid letting children swim in dirty water to avoid getting Buruli ulcer , or stories of yaws. In any case, we talked about at least four NTDs. We finished the tour yesterday*** », (Health District Representative, Botro, November 2023).

We note from OS1 that the immediate effect has been achieved, and therefore observable to the extent that advocacy has been carried out and awareness-raising has been done. Consequently, the issue of NTDs and especially the situation of people affected by NTDs is known both in the specific communities and by the structures contacted. Furthermore, advocacy has been carried out with a view to extending free care to rehabilitation and re-adaptation services for PSH-NTDs. In addition, the targeted national and regional health authorities have made a commitment to contribute to the improvement of psychological care and to integrate PSH-NTDs into the care provided.

However, the actual delivery of these services (free care, improved access to physical, psychosocial, educational and economic rehabilitation services) is not the responsibility of the Dignity pilot project. It is up to the structures and organizations to which the advocacy has been directed to make arrangements for the effective delivery of these services in the medium and long term. This is justified by the fact that the delivery of these services requires a fairly long period of time and administrative and budgetary procedures to be put in place.

***OS2 - fight against stigmatization and promote respect for the rights of people with disabilities***

This specific objective is broken down into nine (09) activities, seven (07) of which were carried out, representing an achievement rate of 77.8%. The fight against stigma was carried out through meetings with community leaders, radio awareness sessions, and the sharing of leaflets. This activity involved the leaders of disabled people's associations. This rate of 77.8% shows that the short-term indicators have been achieved. This immediately resulted in strengthening the capacities of PWD associations to, in turn, train their members in the fight against self-stigma and develop self-confidence. In this regard, awareness-raising strategies were developed and implemented to improve the knowledge and attitudes of people affected by NTDs (signs, symptoms, complications, Good Practices) in order to better take care of themselves and thus reduce disabilities. From now on, disabled people's associations are able to promote the rights of people affected by NTDs. The medium and long-term effect within the general population in terms of reducing stigmatizing and discriminatory behaviors towards PSHMTN does not depend on the Dignity pilot project.

***OS3- promotion of mutual aid between*** ***PSH-MTN***

To this end, five (5) activities were planned, but only three (03) were fully implemented and one (01) partially implemented. Thus, we have the following completion rates: 60% for final implementation and 20% for partial implementation. This result shows that the promotion of mutual assistance between PSH-NTDs is on the right track. It would be wise to perpetuate this initiative through large-scale actions, especially in rural areas where people affected by NTDs express several socioeconomic needs. In short, we can see from these results of SO3 that the indicator relating to the promotion of mutual assistance between PSH-NTDs is achieved by strengthening the self-help and mutual aid capacities of PSH-NTDs. Self-help mechanisms are now being developed within associations through the identification of the needs of their members and advocacy for the mobilization of resources. However, the medium and long-term effects will be felt when resources are effectively mobilized by the associations and made available to their members.

***OS4- Strengthening the organizational capacities of the FAHCI association with a view to sustaining the actions of PSH-MTN***

Out of six (06) activities planned in this specific objective, five (05) were fully completed, representing an achievement rate of 83.3%. Indeed, the project team (FAHCI) implemented several actions to strengthen the capacities of its members in terms of associative management, coordination, monitoring and evaluation of the project.

Ultimately, we can conclude that the activities of the various specific objectives were carried out at acceptable rates. This demonstrates that the objectives of the pilot project were achieved. This result is justified by the good planning and execution of the project.

However, the activity related to establishing a partnership with a firm for the development and implementation of a resource mobilization plan to support the execution of the plan has not yet been carried out. However, the indicators related to capacity building of the FAHCI component associations have been achieved. Today, each association has the required capacity to develop innovative strategies likely to ensure the sustainability of actions aimed at supporting PSH-MTNs. In addition, the FACI's capacities in association management and resource mobilization for the sustainability of the strategy are strengthened.

This project has generated immediate effects among people affected by NTDs, since it is a source of change and improvement in the living conditions of the beneficiaries. To this end, the Bouaké Northwest Health District, speaking of the effectiveness of the project, puts forward this: ***" It's a project that is effective. You can feel it automatically. Because there are projects when you set them up it takes years, a good while, or it's long term. But the effects are immediate. When we arrive on the ground we provide care, it allows us to detect cases*** ," (Representative of the Bouaké Northwest District, Bouaké, November 2023).

However, the FAHCI noted some flaws in planning and execution. For them, the resources made available were limited, especially with regard to logistics (mobility of stakeholders). Moreover, this is the reason behind the massive recruitment of volunteers, as the following comments relate: " ***planning, for me, there were some flaws in the resources made available to the project. It was the means of transport that were a problem. In terms of execution too, it was good because I myself - at the beginning I was alone, but then I saw that I couldn't since the project covers the entire Bouaké region. So I looked for ten (10) volunteers to help me, including eight (08) with disabilities and two (02) able-bodied people*** ," (FAHCI Representative, Bouaké, November 2023).

For the FAHCI, the results would be more convincing if sufficient resources had been made available to the project team. To reinforce this relative position, the Scientific Committee, although it recognizes the efforts made, notes some shortcomings in planning and execution which can be summed up in the delay recorded which was not made up for before the end of the project: " ***planning, the execution of the project yes indeed good. I think that in the execution it must be said, eh there were still small delays in the execution of the various activities. That must be said eh. And then the project was not extended a little because we still fell behind in certain activities which were very late*** ", (Scientific Committee Representative, Abidjan, November 2023).

## IV.5 Analysis of the effects of the project on People affected by NTDs

The project has generated several effects on PVMTN. These include the reduction of stigma, discrimination, rejection and especially community self-exclusion , after the various awareness-raising phases in the health districts and through radio broadcasts. The advent of this project has also generated hope and confidence in associations of people with disabilities due to NTDs. Moreover, the position of the Botro Health District is part of this perspective. For him, the dignity project has generated " ***hope and then seeing that we are considered, we are taken into account by society* ," (** Health District Representative, Botro, November 2023).

In addition, associations of people with disabilities due to NTDs have understood that they are capable of large-scale actions and, above all, now feel human. Thus, with the help of training and awareness-raising, many have regained confidence in life. Thus, several PVMTN have understood that they are no different from other so-called normal men. Moreover, this is what is perceived in the comments of the legal clinic: " ***I think that the project has generated even more confidence - they were able to see that there is no need to make a difference between a disabled person and a normal person. They understood that the fight must begin with themselves, because there is no better protector than the one we must protect. - It gave much more confidence and allowed them to understand that being in a situation of disability is not an end in itself and that we can accomplish a lot of things like those who call themselves normal*** ," ( Representative of the legal clinic, Bouaké, November 2023).

In addition, the dignity project has improved the daily lives of PVMTN. This is what the RTI Bouaké testifies in these terms: ***“There were also some who were victims of neglected tropical diseases. But who managed to live healthily. But at the beginning it was not easy. So with all these examples it struck me. I saw the smiles on the faces of these people, so it was good, it is a project that is truly commendable*** ”, ( RTI Representative, Bouaké, November 2023).

In short, the indicators related to the immediate impact of the project have been partially achieved. This has resulted in several achievements, including awareness raising on NTDs and especially the warning signs and the first steps to take regarding treatment locations. Today, several PSH-NTDs are being reintegrated into specific communities. The other aspect, which will be achieved later, relates to the mobilization of resources for the well-being of PSH-NTDs.

In such a context it is important to mention some effects of the project on the knowledge, attitudes of PVMTN, and at the socio-health and socio-economic level.

### **IV.5.1 At the level of knowledge and attitudes**

The Dignity pilot project improved the knowledge of PVMTN and promoted significant changes in the attitudes of PVMTN and their respective communities. The project allowed several people to learn about the manifestation of NTDs and especially the first symptoms. These gradual changes are linked to the effects of training and awareness-raising activities carried out in person by the project team and through RTI. In addition, association leaders played a major role in this communication process, as each disseminated the information and training received within their own groups, thus creating a multiplier effect.

Therefore, the project's actions had a significant effect in terms of improving attitudes, which was manifested by the rapprochement and collaboration between PVMTN and so-called normal people. To this end, the associations of PWD (not suffering from NTDs) affirm this: " ***In any case, the project, the training brought me a lot because I didn't really know how these diseases developed. We learned how they are contaminated, how you get them, how you heal. Before, we used to run away, we were afraid of them. But through this project, I learned that these people are the same as us. We don't have to neglect them, we don't have to run away from them, so it's really a good project."*** », Focus representative of PSH association leaders, Bouaké, November 2023).

At this level, the short-term objectives of the project have been achieved to the extent that knowledge of NTDs has improved the attitude of PVMTN and PSH-NTDs towards themselves, and that of the populations towards PVMTN and PSH-NTDs.

### **IV.5.2 At the level of the health situation of PVMTN**

The health effects are not sufficiently perceptible, as PVMTNs still do not have access to free healthcare. The free care that should result from partnerships between their respective associations and the Ministry of Health is not effective in health centers. In this regard, the testimony of the Bouaké Nord-Ouest health district regarding free care for PVMTNs is edifying. It states this: " ***If they have health problems, they go to the center, I think they buy the medication. We have not received information that these people should be treated differently. I do not know if the nurses of Bouaké EST do it. Perhaps, since they know the population, they are doing favors. But to say that we received a note from the hierarchy saying that we must treat these people for free, to my knowledge no."***», (Representative of the Bouaké North-West Health District, Bouaké, November 2023).

However, it is essential to mention that people in general and those affected by NTDs benefit from free care during occasional early detection campaigns, although the medications provided by partners are insufficient. Moreover, this is what the Bouaké Nord-Ouest health district describes in the following comments: " ***The partner provides us with the medications; but it is not in sufficient quantity. We have ten villages and when we have to distribute these medications it is not enough. But we know that these populations really do not have the means even if you give them a prescription they will not be able to honor it, it is complicated. Because when we arrive we tell them that we are coming to provide free treatment and they expect everything to be really free. When we say for those who have skin problems, those who have aches and pains and all that, they come* "** (Representative of the Bouaké Nord-Ouest Health District, Bouaké, November 2023).

Although PVMTNs do not have free access to healthcare, the project team is taking steps to ensure that everyone has a CMU card to benefit from lower-cost healthcare services. Through its statement, the FAHCI outlines the route of this process: " ***For the moment, we cannot say that they have free access to healthcare because of the project. But specialized institutions have already been informed, but we cannot say that because of the project they have free access. But at the CMU card level, we have made pleas, and the minister has requested a list so that the CMU card and contributions are free for people with disabilities. So we have sent the list and we are waiting*** ," (FAHCI representative, Bouaké, November 2023).

In short, the project has not directly impacted the health aspect of PVMTN, but the future looks bright for those registered on the list of future beneficiaries of the CMU card whose contributions will be covered by specialized institutions, especially social protection. However, the indicators related to the effective and free care of PVMTN will be achieved in the medium and long term, just the time it takes for the advocacy made to the health authorities to have an effect.

### **IV.5.3 At the level of the socio-economic situation of PVMTN**

At the socioeconomic level, it is important to clarify that the pilot project does not aim to provide socioeconomic assistance to PWD-NTDs and PVMTNs. Rather, it aims to show them the path to follow for their socioeconomic empowerment. This is why PVMTNs and PWD-NTDs continue to experience their ordeal at the socioeconomic level.

Indeed, the disease has left several people disabled, unemployed while keeping them in a precarious life. Lacking real support, they express their impatience to benefit from material or financial support overnight that could contribute to the satisfaction of their social and economic expectations. To meet this vital need, the project team met with several professional and educational structures for the integration of PSH-MTN. The following comments from the FAHCI describe the actions carried out in this direction: " ***The socioeconomic rehabilitation and re-education of these people as I say; that's why I mentioned the timeframe of the pilot phase, it was short. And one year was not enough, since we reached certain specialized institutions, we cannot put pressure on them since they have their address book, their program too to follow. We reached the heads of companies, the directors in any case they welcomed the project. But they have not yet provided us with what is necessary to continue to achieve results*** ,” (FAHCI representative, Bouaké, November 2023).

In addition, the project team had discussions with integration structures such as youth employment, which has several employment opportunities for PSH-MTN, as stated in this statement from the FAHCI: “ ***Training center, professional integration, we have had a lot of discussions with the youth employment agency, which even told us that there are projects for people with disabilities, but they do not see people with disabilities and now there are several who are heading there*** ,” (FAHCI representative, Bouaké, November 2023).

On the socioeconomic level, the objectives will be achieved in the medium and long term because the procedure is ongoing with the contacted structures. Thus, PSH-MTNs will be trained in IGAs and will benefit from technical support for their installation. Thanks to the partnerships that will be established with financial support structures, they will be able to receive funding to develop their activities.

### **IV.5.4 At the level of stakeholder satisfaction**

All stakeholders expressed their satisfaction with the implementation of the dignity pilot project, despite the few shortcomings mentioned. The following comments clearly justify their position:

" ***My greatest satisfaction is that this project involves PWDs and people living with NTDs. Because we say today that if you want to do something for someone and you don't involve them, you won't get good results. And these people are present at all the training sessions.*** », (Representative of the PSH-MTN Association, Bouaké, November 2023.

“ ***We’ll say, well. It’s excellent. In any case, it’s good; it really helped us. We hope it will be repeated*** ,” (Representative of the Bouaké Northwest District, Bouaké, November 2023).

“ ***Overall, I am very satisfied*** ,” (Scientific Committee Representative, Abidjan, November 2023).

- **In relation to the knowledge and attitudes of PVMTN**

The stakeholders' satisfaction with the knowledge and attitudes of the PVMTN results from the comparison they establish between their situation before the project and that of today. This exercise allowed them to understand the effects of radio and face-to-face awareness campaigns carried out by the project team and its partners. This statement from the Bouaké Northwest health district is edifying: " ***When we don't have the information, these people are stigmatized. Because these people are said to have cast a spell on them or it's witchcraft, these people are stigmatized. So when the population is informed about the disease, you see that there is a way of viewing these people. So each time when we went to the villages, we took care to really raise awareness among the population. So all this has meant that it has more or less changed the population's view of these people." So you see that if the way these people look at them changes, you see that the person themselves will feel better*** ,” (Representative of the Bouaké North-West District, Bouaké, November 2023).

The associations believe that the Dignity pilot project has allowed them to better understand the NTDs they suffer from. " ***In this project, we learned how to treat yourself when you have the disease, how you catch the disease, and how to avoid it*** ," (Representative of the PSH-NTD Association, Bouaké, November 2023).

- **Regarding the health situation of PVMTN**

The majority of stakeholders expressed dissatisfaction with the health component, which is not yet a given for the PVMTN. The project team has certainly identified and recorded the PVMTN, but access to free healthcare is not yet a reality. This is the heartfelt cry of the PVMTN of Akawa at the Bouaké Sud health district level. " ***So far, they haven't done anything yet. Because they have simply recorded the PVMTN and notified the needs. A time will come when they will send us what we said. In any case, they only held one meeting; since then, they haven't come back*** ," (PVMTN Representative, Akawa, Bouaké, November 2023).

- **Compared to the socio-economic situation of PVMTN**

Although the pilot project is almost complete, the effects on the socioeconomic situation are not yet felt in the lives of PVMTN. Many complain, suggesting that promises of financial support have been made to them. But so far they have not received any feedback on the promises made. As a result, they are in a dead end. This is, moreover, this reality that this statement relates: " ***here we are when we had the disease now we cannot work we need to be given money yet we cannot find it, that is what tires us out*** ", (PVMTN Representative, Akawa, Bouaké, November 2023).

Several PVMTN are still waiting for the promises made during their registration, as the Botro Health District points out: " ***the sick are waiting for what will come after calling them after registering them, we don't know what happens next. So there is that, since we registered them, we told them to give all their numbers. How to contact them, what they want, all that. So that has already been done. Now we just have to do what we promised them*** ," (Health District Representative, Botro, November 2023). To alleviate the situation of the PVMTN, the project team has asked the Ministry of Solidarity and the Fight against Poverty for support for these vulnerable people, as described by Coptiment : " ***For the mobilization of resources, for example, the FAHCI has already made pleas so that the Ministry of Solidarity and the Fight against Poverty can support them*** ," ( Coptiment Representative , Abidjan, November 2023.

- **Compared to the capacity of appropriation, advocacy and influence of PVMTN**

The Dignity Pilot Project aimed to restore the dignity of PVMTN through several activities. It was also about ***"getting them to believe in themselves, to have confidence in their ability to achieve what those who consider themselves normal can do. To know their rights and to have their rights respected by the various authorities*** " (Leprosy Program Representative, Abidjan, November 2023).

Following the implementation of this package of activities, we observed a enthusiasm among PSH-MTNs to take ownership of the project in order to continue advocating for the improvement of their daily lives.

Indeed, it emerges from the various interviews and focus groups that the Dignity Pilot Project provides an awakening framework for PVMTN. Many have a better understanding of their situation and express a desire to have the minimum necessary to undertake their empowerment. However, it is important to mention that they currently need support and guidance at several levels (health, material, financial, psychological, etc.) since vulnerability due to the disease differs from one person to another. Advocacy actions carried out with socioeconomic and professional structures have not yet borne fruit. However, it is important to emphasize that the determination of PSH-MTN and PVMTN shows that they have already gradually taken ownership of the project. This is evident in their remarkable mobilization at the meetings and especially the constructive proposals they make. This is explained by the fact that they are now aware that entities are mobilizing for their well-being. As a result, they are now motivated and even courageous, and they want to take advantage of this opportunity to emerge from the shadows where stigma, discrimination and self-exclusion had imprisoned them.

### **IV.5.5 At the level of situation analysis Before versus After the project**

The short implementation period of the Dignity pilot project (12 months) did not allow for a quantitative assessment of the project's impact in terms of significant medium- and long-term changes among the final beneficiaries. Therefore, it was not possible to make a comparison between the current situation of people with disabilities due to NTDs in terms of satisfaction of needs and living conditions and the initial situation established during the baseline situation.

However, the final evaluation of the project showed significant qualitative changes that occurred in the short term in the life of the associations of people with disabilities due to NTDs and their members (FAHCI and affiliated associations) responsible for implementing the project (direct beneficiaries).

The table below compares the situation before versus after the Dignity pilot project on the immediate changes made among direct beneficiaries.

| **Strategic axes** | **Situation before the project** | **Situation after the project** |
| --- | --- | --- |
| **Axis 1** : Advocacy and awareness raising for improving access to physical, psychosocial, educational and economic rehabilitation services | Low awareness for improving access to physical, psychosocial, educational and economic rehabilitation services | Engagement in advocacy and awareness raising to improve access to physical, psychosocial, educational and economic rehabilitation services |
| **Axis 2** : Fight against stigmatization and promotion of respect for the rights of people with disabilities | Low level of knowledge and skills of FAHCI and its members in matters of NTDs, care structures and good practices to be observed | Improving knowledge and developing the skills of FAHCI and affiliated associations in conducting awareness-raising and advocacy activities for PSH-MTNs  Advocacy towards dedicated institutions to facilitate access to health, psychosocial, educational and economic care services for their members |
|  | Non-involvement of FAHCI and affiliated associations in initiatives to combat NTDs | Ownership of the Dignity project by the FAHCI and its members both in the implementation of activities and in the coordination of the project including advocacy towards partners |
| **Axis 3** : Promotion of mutual aid between PSH-MTN | Lack of knowledge of NTDs and NTD treatment structures | Improving knowledge on NTDs and treatment structures for NTD cases |
|  | Needs for psychological support and socio-economic rehabilitation | Establishment of a dynamic list of people disabled by NTDs and their needs |
| **Axis 4** : Strengthening the organizational capacities of the FAHCI association with a view to sustaining the actions of PSH-MTN | Weak organizational capacity of FAHCI and affiliated associations, no previous experience in autonomous project management | Organizational capacities of FAHCI and affiliated associations strengthened through training, coaching and especially through autonomous management of the dignity project |
|  | Non-existence or very low level of mobilization of PSH-MTN associations | Integration of the fight against NTDs into the interventions of existing associations of disabled people affiliated to FAHCI |

## IV.6 Analysis of the project's strengths and weaknesses

At the end of the implementation of the dignity pilot project, it is time to identify and analyze the strengths and weaknesses. However, let us specify that this analysis will be done in three sub-points which are:

- Project management bodies,
- Project implementation device,
- Implementation of the project.

In each section, strengths and weaknesses will be highlighted and illustrated with statements from the respective actors.

- ***At the level of the project management bodies***

At the organizational level, one of the project's strengths lies in the dynamism of the project team and the contribution of all stakeholders. Moreover, this is the point of view of the Botro Health District, which believes that: " ***the strengths of this project are that it has staff who are concerned about the future of their patients."*** », (Health District Representative, Botro, November 2023).

The second strength is that all the partners appreciated, welcomed the project and mobilized: " ***the strengths first of all are that the team is dynamic, we must recognize that the team is dynamic, the population welcomes the project, the institutions welcome the project everyone welcomes the project this gives the courage to move forward, everywhere we have been, the advocacy which gives strength to this project*** ", (FAHCI Representative, Bouaké, November 2023).

The weaknesses at the organizational level are summarized in the problem related to communication and the involvement of certain programs as described by the Scientific Committee in these terms: " ***A lot of communication was needed from the different stakeholders, that is to say, people affected, people disabled by NTDs, programs that we are. It is true that there is the leprosy program, there is also the Buruli ulcer program which were not really involved. It is true that it is the program that carried the project - but it was also necessary to really involve all these programs because there is the Buruli ulcer program, and the CP NTD program also which have a lot of disabilities*** ", (Scientific Committee Representative, Abidjan, November 2023). For the Scientific Committee, it was necessary to involve all programs in the coordination and planning and above all communication should be in place within the management body of this project so that each stakeholder is at the same level of information.

- ***Project implementation arrangement***

Regarding the arrangements for implementing the dignity pilot project, the target played the greatest role in providing leadership. According to Coptiment , the FAHCI took ownership of the project to gain more support. This is reflected in the following statements: " ***The strengths are that, once people with disabilities have, through programming issues at the Ministry of Health, had the opportunity to take a leadership role in the conduct of the project. They took ownership of the project because they were the ones who appointed a project coordinator in Bouaké*** ," ( Coptiment Representative , Abidjan, November 2023).

The strategies and mechanisms for implementing the project constitute strengths that are important to mention for the FAHCI, which states this in these words: " ***the strategies and mechanisms in carrying out the project, we went through the media, the awareness communities through the radio, the regional radio which covers the Gbêkê region and several other regions, we went on the radio to make press releases, messages, we did two broadcasts per week, we communicated a lot*** ", (FAHCI Representative, Bouaké, November 2023).

The other strong point is that the FAHCI is in the process of finding strategies to implement this project on a national scale, as Coptiment points out : " ***the FAHCI having become aware of what we can do through the lessons they have received, - they are currently developing another phase of this project, to move to a large scale in Côte d'Ivoire. So that 's really the strong point, ensuring that it can continue and extend to the whole country*** ", ( Coptiment Representative , Abidjan, November 2023.

As for the weaknesses, the first is related to the time factor allocated to the implementation of the dignity pilot project, which is 12 months. This rather short implementation period means that the actors do not clearly perceive the effects of this project on the beneficiaries as well as the actions of the partners, as indicated in this statement: " ***there is time, I find that the time of the pilot phase is short and as I said, the means implemented are small so if it can have a sustainability phase, it must be*** ", (FAHCI Representative, Bouaké, November 2023).

The second weakness is the lack of a guaranteed fund to support PVMTN. Certainly, microfinance institutions are willing to support the project, but this handicap poses a challenge for the implementation of the dignity pilot project: " ***The COOPEC, for example, is willing to support people with disabilities, but they require approval. The FAHCI also lacks the necessary funds to guarantee, the necessary resources to ensure this; we need resources, a guaranteed fund to ensure this. It's true that we are making pleas, but there are institutions that will require approval, so we need a guaranteed fund. Create a monitoring committee to guarantee this*** ." (FAHCI Representative, Bouaké, November 2023).

Coptiment fits into this financial problem perspective. For him, the financial problem limited the actions of the project team and its partners: " ***the financial problem did not allow us to pay the people involved in the execution, but also to properly finance the activities that were planned for the project. - And then now the second thing, how to ensure that the important meetings that are scheduled can be held, the FAHCI at the Abidjan level to take part in them. There, that too we missed, the general implementation is not yet, validation of protocols, reports, major activities, everyday activities, they should be a little more involved but they were not able to ", (*** Coptiment Representative , Abidjan, November 2023).

- ***Project implementation***

As an initiative of the FAHCI, the pilot project dignity in its implementation consisted of first identifying people with disabilities due to illnesses or NTDs, gathering their needs and making pleas to the authorities in order to provide them with assistance. The project team made the effort to adhere to this perspective even if activities were not carried out. Thus, the strength that is perceived is the availability and participation of associations for the disabled and especially the PVMTN. In general, the FAHCI worked without great resources while respecting the orientations broken down into axes. This is what the PLMTN describes in these words: " ***We tried to decide on orientations or axes but what was important for me or I think it is really a strong point, is that an association, the FAHCI, itself carries out the activities*** ", (PLMTN Representative, Abidjan, November 2023).

Beyond this commitment, our survey revealed that several PVMTN were identified and registered, their needs were recorded, and educational and socio-professional integration centers were identified. In a word, the project brought the FAHCI closer to its members. The FAHCI describes the actions carried out by saying this: " ***The project allowed us to be closer to people with disabilities due to NTD diseases. And then we were also able to interact with them, understand the needs, the situations that these people experience. We were able to identify the needs of the people, which is the main objective, people with disabilities due to NTDs. We were able to contact institutions, advocate for people with disabilities due to neglected tropical diseases, their socioeconomic reintegration, and their rehabilitation. The institutions contacted: the youth employment agency, the legal clinic, social protection, town hall, the general directorate of health, I can name those, otherwise we contacted several. Microfinance: we have Coopec , Access Credit and Baobab*** ,” (FAHCI Representative, Bouaké, November 2023). Beyond the identification of the PVMTN, it must be said that they accepted and even took ownership of the project as can be seen in the words of the Bouaké women’s association: “ ***the strengths are that the project itself, the community accepted and adopted the project. That is a strength of the project itself and then with the health personnel and also they took ownership of the project*** ,” (Women’s Association Representative, Bouaké, November 2023).

The second strength is that the coordinator was truly committed with the support of all the programs and the involvement of Coptiment and the Scientific Committee. Coptiment testifies to this by emphasizing this: " ***At the local level, the coordinator who was committed also showed proactivity, really took the project in hand, although the project was limited, volunteers who worked, who ensured that the project was implemented ", (*** Coptiment Representative , Abidjan, November 2023).

However, there are some weaknesses that are worth mentioning. The first is related to the lack of material, financial, and health support at the PVMTN level. To this end, the Botro Health District is acting as the spokesperson for the PVMTN, drawing everyone's attention with these words: ***"I have the impression that we have finished registering the sick, we still have to take care of them. - very often they come to see me. And where are we with our project? No one called me. Because when Amoulaye came, he said he would call them. Now some have been called, some have not been called*** ," (Health District Representative, Botro, November 2023). Coptiment mentions other weaknesses which concern the very limited time factors, the unavailability of funds for the support and self-employment of PVMTN: " ***the time factor for the execution of such a strategy, it could have been a favorable element if we still had 1 year to see things through to the end to be able to even better guarantee sustainability - we were not able to provide mutual aid, when the principle is known. The other thing is the financing of sustainability or the funds to enable people to self-employ themselves",*** ( Coptiment Representative , Abidjan, November 2023).

Although these elements are weaknesses, they also constitute threats to the survival of the project. These observations show that the PVMTN expect to receive what was promised to them, which seems uncertain. In addition, the Committee reinforces this position by mentioning the weakness in communication. For him: " ***Weaknesses in the implementation of the project often require a lot of communication*** ," (Representative of the Scientific Committee, Abidjan, November 2023)

## IV.7 Analysis of the potential sustainability of project actions

What matters in this pilot project is the sustainability of the achievements. Currently, the radio awareness campaigns are going very well. But what can help preserve the achievements and, above all, ensure the potential sustainability of the project's actions is the support of PSH-MTN. From the surveys conducted, it appears that the PVMTN have not been supported until now. And their most ardent desire is to see the fulfillment of the promises made during their census. For the Scientific Committee, the time has come to support the PVMTN and, above all, to proceed with their socio-professional integration: " ***It's the economic aspect: supporting these people and their integration into our public services. It had a good impact because the FAHCI was really involved in this project. But I hope that the project can take socio-economic integration into account*** ," (Scientific Committee Representative, Abidjan, November 2023)

In addition to this socioeconomic component, all managers of the dignity pilot project must consider the possibility of extending this project so that its impacts are sufficiently visible. To this end, the PNEL would like the duration of the project to be extended so that activities continue to be carried out: " ***It's not about stopping it. It needs to be extended over a certain number of years, a certain period, and activities at the radio level continue. I think there was a good space there - to talk, and by dint of listening, it will change mentalities*** ," (PNEL Representative, Abidjan, November 2023). Thus, mentalities will change to lead communities to ban stigmatization, discrimination, and rejection.

In this perspective, the Regional Prefecture proposes that the dignity project be renewed and strengthened for the well-being of this vulnerable group that constitutes the PVMTN. In this regard, it puts forward the following: " ***That the project be renewed and strengthened through awareness-raising. That awareness-raising emphasizes the causes of NTDs, draws the attention of parents to the sanitation of the living environment. We must also take care of the PVMTN by providing them with kits for the creation of AGRs*** ", (Representative of the Gbêkê Regional Prefecture, Bouaké, November 2023).

Speaking of the project team and its arrangements, the PLMTN proposes reinforcement at several levels as stipulated in this statement: " **I think perhaps we will have to strengthen a little everything that is physical means, mobility, availability, financial, material, that is the aspect that needs to be improved a little more** ", (PLMTN Representative, Abidjan, November 2023).

## IV.8 Lessons learned and good practices

At the end of this project, all the implementing actors and partners recognize having learned lessons and good practices.

The first lesson is the successful experience of the participatory and integrated approach. This has generated the commitment and effective involvement of all partners in Bouaké and elsewhere: " ***the involvement of administrative authorities, the search for the commitment of the administrative authorities of a locality in which we are going to carry out a project*** " (PNEL Representative, Abidjan, November 2023).

The second lesson, the project allowed us to discover the existence of so-called eradicated diseases such as yaws in the Bouaké health region and the extent of the suffering of PVMTN: " ***as a lesson learned is that there are diseases that we thought no longer existed but we understood that there are diseases that still exist such as yaws. And this project allowed us to see these people who have this disease, Buruli ulcer, children who suffered from it*** " (Representative of the Bouaké North-West Health District, Bouaké, November 2023).

Other lessons learned include knowing how to live in a community, the importance of well-structured and organized work, the importance of contacts and, above all, good guidance, as summarized in the words of the FAHCI: " ***What I learned first was knowing how to live in a community, how to structure projects well, how to find my way. It gave me experience, contacts, and how to find my way. I saw serious cases that I had never seen before, and on a social and educational level, it taught me a lot*** ." (FAHCI Representative, Bouaké, November 2023). This experience constitutes a learning opportunity and above all the acquired capacity is social capital to be mobilized for the continuation of the project.

In terms of best practices, the project has boosted the activities of health districts by energizing the investigation process. It also involves the adoption of new strategies for early detection of NTDs and the development of contextual resilience strategies in the face of a number of challenges encountered in the community. " ***It boosts the focus on these investigation activities a little. It pushed them to supervise the CHWs; because the CHWs have been trained on recognizing these cases. When decisions are made, we call on the coordinator to be able to carry out supervisions now, if there are cases, we carry out investigations*** ," (Representative of the Bouaké North-West Health District, Bouaké, November 2023).

# **Conclusion and recommendations for the sustainability and survival of the project**

Since the baseline study carried out in 2021-2022 on the needs of PVMTN and PSH-MTN, the issue of NTDs has been sufficiently documented. These studies enabled the development of the dignity pilot project strategy and its implementation in the Gbêkê health region, an area endemic for several NTDs, of which leprosy and Buruli ulcer are the most common. To better understand the implementation of this pilot project, a mid-term evaluation took place in April 2023. This partial evaluation identified the strengths and weaknesses of the project's implementation while making recommendations likely to lead to the achievement of the objectives assigned to the dignity pilot project. A final evaluation was initiated to measure the effectiveness of the project in its planning and execution, to analyze the relevance, effectiveness, sustainability and effects of the project on the beneficiaries' experiences .

At the end of this final evaluation, we note that the project activities were implemented at 83.70%. This completion rate represents a very good level of satisfaction for all stakeholders. This result also makes us say that the short-term objectives of the project were achieved, although a proportion of 10.20% of the activities were not implemented at all. Today, FAHCI has a large database of PSH-MTN, PVMTN and their specific needs. It has carried out actions and made several pleas in favor of this vulnerable group by requesting various supports in collaboration with all the project stakeholders. These actions, which have generated significant gains, require provisions for their sustainability and especially for the survival of the project both in Gbêkê and its implementation at the national level. It must be recognized that the dignity pilot project was adopted by the beneficiaries and captured the commitment of the partners. Certainly, effective socioeconomic support, not being part of the objectives of the pilot project, was a point of disagreement between the implementing team and the beneficiaries. However, with the ongoing steps, the beneficiaries hope for a better tomorrow in terms of improving their situation at all levels. This also implies that the time factor did not allow for certain impacts, especially at the health and socioeconomic level, but overall the project deserves to be continued (Annex IX: List of processes underway with institutions collaborating with FAHCI). Let us also add that the immediate indicators have been achieved. These are awareness raising on knowledge of NTDs, which has improved the attitudes of several people (PVMTN, PSH-NTD, communities, etc.); capacity building of FAHCI associations and the implementing team; the realization of several advocacy campaigns towards various structures and administrations as well as requests for the improvement of the health of the project beneficiaries. The achievement of the other indicators is classified in the medium and long term since they will result from the steps currently in progress.

Taking into account all these findings, the stakeholders made the following proposals to ensure the effects of the project in the medium and long term:

***At the level of associations of people affected by NTDs:***

- - - 1. Continue advocacy with institutions as part of their activities to include people with mental health conditions (MSMTN) so that they can effectively mobilize resources dedicated to people affected by MTNs;
      2. Establish a technical monitoring and evaluation unit to strengthen coordination between the project team and FAHCI management;

1. Seek technical and financial partnerships in order to provide the FAHCI project team with substantial resources to continue project activities;
2. Advocate with institutions and programs to participate in strategy development, planning and awareness-raising activities to take into account the expectations of PSH-MTNs;
3. Maintain collaboration with health and psychological care structures and programs;

***At the level of health and psychological care structures and programs:***

1. Provide technical and financial support to FAHCI and other PSH associations;
2. Involve PSH associations in routine activities to combat NTDs;
3. Make effective free care and psychological support, in particular targeted free care for people affected by NTDs;

***At the level of the socio-economic and educational support structures and programs identified:***

1. Train people affected by NTDs in trades and employment strategies in the public and private sectors, including self-employment;
2. Mobilize funds to support people affected by NTDs in the development of Income Generating Activities;
3. Train PSH-MTNs on texts relating to their protection and promotion;
4. Promote access to basic education for people affected by NTDs;

***At the level of technical and financial partners (including the project funder)*** :

1. Provide technical and financial support to the dignity project over an additional three-year period to enable the achievement of medium-term objectives;
2. Supporting strategy scaling initiatives;
3. Provide technical and financial support for periodic monitoring and evaluations to better document successes, challenges and lessons learned.

# **Bibliography**

1.( Ndongo Paule Ylande et al., 2014, Community approaches in the fight against Buruli ulcer: literature review, in [Public Health​](https://www.cairn.info/revue-sante-publique.htm) [2014/HS (S1)](https://www.cairn.info/revue-sante-publique-2014-HS.htm) )

2. WHO, 2019, Neglected tropical diseases, EBI46/14

3. National Health Development Plan (NHDP) 2016-2020

4. Mid-term evaluation study protocol for the dignity project, 2023, Identification of a strategy to increase the influence of people affected by NTDs on access to care services for people affected by NTDs in Côte d'Ivoire

5. Final evaluation study protocol for the dignity project, 2023, Identification of a strategy to increase the influence of people affected by NTDs on access to care services for people affected by NTDs in Côte d'Ivoire

6. Mid-term evaluation report of the dignity project, 2023, Identification of a strategy to increase the influence of people affected by NTDs on access to care services for people affected by NTDs in Côte d'Ivoire

7. Study report, 2022, Identification of the specific needs of people with disabilities due to Neglected Tropical Diseases (PSH-NTDs) in Côte d'Ivoire

8. DIDR-OFPRA Report: People with disabilities - Ivory Coast 2018

9. Valérie Simonet (2014) Participatory local diagnosis on access to health care for people with disabilities in the district of Bankim , Cameroon, University of Lorraine,

# **Annexes**

## Appendix I: Information notice

**CONTEXT (What motivated this study)** : We are conducting this study with the aim of evaluating the initial effects of the Dignity pilot project on people with disabilities caused by NTDs in Côte d'Ivoire. Effect : hope and Coptiment have developed a partnership with Leprosy Research Initiative (LRI), one of the key players involved in the fight against NTDs worldwide. As part of this collaboration with LRI, it became necessary to conduct a final evaluation of the Dignity pilot project: "Identification of the specific needs of people with disabilities due to NTDs - Côte d'Ivoire".

**PURPOSE** : The purpose of this survey is to assess the effectiveness of the Dignity Pilot Project strategy to engage people with disabilities from neglected tropical diseases (NTDs) to influence government policies and programs to ensure a person-centered approach for people with disabilities in Côte d'Ivoire.

**OBJECTIVES** : The objectives assigned to this study are:

- Measure effectiveness in planning and executing project activities;
- Analyze the relevance, efficiency, effects of the project and their sustainability
- Analyze the effects before and after the implementation of the strategy developed by FAHCI and their sustainability in the Gbêkê region.
- Identify lessons learned and good practices from the project
- Analyze the project scaling assumptions.

**WHY WE ARE ASKING YOU TO PARTICIPATE**

You are invited to participate in this study. You may refuse to answer any questions you wish. If you agree to participate, the conversation will last less than 30 minutes. We will not collect your personal information, and your name will not be attached to the information or comments you provide. The data will be kept confidential with secure password access for the legal storage period and will be destroyed thereafter. No other use will be made of the data other than for the purposes of this study. We may also ask you to complete a very brief questionnaire at the beginning of the interview to determine whether you agree to participate in this study.

**STUDY PROCEDURES** : If you decide to participate in this study, we will administer an interview guide. We will ask you questions about your opinion on the project's conduct, the results achieved by the project, and its effect on the level of influence of people disabled by NTDs. With your permission, the information will be recorded on a dictaphone. The information collected will be confidential and only members of the study team will have access to it.

**BENEFIT** : As a direct benefit, a financial compensation of 1000F will be given to you at the end of the interview, also this study will be followed by the implementation of a pilot initiative to increase the capacity of influence and advocacy of people disabled by NTDs with a view to their psychosocial and economic care.

**TERMINATING CONSENT** : You are not required to agree to participate in this study. If you agree to participate, you may withdraw your consent at any time. Information obtained and used before you withdraw your consent will continue to be used for research. If you wish to withdraw your consent, please let us know.

**WHO CAN I CALL WITH QUESTIONS OR PROBLEMS?**

***Names and contact details of the investigators and that of the National Committee for Ethics in Life Sciences and Health:***

| - LOROU Bi Gohoré Jean-Maxime (Statistical Engineer) / [loroubi22@gmail.fr](mailto:loroubi22@gmail.fr) , 07 57 42 57 03 - TANOH Armand Hira, Principal Investigator, Director of CESI, [cabstat_info@yahoo.fr](mailto:cabstat_info@yahoo.fr) , 07 08 27 37 38 - Dr. Julien Aké, Physician, Community Health Specialist julien.ake@coptiment.com, 07 07 92 20 48 - Dr Louis PENALI: President of CNESVS (07 69 28 57 53) |
| --- |

## Appendix II: Written and informed consent form

**Final evaluation of the Dignity pilot project**

**“Identification of the specific needs of people with disabilities due to NTDs in Côte d’Ivoire”**

**WRITTEN AND INFORMED CONSENT OF THE STUDY PARTICIPANT**

***I have read the information note***

***I heard the information note read***

***I had the information note translated for me.***

***I have received and understood the explanations given on this research***

The nature of this research was explained to me. I understood the nature of this research and the information text. I asked all my questions and received satisfactory answers.

I freely agree to participate in an individual interview and/or possibly a group interview, which will be recorded. I understand that I can terminate my participation at any time during the discussions, without prejudice or explanation.

Do you agree to participate in the study? Yes No

**Participant**

Signature of adult participant: __________________________________________________

Date / / Place ________________________

**Investigator**

Name of person obtaining consent: ______________________________________

Date / / Place Signature__________________

## Appendix III: Confidentiality Agreement

**Evaluation of the Dignity pilot project: “Identification of the specific needs of people with disabilities due to NTDs in Côte d’Ivoire”**

I, the undersigned………………………………………………………………………………………………………………………………

Status in the team: ………………………………………………………………………………………………… ……. .

Contact : ……………………………………………………………………………………………………………………… ……. .,

I undertake , under the Survey Practice Act, to protect the confidentiality of information provided by respondents. To this end, personal data that allows the respondent to be identified will not be published.

Furthermore, I undertake to scrupulously respect the rules of ethics and professional conduct.

In the event of non-compliance with these commitments, the Statistical and Computer Studies Firm (CESI) is free to undertake procedures that it deems appropriate.

Date / ___/___/________/ **PROJECT MANAGER**

(Name and signature)

Agent's signature:

## Appendix IV: Data collection tools

Focus group guide

- 1 focus with people directly affected by leprosy

- 1 focus with people directly affected by Buruli Ulcer

- 1 focus with the heads of NTD associations

**I Knowledge of the dignity project**

- What do you mean by MTN and the dignity project?
- What do you know about the dignity project?
- How were you informed?
- What are the strategies for implementing this project?
- What are the socio-economic needs of the MTN PSH targeted by this project?
- What did this project do for you?
- Did this project help you to gain more knowledge on the following topics ( *measure the level of knowledge of NTD PSH on these topics)* :
  - Symptoms of NTDs according to the types present
  - Possible causes and modes of transmission of NTDs according to the types present
  - structures for the care of PSH MTN (rehabilitation centers, re-education centers, specialized care centers, training centers, other centers)
  - laws and regulations that protect people with disabilities
- What was your situation before the project was carried out?
- What is your situation today?

**II Implementation of the dignity project and its impacts**

- What do you think about the implementation of the dignity project?
- What are your thoughts on the planning and execution of this project?
- What are the strengths and weaknesses of the implementation of this project?
- What changes did this project bring you?
- How satisfied are you with the implementation of the pilot project?
- What do you think about the effectiveness of the dignity project?
- What are the obstacles to implementing this project?
- Assessment of the attitudes of PSH MTN
  - Forms of stigma and discrimination and their extent;
  - the extent of self-stigma.
- Assessment of the effects of the project
  - Free access to health care and medicines,
  - Knowledge and practice of good practices as a result of the project (to take care of oneself)
  - Free access to rehabilitation and re-education services as a result of the project;
  - Free access to psychological support as a result of the project;
  - Access to awareness raising on NTDs
  - Free access to training and/or facilitation of schooling as a result of the project
  - Facilitating the membership of MTN PSH in an association as a result of the project
  - Access to economic strengthening activities (periodic monetary transfers, initiation of Income Generating Activities, self-employment)
  - Organization of NTD detection campaigns as a result of the project
  - Strengthening PSH-MTN Organizations by giving them the capacity to advocate for the promotion of their rights
  - Empowerment of PSH-MTN Organizations for the development of strategic partnerships at different levels *(cite the partnerships developed)*

**III Lessons learned and proposals for preserving achievements**

- What did you learn from this project?
- What are the best practices received by this project?
- What do you propose to preserve acquired skills?
- What are your proposals for the survival of this project?
- How can we proceed to extend this project to all regions in Ivory Coast?

**Appendix V: Interview guide for focal points**

- National Leprosy Elimination Program (PNEL)
- National Program to Combat Buruli Ulcer (PNLUB)
- National Program for the Fight against Neglected Tropical Diseases with Preventive Chemotherapy (PNLMTN-CP)
- FAHCI leaders
- Coptiment Project Team
- Other Dignity Project Implementers

**I Knowledge of the dignity project**

- What do you know about the dignity project?
- What is your level of involvement?
- What are the socio-economic needs of the MTN PSH targeted by this project?
- What did this project allow you to achieve for PSH-MTN?
- What was the PSH-MTN situation before the project was implemented?
- What is their situation today?

**II Implementation of the dignity project and its impacts**

- How did you participate in the execution of this project?
- What are the strategies and mechanisms for its implementation?
- What actions have you taken towards the target of this project?
- What do you think of your actions towards PVMTN?
- Which project activities have achieved their objectives?
- Which project activities did not achieve their objectives?
- What do you think about the implementation of the dignity project?
- What are your thoughts on the planning and execution of this project?
- What are the strengths and weaknesses in the implementation of this project?
- What changes has this project brought to PSH-MTNs?
- How satisfied are you with the implementation of the pilot project?
- What do you think about the effectiveness of the dignity project?
- What are the obstacles to implementing this project?
- What are the different impacts of this project on PSH-MTN?
  - Free access to health care and medicines,
  - Free access to rehabilitation and re-education services as a result of the project;
  - Free access to psychological support as a result of the project;
  - Access to awareness raising on NTDs, improvement of knowledge and practice of good practices as a result of the project (to take care of oneself)
  - Free access to training and/or facilitation of schooling as a result of the project
  - Facilitating the membership of MTN PSH in an association as a result of the project
  - Access to economic strengthening activities (periodic monetary transfers, initiation of Income Generating Activities, self-employment)
  - Organization of NTD detection campaigns as a result of the project
  - Strengthening PSH-MTN Organizations by giving them the capacity to advocate for the promotion of their rights
  - Empowerment of PSH-MTN Organizations for the development of strategic partnerships at different levels *(cite the partnerships developed)*
- What are the expectations of MTN PSH that the project has not taken into account?

**III Lessons learned and proposals for preserving achievements**

- What did you learn from this project?
- What are the best practices received by this project?
- What do you propose to preserve acquired skills?
- What are your proposals for the survival of this project?
- How can we extend this project to all regions in Ivory Coast?
- Are there any assumptions or preconditions that need to be verified before this extension?

**Appendix VI: Interview guide for health practitioners**

- Gbêkê Health Region
- Health workers from the Bouaké Health Districts involved in the management of NTDs
- ASC of the Health District involved in the management of NTDs
- Other actors

**I Knowledge of the dignity project**

- What do you know about the dignity project?
- What is your level of involvement?
- What are the socio-economic needs of the MTN PSH targeted by this project?
- What did this project allow you to achieve for PSH-MTN?
- What was the PSH-MTN situation before the project was implemented?
- What is their situation today?

**II Implementation of the dignity project and its impacts**

- How did you execute this project?
- What are the strategies and mechanisms for its implementation?
- What actions have you taken towards the target of this project?
- What do you think of your actions towards PVMTN?
- Which project activities have achieved their objectives?
- Which project activities did not achieve their objectives?
- What do you think about the implementation of the dignity project?
- What are your thoughts on the planning and execution of this project?
- What are the strengths and weaknesses in the implementation of this project?
- What changes has this project brought to PSH-MTNs?
- How satisfied are you with the implementation of the pilot project?
- What do you think about the effectiveness of the dignity project?
- What are the obstacles to implementing this project?
- What are the different impacts of this project on PSH-MTN and you?
  - Free access to health care and medicines,
  - Free access to rehabilitation and re-education services as a result of the project;
  - Free access to psychological support as a result of the project;
  - Access to awareness raising on NTDs, improvement of knowledge and practice of good practices as a result of the project (to take care of oneself)
  - Free access to training and/or facilitation of schooling as a result of the project
  - Facilitating the membership of MTN PSH in an association as a result of the project
  - Access to economic strengthening activities (periodic monetary transfers, initiation of Income Generating Activities, self-employment)
  - Organization of NTD detection campaigns as a result of the project
  - Strengthening PSH-MTN Organizations by giving them the capacity to advocate for the promotion of their rights
  - Empowerment of PSH-MTN Organizations for the development of strategic partnerships at different levels *(cite the partnerships developed)*
- What are the expectations of MTN PSH that the project has not taken into account?

**III Lessons learned and proposals for preserving achievements**

- What did you learn from this project?
- What are the best practices received by this project?
- What do you propose to preserve acquired skills?
- What are your proposals for the survival of this project?
- How to proceed with the implementation of this project in Ivory Coast?

**Appendix VII: Interview guide for institutions**

- Gbêkê regional prefecture
- Labor inspection,
- Youth employment,
- Social Protection Department
- Caritas Project Team
- Community leaders

**I Knowledge of the dignity project**

- What do you know about the dignity project?
- How did you find out about this project?
- What are the socio-economic needs of the MTN PSH targeted by this project?
- What did this project allow you to achieve for PSH-MTN?
- What was the PSH-MTN situation before the project was implemented?
- What is their situation today?
- What did you learn from this project?

**II Implementation of the dignity project and its impacts**

- What is your level of involvement in the project?
- What do you think about the planning and execution of the activities of this project?
- What actions have you taken towards the target of this project?
- What do you think of your actions towards PVMTN?
- Which project activities have achieved their objectives?
- Which project activities did not achieve their objectives?
- What do you think about the implementation of the dignity project?
- What are your thoughts on the planning and execution of this project?
- What are the strengths and weaknesses in the implementation of this project?
- What changes has this project brought to PSH-MTNs?
- How satisfied are you with the implementation of the pilot project?
- What do you think about the effectiveness of the dignity project?
- What are the obstacles to implementing this project?
- What are the different impacts of this project on PSH-MTN and you?
- What are the expectations of MTN PSH that the project has not taken into account?

**III Lessons learned and proposals for preserving achievements**

- What did you learn from this project?
- What are the best practices received by this project?
- What do you propose to preserve acquired skills?
- What are your proposals for the survival of this project?
- How to proceed with the implementation of this project in Ivory Coast?

**Appendix VIII: Interview guide for communications officers**

- Bouaké Radio
- Press
- Other actors

**I Knowledge of the dignity project**

- What do you know about the dignity project?
- How did you participate in the execution of this project?
- What is your level of involvement in the implementation of this project?
- What was the PSH-MTN situation before the project was implemented?
- What is their situation today?
- What did you learn from this project?

**II Implementation of the dignity project and its impacts**

- What are your strategies and mechanisms for its implementation?
- How often do you intervene?
- What actions have you taken towards the target of this project?
- What themes did you cover?
- What do you think of your actions towards PVMTN?
- Which project activities have achieved their objectives?
- Which project activities did not achieve their objectives?
- What did this project allow you to achieve for PSH-MTN?
- What do you think about the implementation of the dignity project?
- What are your thoughts on the planning and execution of this project?
- What are the strengths and weaknesses in the implementation of this project?
- What changes has this project brought to PSH-MTNs?
- How satisfied are you with the implementation of the pilot project?
- What do you think about the effectiveness of the dignity project?
- What are the obstacles to implementing this project?
- What are the different impacts of this project on PSH-MTN and you?
- What are the expectations of MTN PSH that the project has not taken into account?

**III Lessons learned and proposals for preserving achievements**

- What did you learn from this project?
- What are the best practices received by this project?
- What do you propose to preserve acquired knowledge?
- What are your proposals for the survival of this project?
- How to proceed with the implementation of this project in Ivory Coast?

**Annex IX: PROCESSES IN PROGRESS WITH INSTITUTIONS COLLABORATING WITH THE FAHCI**

| **No.** | **INSTITUTIONS** | **PLEAS MADE** | **CURRENT ACTIONS** |
| --- | --- | --- | --- |
| **01** | **Bouaké Youth Employment Agency** | - Support for PSH-MTN in their activities - Funding projects for PSH-MTNs | - Canvas of action plans of the youth employment agency made available to PSH-MTN for the formulation of their projects. - Action plan to be submitted to the Youth Employment Agency to benefit from support and financing for projects planned by the management of the youth employment agency |
| **02** | **Social Protection Department** | - Free CMU contributions for PSH-MTN | - List of identified PSH-MTNs made available to the said department then sent to the Ministry of Solidarity and the Fight against Poverty. - Follow-up to be done with the Regional Directorate of MEPS |
| **03** | **CARITAS Bouaké** | - Advocacy for the care and free treatment of PSH-MTN | - List of PSH-MTN filed with Father Achille |
| **04** | **Social Service of the Town Hall of the commune of Bouaké** | - Involving PSH-MTN in the community's social actions | - The status and internal regulations of the FAHCI requested by the director of the social service so that the project benefits from the social actions of the town hall |
| **05** | **Department of National Education and Literacy**  **(DRENA)** | - School support for students with disabilities due to neglected tropical diseases - Free school kits for students with disabilities due to neglected tropical diseases | - Plea accepted by management - List of students with disabilities due to neglected tropical diseases with basic education needs filed. |
| **06** | **Ivorian Radio and Television Broadcasting (RTI-Bouaké)** | - Help disseminate and convey awareness messages about neglected tropical diseases. - Media coverage of the project ceremonies | - Availability of management to facilitate and ensure the dissemination of awareness messages and media coverage of project activities |
| **07** | **COOPEC Bouaké** | - Supporting PSH-MTNs in their activities - Funding projects for PSH-MTNs | - Seeking approval to guarantee the funds that will be made available to PSH-MTN - Support and financing of projects planned by the agency head |
| **08** | **Regional Directorate of Labor Inspection** | - Request for texts protecting PSH-MTN - Provide the project with a specialist to raise awareness of the rights and duties of PSH-MTN in the world of work | - Test made available to the project team. - A specialist will be made available to the project |
| **09** | **Vatelot Health Center in Bouaké** | - Support for PSH-MTN (care and physical rehabilitation) | - List of PSH-MTN still suffering from Buruli ulcer submitted to the director of the center. |
| **10** | **Legal Clinic Management** | - Promote the improvement and access to rights of PSH-MTN - Promoting equitable access to justice for PSH-MTN - Collaboration with the Dignity Project team | - Plea accepted by management - List of people with disabilities due to neglected tropical diseases filed with the director. - Connecting the project team with other institutions whose mission is to protect PSH-MTNs |
| **11** | **Bouaké Court of First Instance** | - Help protect PSH-MTN - Enforcing the laws of people with disabilities | - Guarantee the protection of every citizen - Availability for any matter aimed at protecting PSH-MTN |
