# Supplementary material for: People-centered strategies to mobilize people living with disabilities due to Neglected Tropical Diseases (PD-NTDs) to influence policy and programs: A mixed-methods study in Côte d’Ivoire
Source: PLoS Negl Trop Dis. 2025 Sep 8;19(9):e0013485. doi: 10.1371/journal.pntd.0013485 (PMC12431663; doi:10.1371/journal.pntd.0013485)
Supplement: S1 File — (ZIP) [file pntd.0013485.s007.zip › Guides for Reps of National Programs.docx]

Interview guide for representatives of national programs (Leprosy Control (PNEL); Buruli Ulcer Control (PNLUB); NTD Control (PNLMTN CP))

I- Introduction of the interviewee and the organization

1- Full name

2- Position and title

3- Number of years in the program

4- Brief introduction of the organization

II- Missions and activities of the organization related to NTDs

1- What are the existing laws, policies, systems, and mechanisms aimed at supporting people with disabilities, including those affected by NTDs, at both the national and regional levels? (Provide documentation if available)

……………………………………………………………………………………………………………………………………………………………………

…………………………………………………………………………………………………………………………………………………………..

2- Describe the support system in place for people disabled by NTDs in Côte d'Ivoire.

……………………………………………………………………………………………………………………………………………………………………………

…………………………………………………………………………………………………………………………………………………………….

3- What are the existing systems, mechanisms, and programs for psychosocial and economic support for people disabled by NTDs?

……………………………………………………………………………………………………………………………………………………………

…………………………………………………………………………………………………………………………………………………………..

4- What provisions are in place for morbidity management and disability prevention?

……………………………………………………………………………………………………………………………………………………………

…………………………………………………………………………………………………………………………………………………………..

5- Are there case management services? If so, what are they? ………………………………………………………………………………………………………………………………………………………………………

…………………………………………………………………………………………………………………………………………………………………

6- What do you think are the specific and priority needs of people with disabilities due to NTDs in Côte d'Ivoire at the social and economic levels (education, health, and access to the labor market)?

a- Health

……………………………………………………………………………………………………………………………………………………………

…………………………………………………………………………………………………………………………………………………………

b- Education

……………………………………………………………………………………………………………………………………………………………

……………………………………………………………………………………………………………………………………………………………

c- Access to the labor market

……………………………………………………………………………………………………………………………………………………………

……………………………………………………………………………………………………………………………………………………………

7- What do you think are the main obstacles faced by people with disabilities in education, health, and access to the labor market?

a- Health Health

………………………………………………………………………………………………………………………………………………………………………………………………………………………………………………………………………………………………………………………………………………………………………………………………………………………………………………………………………………………………..

b- Education

……………………………………………………………………………………………………………………………………………………………………………………………………………………………………………………………………………………………………………………………………………………………………………………………………………………………………………………………………………………………………………………..

c- Access to the labor market

………………………………………………………………………………………………………………………………………………………………………………………………………………………………………………………………………………………………………………………………………………………………………………………………………………………………………………………………………………………………………………………………………………………

8- What do you think are the sources of stigmatization and exclusion in Cote d’Ivoire’s policies and regulations? ………………………………………………………………………………………………………………………………………………………………………………………………………………………………………………………………………………………………………………………………………………………………………………………………………………………………………………………………………………………

III- Suggestions and proposed solutions related to the situation of people with disabilities due to NTDs

…………………………………………………………………………………………………………………………………………………………………………………………………………………………………………

………………………………………………………………………………………………………………………………………………………………………………………………………………………………………………………………………………………………………………………………………

THANK YOU FOR YOUR AVAILABILITY
