# Supplementary material for: People-centered strategies to mobilize people living with disabilities due to Neglected Tropical Diseases (PD-NTDs) to influence policy and programs: A mixed-methods study in Côte d’Ivoire
Source: PLoS Negl Trop Dis. 2025 Sep 8;19(9):e0013485. doi: 10.1371/journal.pntd.0013485 (PMC12431663; doi:10.1371/journal.pntd.0013485)
Supplement: S1 File — (ZIP) [file pntd.0013485.s007.zip › InterviewGuide_MinistryofJustice.docx]

Interview Guide for Representatives of the Ministries

I- Introduction of the Interviewee and the Organization

1- Full Name

2- Position and Title

3- Number of Years at the Ministry

4- Brief Introduction of the Department

II- Missions and Activities of the Organization Related to NTDs

**1- What are the existing laws, policies, systems, mechanisms, and programs aimed at supporting people with disabilities, including those affected by NTDs, at both the national and regional levels? (Provide documentation if available)**

……………………………………………………………………………………………………………………………………………………………

**2- What policies and regulations have been developed by your Ministry to support the situation of people with disabilities in the country? (Provide documentation if available)**

……………………………………………………………………………………………………………………………………………………………

**3- What are the existing mechanisms for psychosocial and economic support for people with disabilities affected by NTDs within your Ministry?** ………………………………………………………………………………………………………………………………………………………………………

**4- What do you consider to be the main obstacles encountered by people with disabilities?**

**a- Health**

……………………………………………………………………………………………………………………………………………………………………………

**b- Education**

……………………………………………………………………………………………………………………………………………………………

**c- Access to the labor market**

……………………………………………………………………………………………………………………………………………………………

**5- Describe the support system implemented by your ministry for PD-NTDs in Côte d'Ivoire**

……………………………………………………………………………………………………………………………………………………………………………

**6- What are the existing systems, mechanisms, and programs for psychosocial and economic support for PD-NTDs in your ministry?**

……………………………………………………………………………………………………………………………………………………………

**7- What do you consider to be the specific and priority social and economic needs of PD-NTDs in Côte d'Ivoire?**

**a- Health**

………………………………………………………………………………………………………………………………………………………………………………

**b- Education**

……………………………………………………………………………………………………………………………………………………………………………

**c- Access to the labor market**

……………………………………………………………………………………………………………………………………………………………

**8- In your opinion, what are the main obstacles encountered by people with disabilities?**

**a- Health**

……………………………………………………………………………………………………………………………………………………………

**b- Education**

……………………………………………………………………………………………………………………………………………………………

**c- Access to the labor market**

……………………………………………………………………………………………………………………………………………………………

**9- In your opinion, what are the sources of stigma and exclusion in policies and regulations in Côte d'Ivoire?**

…………………………………………………………………………………………………………………………………………………………….

**10- What provisions are in place for the integration of people with disabilities through NTDs in Côte d'Ivoire in terms of access?**

**a- Health**

………………………………………………………………………………………………………………………………………………………………………………

**b- Education**

……………………………………………………………………………………………………………………………………………………………………………

**c- Labor Market**

……………………………………………………………………………………………………………………………………………………………………………

**11- Are there laws to punish stigmatization and exclusion of people with disabilities in general and by those with disabilities due to NTDs in Côte d'Ivoire?**

………………………………………………………………………………………………………………………………………………………………………..

**III- Suggestions and proposed solutions related to the situation of people with disabilities due to NTDs**

…………………………………………………………………………………………………………………………………………………………………………………………………………………………
