# Supplementary material for: People-centered strategies to mobilize people living with disabilities due to Neglected Tropical Diseases (PD-NTDs) to influence policy and programs: A mixed-methods study in Côte d’Ivoire
Source: PLoS Negl Trop Dis. 2025 Sep 8;19(9):e0013485. doi: 10.1371/journal.pntd.0013485 (PMC12431663; doi:10.1371/journal.pntd.0013485)
Supplement: S1 File — (ZIP) [file pntd.0013485.s007.zip › FocusGroups_NTDs.docx]

FOCUS GROUP DISCUSSION GUIDE WITH PD-NTDs

I- Introduction of participants

- List of participants:

o Full name

o Age

o Level of education/study

o Number of years with disability

o Type of disability

II- Knowledge, attitudes, and practices of people affected by leprosy, Buruli ulcer, yaws, lymphedema, and hydrocele associated with lymphatic filariasis, regarding NTDs, existing policies, regulations, and support services.

Knowledge (discover the level of knowledge about the phenomenon and assess stakeholders' perceptions of both NTDs and support services)

o What existing laws and regulations regarding the protection of people with disabilities are you aware of?

o What care and support policies and mechanisms are you aware of?

o Are you familiar with existing healthcare, rehabilitation, and reeducation services? List them.

o Are you familiar with the organizations for people with disabilities operating in the country and in the pilot area?

o What are your relationships with these organizations?

o What do you think are the causes/sources/origins of NTDs?

o What are the consequences of the NTDs that you are aware of?

o Describe the symptoms of NTDs.

o Describe the modes of transmission of NTDs.

o What are the appropriate treatments for NTDs?

o What are the specific needs of people with disabilities?

Attitudes

o What judgments (favorable or unfavorable) do you make or have regarding people with disabilities? ………………………………………………..

What is your state of mind regarding this situation? (Defeatist or optimistic attitude)……

o Stigma / Discrimination

Self-stigma/Perceived vulnerability:

- Do you think you are vulnerable? Justify your answer……………………

Experienced stigma:

- Can you describe situations of stigma you have experienced?.....................................................

Perceived stigma:

- Can you describe situations of perceived stigma?.....................................................

Social participation (perceived advantages and obstacles related to your disability):

- Do you participate in community or association activities? If yes, what advantages do you derive from them; if no, what are the obstacles to your non-participation?...........

Self-confidence and willingness to change:

- Do you have confidence in yourself and your abilities? Justify your answer…………

Cultural Factors that Affect Perceptions of NTDs

Beliefs about NTDs and disability

- Do you think the causes or origins of NTDs are natural or mystical? Justify your answer………………………………………………….

Practices regarding NTDs

o Behaviors

Social behavior (social and professional integration)

- Do you have an income-generating activity? …………………………………….
- Do you belong to an association?…………………………………………………………
- Are you an activist or participate in community activities?.....................................

Medical behavior (taking into account physical and mental health, treatment adherence, etc.)

- Are you under the care of a doctor? ………………………………………………….
- Do you take your medication regularly? ……………………………………

Access to care / treatment regimen

- What is your treatment regimen?…………………………………………………………
- What are the different types of treatment you provide?................................................

…………………………………………………………………………………………………

III- Suggestions and proposed solutions related to the situation of people with disabilities due to NTDs

THANK YOU FOR YOUR COLLABORATION
