# Supplementary material for: People-centered strategies to mobilize people living with disabilities due to Neglected Tropical Diseases (PD-NTDs) to influence policy and programs: A mixed-methods study in Côte d’Ivoire
Source: PLoS Negl Trop Dis. 2025 Sep 8;19(9):e0013485. doi: 10.1371/journal.pntd.0013485 (PMC12431663; doi:10.1371/journal.pntd.0013485)
Supplement: S1 File — (ZIP) [file pntd.0013485.s007.zip › FAHCI.docx]

Interview Guide for FAHCI Representatives

I- Introduction of the Interviewee and the Organization

1- Full Name

2- Position and Title

3- Number of Years with the Organization

4- Brief Introduction of the Organization

II- Missions and Activities of the Organization Related to NTDs

1- What are the existing laws, policies, systems, mechanisms, and programs aimed at supporting people with disabilities, including those affected by NTDs, at both the national and regional levels? (Provide documentation if available)

……………………………………………………………………………………………………………………………………………………………

2- What policies and regulations have been developed to support the situation of people with disabilities in the country? (Provide documentation if available)

……………………………………………………………………………………………………………………………………………………………

3- What are the existing mechanisms for psychosocial and economic support for people with disabilities affected by NTDs? ………………………………………………………………………………………………………………………………………………………………………

4- What do you think are the main obstacles faced by people with disabilities?

a- Access to education

……………………………………………………………………………………………………………………………………………………………

b- Health care

……………………………………………………………………………………………………………………………………………………………

c- Access to the labor market

……………………………………………………………………………………………………………………………………………………………

5- Describe the support system in place for people with disabilities due to NTDs in Côte d'Ivoire

……………………………………………………………………………………………………………………………………………………………

6- What are the existing systems, mechanisms, and programs for psychosocial and economic support for people with disabilities due to NTDs in Côte d'Ivoire?

……………………………………………………………………………………………………………………………………………………………

7- What do you think are the specific and priority social and economic needs of people with disabilities due to NTDs in Côte d'Ivoire? a- Health

………………………………………………………………………………………………………………………………………………………………………………

b- Education

……………………………………………………………………………………………………………………………………………………………………………

c- Access to the labor market

……………………………………………………………………………………………………………………………………………………………………………

8- In your opinion, what are the main obstacles encountered by people with disabilities?

a- Health

……………………………………………………………………………………………………………………………………………………………………………

b- Education

……………………………………………………………………………………………………………………………………………………………

c- Access to the labor market

……………………………………………………………………………………………………………………………………………………………

9- In your opinion, what are the sources of stigma and exclusion in policies and regulations in the field of disability?

…………………………………………………………………………………………………………………………………………………………….

10- What provisions are in place for the integration of people with disabilities through NTDs in Côte d'Ivoire in terms of access?

a- Health

………………………………………………………………………………………………………………………………………………………………………………

b- Education

……………………………………………………………………………………………………………………………………………………………………………

c- Labor Market

……………………………………………………………………………………………………………………………………………………………………………

11- What provisions are in place for morbidity management and disability prevention?

…………………………………………………………………………………………………………………………………………………………….

12- Are there any case management services that you are aware of?

………………………………………………………………………………………………………………………………………………………..

13- Are there laws to punish the stigmatization and exclusion of people with disabilities caused by NTDs in Côte d'Ivoire? ………………………………………………………………………………………………………………………………………………………..

14- Are there formal collaborative relationships between the FAHCI and the Ministries of Health, Education, Employment, Protection of Persons with Disabilities, and Justice?

………………………………………………………………………………………………………………………………………………………………………..

III- Suggestions and proposed solutions related to the situation of PD-NTDs

…………………………………………………………………………………………………………………………………………………………………………………………………………………………
