# Supplementary material for: People-centered strategies to mobilize people living with disabilities due to Neglected Tropical Diseases (PD-NTDs) to influence policy and programs: A mixed-methods study in Côte d’Ivoire
Source: PLoS Negl Trop Dis. 2025 Sep 8;19(9):e0013485. doi: 10.1371/journal.pntd.0013485 (PMC12431663; doi:10.1371/journal.pntd.0013485)
Supplement: S1 File — (ZIP) [file pntd.0013485.s007.zip › Questions pour exploration des connaissances des malades et accompagnantsQR2.docx]

**Questions to explore the knowledge of patients and their caregivers**

| Questions | Maladies | | | | |  |
| --- | --- | --- | --- | --- | --- | --- |
|  | Leprosy | Buruli Ulcer | Yaws | Lymphatic filariasis | | Onchocerciasis |
|  |  |  |  | Elephantiasis  (Large limbs) | Hydrocele (Large testicle) |  |
| What causes this disease ? | - A microbe | - A microbe | - A microbe | - A parasite | - A parasite | - A parasite |
| What are the modes of transmission ? | - Prolonged close contact with an untreated person - Nasal secretions - Saliva - Contaminated objects | - Working without protection in swamps - Swimming in backwaters - Doing household chores in backwaters (laundry, dishes, etc.) - Being bitten by insects in the water | - Contact with an untreated person - Contaminated objects | - Mosquito Bite | - Mosquito Bite | - Small black fly bite |
| What are the possible signs/symptoms of the disease ? | - Lighter patch on skin with little or no sensation - Nodule (pimple) on the skin - Loss of sensation in the fingers and feet - Tingling in the hands and feet - Poor eye closure - Painless sore | - Nodule (button) with little tenderness - Hard area of ​​skin (plaque) with little tenderness - Significant swelling of the limbs or face - Large, slightly painful sores | - Wounds - Yellowish bumps - Pain in bones and joints - Deformity of the bones in the fingers and toes | - Progressive painless swelling of a limb (arm, feet) - Progressive painless swelling of a breast - Painful attacks in case of infection of the swollen limb | - Painless progressive swelling of testicles | - Itchy skin - Soft lump under the skin, especially around the waist - "Lizard-like" skin - Decreased vision - Loss of vision |
| Can we cure the disease? | - Yes | - Yes | - Yes | - No; difficult to treat when complications arise | - Yes | - Yes |
| What is the treatment? | - Oral antibiotics for 6 months or 12 months depending on the form - Treatment of lesions | - Oral antibiotics for 8 weeks - Treatment of lesions | - A dose of antibiotic; oral or injection - Treatment of lesions | - Mass drug treatment - Lifelong washing and hygiene of the affected limb - Complex and very expensive surgery. | - Mass drug treatment - Washing and hygiene of swollen testicles - Surgery (operation) | - Prevention by mass drug treatment |
| What can be the consequences if one is not treated quickly or not treated at all? | - Deformity  - Loss of organs  - Loss of limbs  - Loss of sight  - Loss of mobility | - Deformity  - loss of organs  - Loss of limbs  - Loss of mobility | - Pain in bones and joints - Permanent deformity of the bones of the fingers and feet | - Pain crisis in case of infection of the swollen limb - Systemic infection - Loss of mobility - Death from systemic infection | - Pain attacks in case of infection - Limitation of movement due to the increasing weight of the testicles. | - Permanent vision loss  - "Lizard-like" skin |
