# Supplementary material for: People-centered strategies to mobilize people living with disabilities due to Neglected Tropical Diseases (PD-NTDs) to influence policy and programs: A mixed-methods study in Côte d’Ivoire
Source: PLoS Negl Trop Dis. 2025 Sep 8;19(9):e0013485. doi: 10.1371/journal.pntd.0013485 (PMC12431663; doi:10.1371/journal.pntd.0013485)
Supplement: S1 File — (ZIP) [file pntd.0013485.s007.zip › MinistryofEducation .docx]

Interview Guide for Representatives of the Ministry of National Education

I- Introduction of the Interviewee and the Organization

1- Full Name

2- Position and Title

3- Number of Years at the Ministry

4- Brief Introduction of the Department

II- Missions and Activities of the Organization Related to NTDs

1- What laws, policies, systems, mechanisms, and programs exist to support people with disabilities, including those affected by NTDs, at both the national and regional levels? (Provide documentation if available)

……………………………………………………………………………………………………………………………………………………………

2- What policies and regulations have been developed by your Ministry to address the situation of PD-NTDs in the country? (Provide documentation if available)

……………………………………………………………………………………………………………………………………………………………

3- What mechanisms exist for psychosocial and economic support for PD-NTDs within your Ministry? ………………………………………………………………………………………………………………………………………………………………………

4- What do you consider to be the main obstacles encountered by people with disabilities?

a- Health

……………………………………………………………………………………………………………………………………………………………………………

b- Education

……………………………………………………………………………………………………………………………………………………………

c- Access to the labor market

……………………………………………………………………………………………………………………………………………………………

5- Describe the support system implemented by your ministry for PD-NTDs in Côte d'Ivoire

……………………………………………………………………………………………………………………………………………………………………………

6- What are the existing systems, mechanisms, and programs for psychosocial and economic support for PD-NTDs in your ministry?

……………………………………………………………………………………………………………………………………………………………

7- What do you consider to be the specific and priority social and economic needs of PD-NTDs in Côte d'Ivoire?

a- Health

………………………………………………………………………………………………………………………………………………………………………………

b- Education

……………………………………………………………………………………………………………………………………………………………………………

c- Access to the labor market

……………………………………………………………………………………………………………………………………………………………

8- In your opinion, what are the main obstacles encountered by people with disabilities?

a- Health

……………………………………………………………………………………………………………………………………………………………

b- Education

……………………………………………………………………………………………………………………………………………………………

c- Access to the labor market

……………………………………………………………………………………………………………………………………………………………

9- In your opinion, what are the sources of stigma and exclusion in policies and regulations in the Ivory Coast?

…………………………………………………………………………………………………………………………………………………………….

10- What provisions are in place for the integration of PD-NTDs in Côte d'Ivoire in terms of access?

a- Health

………………………………………………………………………………………………………………………………………………………………………………

b- Education

……………………………………………………………………………………………………………………………………………………………

c- Labor Market

……………………………………………………………………………………………………………………………………………………………

III- Suggestions and Proposed Solutions Related to the Situation of PD-NTDs

…………………………………………………………………………………………………………………………………………………………………………………………………………………………

THANK YOU FOR YOUR AVAILABILITY
