# Supplementary material for: People-centered strategies to mobilize people living with disabilities due to Neglected Tropical Diseases (PD-NTDs) to influence policy and programs: A mixed-methods study in Côte d’Ivoire
Source: PLoS Negl Trop Dis. 2025 Sep 8;19(9):e0013485. doi: 10.1371/journal.pntd.0013485 (PMC12431663; doi:10.1371/journal.pntd.0013485)
Supplement: S1 File — (ZIP) [file pntd.0013485.s007.zip › FocusGroups_Caregivers.docx]

FOCUS GROUP GUIDE WITH CAREGIVERS

I- Participant Introductions

- Participant Attendance List:

o Full Names

o Age

o Level of Education/Study

o Relationship to the PD-NTDs

I- Knowledge, Attitudes, and Practices of Caregivers Regarding Existing Policies, Regulations, Support Services, and NTDs

Knowledge (Discover the level of knowledge about the phenomenon and assess stakeholders' perceptions of both NTDs and support services)

o What existing laws and regulations regarding the protection of people with disabilities are you aware of?

o What care and support policies and mechanisms are you aware of?

o Are you aware of existing healthcare, rehabilitation, and reeducation services? List them

o Are you familiar with the organizations for people with disabilities operating in the country and in the pilot area?

o What are your relationships with these organizations as a support person?

o What do you think are the causes/sources/origins of NTDs?

o What are the consequences of the NTDs that you are aware of?

o Describe the symptoms of NTDs

o Describe the modes of transmission of NTDs

o What are the appropriate treatments for NTDs?

o What do you think are the specific needs of people with disabilities?

Attitudes

- What judgments (favorable or unfavorable) do you have about people with disabilities? ………………………………………………..
- What is your state of mind regarding the situation of your relative who is affected by NTDs? (Defeatist or optimistic attitude) ……………………………………………………………..

o Stigma / Discrimination

- Self-stigma/Perceived vulnerability:

Do you think you are vulnerable in the same way as your disabled parent? Justify your answer……………………

- Experienced stigma:

Can you describe situations of stigma experienced by your relative who is affected by NTDs and yourself?.....................................................................................................................................................

- Perceived stigma:

Can you describe situations of stigma perceived by your relative who is affected by NTDs and yourself? ......................................................................................................................................................

- Social participation (perceived advantages and obstacles related to disability):

Does the PD-NTD whom you care for participate in community or association activities? If so, what advantages do you and this person derive from them? If no, what are the obstacles to their participation?………………………………………………………………………………

Self-confidence and predisposition to change:

- Do you have confidence in their future, in their abilities and willingness to change? Justify your answer…………….

Cultural factors that characterize NTDs

Beliefs about NTDs and disability

- Do you think the causes or origins of NTDs are natural or mystical? Justify your answer………………………………………………….

Practices regarding NTDs

o Behaviors

Social behavior (social and professional integration)

- Does your relative who is affected by NTDs have an income-generating activity? ………………………………

- Do either of you belong to an association?...................................................

- Are you or your relative who is affected by NTDs active or involved in community activities?.............

Medical behavior (considering physical and mental health, monitoring treatment, etc.)

- Is your relative who is affected by NTDs under the care of a doctor? ……………………………........................

- Does he or she take his or her medication regularly? ……………………………………

Use of care / Treatment Regimen

What is the treatment regimen for the PD-NTDs?..................................................

What are the different types of treatment you use?..................................................................................................................................................

…………………………………………………………………………………………………

II- Suggestions and proposed solutions related to the situation of PD-NTDs

THANK YOU FOR YOUR COLLABORATION
