# Supplementary material for: People-centered strategies to mobilize people living with disabilities due to Neglected Tropical Diseases (PD-NTDs) to influence policy and programs: A mixed-methods study in Côte d’Ivoire
Source: PLoS Negl Trop Dis. 2025 Sep 8;19(9):e0013485. doi: 10.1371/journal.pntd.0013485 (PMC12431663; doi:10.1371/journal.pntd.0013485)
Supplement: S1 File — (ZIP) [file pntd.0013485.s007.zip › HealthRepsInterviewGuide .docx]

**Interview guide for representatives from mental health institutions; the three health regions; NTD focal points in health districts; and specialized care centers**

I- Introduction of the interviewee and the organization

1- Full name

2- Position and title

3- Number of years with the institution/health region/care center

4- Brief introduction of the institution/health region/care center

II- Missions and activities of the organization related to NTDs

1- What is the attendance rate of people with disabilities due to NTDs at your organization?

…………………………………………………………………………………………………………………

2- What are the existing mechanisms for psychosocial and economic support for people with disabilities due to NTDs?

……………………………………………………………………………………………………………………………………………………………

3- What do you think are the main problems facing people with PD-NTDs?

……………………………………………………………………………………………………………………………………………………………

4- Do you think there are sufficient rehabilitation facilities and skills? Justify your answer

……………………………………………………………………………………………………………………………………………………………….

5- Do you think people with disabilities are well organized? Justify your answer

……………………………………………………………………………………………………………………………………………………………

6- Do you think PD-NTDs are well informed and aware of NTDs and treatment services? Justify your answer

…………………………………………………………………………………………………………………………………………………………….

7- In your opinion, what are the main obstacles faced by PD-NTDs in terms of healthcare?

…………………………………………………………………………………………………………………………………………………………….

8- In your opinion, what are the main obstacles faced by people with disabilities in terms of access to education?

…………………………………………………………………………………………………………………………………………………………….

9- In your opinion, what are the main obstacles encountered by people with disabilities in accessing the labor market?

………………………………………………………………………………………………………………………………………………………………………………….

III- Suggestions and proposed solutions related to the situation of PD-NTDs ………………………………………………………………………………………………………………………………………………………………………………………………………………………………………………………………………………………………………………………………………………………………………………………………………………………….
